# Tetrapod V1R-like *ora* genes in an early-diverging ray-finned fish species: the canonical six *ora* gene repertoire of teleost fish resulted from gene loss in a larger ancestral repertoire

**DOI:** 10.1186/s12864-016-2399-6

**Published:** 2016-01-27

**Authors:** Veronika Zapilko, Sigrun I. Korsching

**Affiliations:** Institute of Genetics, University of Cologne, 50674 Cologne, Germany

**Keywords:** Lepisosteus oculatus, Phylogeny, Evolution, Olfactory receptor, Positive selection

## Abstract

**Background:**

Chemical senses serve a multitude of essential functions across the animal kingdom. Vertebrates employ four GPCR families to detect odors, among them the *v1r/ora* gene family. The V1R family is known to evolve rapidly in the lobe-finned lineage giving rise to tetrapods, but the homologous ORA family consists of just six highly conserved genes in teleost fish, with direct orthologs in the lobe-finned fish coelacanth. Thus, the teleost repertoire of six canonical *ora* genes was assumed to be the ancestral feature before the divergence of ray-finned and lobe-finned fish. So far, this hypothesis has not been tested with earlier diverging ray-finned fish.

**Results:**

We have newly identified the complete *ora* gene repertoires of five teleost species, and of spotted gar, a basal ray-finned fish, using thorough data mining and extensive phylogenetic analysis. The genomes of eight further teleost species were re-analyzed for their ORA repertoires. We report that direct orthologs of the six canonical *ora* genes (*ora1-6*) were present in all newly analyzed species, with faithfully preserved exon/intron structure and mostly preserved genomic arrangement in symmetric pairs for *ora1-4*. In four teleost species including medaka and cave fish we observe species-specific gene duplication events. Thus, the *ora* gene repertoire in teleost fish is not quite as strictly conserved as previously assumed. In fact, the examination of non-synonymous *vs*. synonymous substitution rates (dN/dS) shows pronounced negative selection in five of the six *ora* genes, but also rare occurrence of positive selection in *ora3* and *ora6*. Surprisingly, spotted gar possesses beyond the six canonical genes three additional genes, *ora7-8b*, orthologous to coelacanth genes *v1r07-10.* No orthologs for these genes were found in teleosts and cartilaginous fish.

**Conclusions:**

Early diverging ray-finned fish such as the spotted gar possess several *v1r*-like genes previously assumed to be restricted to the lobe-finned lineage, but now found to be already present in the most recent common ancestor of lobe- and ray-finned fish. Thus, the presence of just six canonical *ora* genes in many teleost species is not the ancestral feature of the ray-finned lineage, but caused by loss of two ancestral genes in teleosts.

**Electronic supplementary material:**

The online version of this article (doi:10.1186/s12864-016-2399-6) contains supplementary material, which is available to authorized users.

## Background

The chemical senses of animals deliver crucial information for essential tasks such as prey localization, predator evasion, reproduction and social behavior. Vertebrates possess a specialized olfactory sense, which employs four different GPCR families to detect odors. Generally, these families are characterized by a very dynamic evolution, with many gene gains and losses leading to distinctly different receptor repertoires even in closely related species [1, 2]. A notable exception is the ORA family, which consists of the same six genes in several distantly related teleost fish species, with very rare gene duplication events and a singular gene loss [3–5]. In all these species, ortholog *ora* genes are always more closely related than paralogs [3–5], and four of these genes have direct orthologs already in a cartilaginous fish [6, 7]. This is all the more remarkable, since the mammalian V1R family - which has originated from a single gene of the ORA family, *ora1* [8] - shows a very dynamic evolution characterized by frequent gene gains and losses even between closely neighboring species [9, 10]. V1R repertoires range between zero and close to 300 genes in tetrapod species [11]. The recent discovery of a family of 20 *v1r* genes already in coelacanths [8, 12] showed this tendency towards dynamic evolution to be present early in the lobe-finned lineage (which comprises coelacanths and tetrapods).

Thus, the difference in family properties - on one hand six canonical *ora* genes for teleosts, which are ray-finned fish, and on the other hand highly dynamic V1R repertoires for coelacanths and tetrapods - appeared to be a difference between the ray-finned and the lobe-finned lineage. Since orthologs of all canonical *ora* genes were also present in coelacanths and since all V1Rs originated from the ORA1 clade, the teleost repertoire of six canonical *ora* genes was assumed to be the ancestral feature before the divergence of ray-finned and lobe-finned fish. So far this hypothesis has been consistent with results in eight teleost species: two cichlids (*Haplochromis chilotes*, *Oreochromis niloticus)*, medaka (*Oryzias latipes*), two pufferfish (*Takifugu rubripes, Tetraodon nigroviridis*), salmon (*Salmo salar*), stickleback (*Gasterosteus aculeatus*), and zebrafish (*Danio rerio*) [3–5, 13, 14].

Although teleosts make up the bulk of fish species with ~29000 species [15], analysis of earlier diverging ray-finned fish would help to elucidate the evolution of the canonical ORA repertoire. We have investigated the ORA family of the spotted gar, *Lepisosteus oculatus*, a species that diverged early in the ray-finned lineage from teleosts [16]. Furthermore we have delineated the ORA family in five newly available teleost genomes, among them cod (*Gadus morhua*) as an early-diverging teleost, cave fish (*Astyanax mexicanus*), as close relative to zebrafish, platyfish (*Xiphophorus maculatus*), Amazon molly (*Poecilia formosa*) and mummichog, a species of killifish (*Fundulus heteroclitus*) as sister group of medaka. Finally we have re-analyzed the eight species mentioned above and present a comprehensive view of the ORA family in 14 ray-finned fish species.

Here we report that the canonical six *ora* genes are present in all 14 fish genomes analyzed. We notice six species-specific, i.e. evolutionarily late gene duplications, three species-specific intron gains, and rare instances of positive selection as suggested by dN/dS analysis. Surprisingly, we identified three additional monoexonic *ora* genes in spotted gar, *ora7-8b*, which have no teleost orthologs, but form two clades with V1Rs restricted to the lobe-finned lineage. Hence we propose that the ancestral ORA repertoire of bony fish consisted of at least eight genes, *ora1-8*, of which two genes were lost in teleosts (*ora7-8*), and seven genes in mammals (*ora2-8*). The full repertoire of eight ancestral *ora* genes is present in lobe-finned coelacanth and ray-finned spotted gar, and thus the canonical six gene repertoire so characteristic for teleosts should be considered a derived feature.

## Results

### The ora gene repertoire of an early-derived ray-finned fish encompasses three genes specific to the lobe-finned lineage

Previous research has shown the presence of six highly conserved, canonical *ora* genes in a total of eight teleost fish species ranging from zebrafish, an early-derived teleost, to several neoteleost species [3–5]. Orthologs of all six genes were identified in a lobe-finned fish [8, 12] suggesting this gene set to be the ancestral feature of lobe-finned as well as ray-finned fish. Multiple gene expansions appeared to be a feature characteristic for and restricted to the lobe-finned lineage that gave rise to tetrapods with their highly variable *v1r* gene repertoires [9–11].

**Fig. 1:**
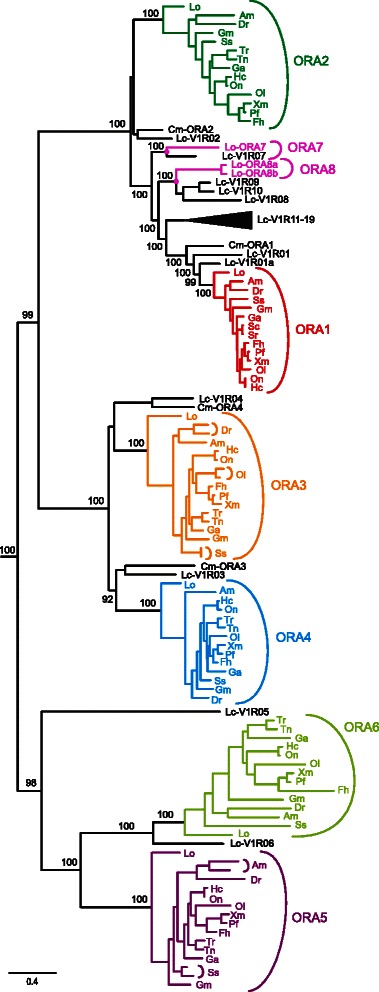
Phylogenetic tree of ORAs. Species are indicated by the initials of their Latin names, see Table 1 for full names. T2R receptors (not shown) were used as outgroup. Numbers indicate branch support. Magenta circles, ancestral genes for *ora7-8b* (spotted gar, *Lo*) and *v1r07-10* (coelacanth, *Lc*). Scale bar, number of amino acid substitutions per site

Here we have performed extensive searches in the genome of an early-diverging ray-finned fish, spotted gar. We report that spotted gar possesses three additional *ora* genes beyond the six canonical genes, *ora1-6* (Fig. 1). Two of the genes are incomplete, presumably due to fragmentary genome sequences in this region, but all three genes possess the expected amino acid motifs characteristic for *ora* genes and in the phylogenetic analysis are located within the ORA family with high branch support (Fig. 1).

Unexpectedly, these three novel *ora* genes belong to V1R clades of the lobe-finned lineage. Despite thorough data mining, no orthologs for these genes could be identified in teleosts and elephant shark. Extending the established numbering for ORAs the three genes were named *ora7, ora8a* and *ora8b.* ORA7 is a direct ortholog of the coelacanth receptor V1R07 (Fig. 1). The genes *ora8a* and *ora8b* result from a late, species-specific duplication event, and are orthologous to V1R08-10 of coelacanths. Thus, the most recent common ancestor (MRCA) of ray-finned fish and lobe-finned fish had not six, but at least eight ancestral genes, seven of which are present as single genes in coelacanths as well as spotted gar, whereas the eighth gene has undergone small independent expansions in both lineages (Fig. 1). Genes *ora7* and *ora8* appear to have been lost early in the teleost lineage. *Ora8* is ancestral to a large cluster of amphibian *v1r* genes (Additional file 1), whereas *ora7* appears to have been lost early in the tetrapod lineage, similar to *ora2, 4-6* [8].

**Fig. 2:**
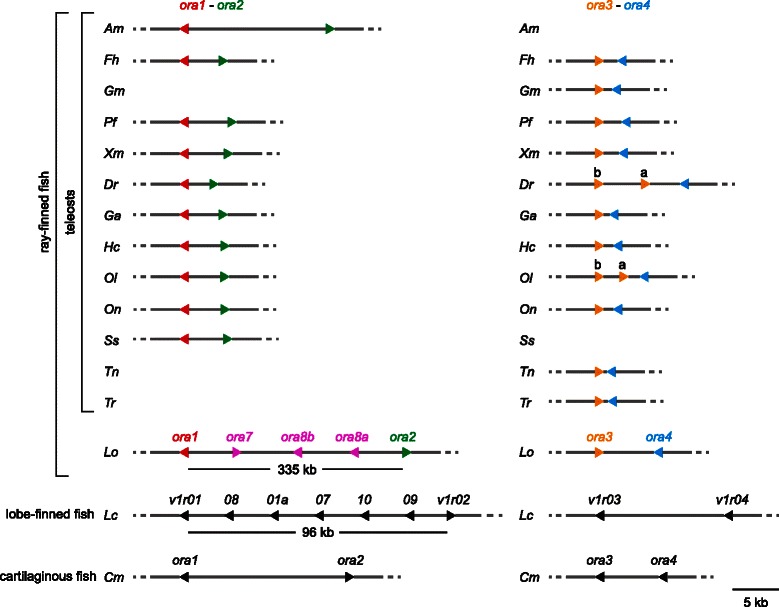
Genomic arrangement of the ora1/ora2 and ora3/ora4 gene pairs. Genes are represented by triangles and the intergenic regions are illustrated by continuous lines and drawn to scale. Triangles pointing right, + strand; triangles pointing left, - strand. Color code as indicated. Species are indicated by the initials of their Latin names, see Table 1 for full names. There is no genomic association for *Gm-ora1* and *Gm-ora2* as well as for *Am-ora3* and *Am-ora4;* for *Ss-ora3* and *Ss-ora4* this is unknown, since only small contigs are available [31]

*Ora7-8b* are located between the genes *ora1* and *ora2* on the chromosome, nearly syntenic to the arrangement of the orthologous coelacanth genes (Fig. 2). Interestingly, *ora1* forms an inverted pair with *ora7* in the spotted gar, and *ora2* forms an inverted pair with *ora8a* (Fig. 2). Such arrangement in inverted pairs is characteristic for teleost genes *ora1-2* and *ora3-4*, and might have some role in gene regulation, *cf*. [17].

### Rare species-specific gene duplications in teleost ora genes

We performed a comprehensive search of the ORA family in the genomes of 13 teleost species, five of which had not been analyzed before (cave fish, cod, Amazon molly, killifish, platyfish). Direct orthologs of all six canonical *ora* genes were identified in all five species (Fig. 1, Additional files 2 and 3). Our reanalysis of eight teleost genomes (zebrafish, stickleback, medaka, fugu, tetraodon, salmon, Lake Victoria cichlid, Nile tilapia) confirmed most of the previously found *ora* sequences [3–5, 13, 14, 18]. Tetraodon *ora6* and fugu *ora2* were reported as multiexonic [3], but in the currently available database versions a monoexonic prediction results in higher homology to orthologous genes.

We observe a duplication of the zebrafish, salmon and medaka *ora3* gene, and of the cave fish and salmon *ora5* gene (Figs. 1 and 2). All duplications are species-specific (Fig. 1), i.e. late evolutionary events after speciation had occurred. The duplicate genes neighbor each other in the genome, i.e. resulted from local gene duplications (Fig. 2 and Additional file 2). For zebrafish and salmon, our results are consistent with [5] and [4], respectively. Another duplicate gene reported for ORA1 [19] was not found in the genome. On average one gene duplication per three species occurs. This is much less frequent than what is observed for the highly variable mammalian V1R repertoires, but nevertheless shows the conservation of the *ora* gene family size not quite as strict as initial results suggested [3]. We have therefore investigated the degree of conservation for two other features, the exon/intron organization and genomic arrangement, and the ratio of synonymous *vs*. nonsynonymous mutations as a measure for the selective pressure on the six canonical *ora* genes.

### Intron gains in the ora gene family of ray-finned fish

In previous analyses *ora1-2* and *ora5-6* genes were found to be generally intronless, whereas *ora3* and *ora4* possess three and one intron, respectively [3–5, 13, 14, 18]. Additional introns had been reported for fugu *ora2*, zebrafish *ora4*, tetraodon *ora6* and salmon *ora6* [3, 4].

**Fig. 3:**
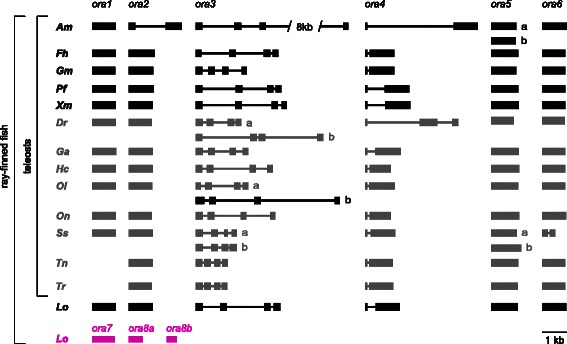
Genomic structure of ora genes from ray-finned fish. Rectangles, predicted exons; lines, predicted introns. Newly identified *ora1-6* genes, black; *ora7-8b*, magenta; previously published genes, gray. All elements are drawn to scale, if not stated otherwise. Species are indicated by the initials of their Latin names, see Table 1 for full names

We used the GeneWise algorithm [20] to predict the exon/intron borders of all *ora* genes in all 14 species investigated (Fig. 3). We confirm the previous results with two exceptions, fugu *ora2* and tetraodon *ora6.* Both now are predicted as monoexonic, see above. Furthermore we report a novel intron in cave fish *ora2* (Fig. 3)*.* Taken together this amounts to three intron gains (zebrafish *ora4*, salmon *ora6*, and cave fish *ora2*) in a total of 14 species ranging from an early-derived ray-finned fish (spotted gar) to several neoteleost species. No intron losses were observed. Considering that very few intron gains are expected in the vertebrate lineage [21] this shows notable evolutionary dynamics.

All five newly analyzed species and Nile tilapia show a conserved exon/intron pattern: *ora1-2, 5-6* are monoexonic (except cave fish *ora2*), *ora3* has three introns, and *ora4* one intron (Fig. 3). The exact borders of these introns are strictly conserved in all species, including the early-derived ray-finned spotted gar (Additional file 4), consistent with a common origin of these introns early in the evolution of the ray-finned lineage, since they are absent in the coelacanth orthologs [8].

### Genomic arrangement of ora1/2 and ora3/4 gene pairs is not always preserved

*Ora1* and *ora2* are arranged head-to-head in the genomes of previously analyzed teleost species [3–5], whereas *ora3* and *ora4* exhibit a tail-to-tail genomic orientation [3, 5]. Interestingly the head-to-head arrangement for the *ora1/ora2* gene pair is already present in the elephant shark, a cartilaginous fish, whereas the *ora3/ora4* gene pair has head-to-tail orientation in this species (Fig. 2). Both features may correspond to the ancestral situation since coelacanths also show head-to-head orientation for *ora1/ora2* and head-to-tail for *ora3/ora4* [8]. If so, the *ora3* gene must have flipped at some point in the ray-finned lineage resulting in tail-to-tail orientation.

We found the head-to-head arrangement of the *ora1/ora2* gene pair as well as the tail-to-tail arrangement of the *ora3/ora4* gene pair in most, but not all of the newly analyzed species, including the early-derived spotted gar. However, in cod *ora1* and *ora2* appear to have lost their close genomic association, and in cave fish the same is true for *ora3* and *ora4* (Fig. 2). Three of the four genes are present within large contigs, so that technical reasons for the association loss appear unlikely. Again, this analysis shows a somewhat less stringent conservation of genomic features within the *ora* gene family than previously assumed.

### Two sites in ora3 and ora6 show evidence of positive selection despite generally strong negative selection in the six canonical ora genes

The rate of nonsynonymous to synonymous (silent) nucleotide substitutions (dN/dS) is often used to estimate the selective pressure acting on particular genes. A dN/dS value below 1 is taken as evidence of negative selection, whereas dN/dS >1 is an indicator of positive selective pressure, i.e. a tendency towards diversification. Positive selection has been reported in several other chemosensory receptor gene families [8, 22, 23]. For *ora* genes previous analyses have yielded somewhat conflicting results. While a study of *ora* genes in five teleost genomes has found strong negative selection and no evidence for positive selection [3], similar to results with 13 salmonid species [19], other studies have reported positive selection in one of the *ora* genes in several closely related species [24, 25].

**Fig. 4:**
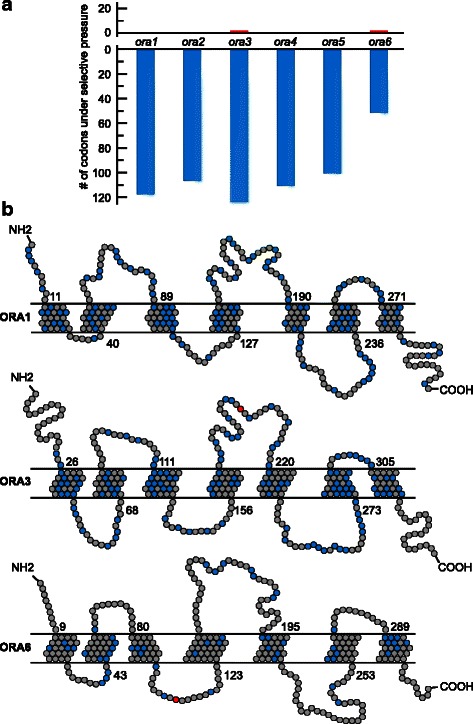
Strong negative and rare positive selection in ora genes from ray-finned fish. Predicted selective pressure for single codons is shown as consensus dN/dS values for fixed effects likelihood (FEL) algorithm and single likelihood ancestor counting (SLAC). **a** Numbers of negatively (*blue*) and positively (*red*) selected codons of each *ora* gene depicted as bar chart. 117, 106, 123, 110 and 51 negatively selected codons were identified in *ora1*, *ora2*, *ora3*, *ora4*, *ora5* and *ora6*, respectively. One positively selected codon was predicted for *ora3* and *ora6*. **b** Positions of sites with positive and negative selective pressure within the coding sequences are illustrated as snake plots for ORA1, ORA3 and ORA6. The schematic representations of these receptors were drawn based on the respective degapped codon-based nucleotide alignments, which were generated from the corresponding amino acid alignments

Here we have determined dN/dS ratios for each codon of all six canonical *ora* genes from 14 ray-finned species, including the early derived spotted gar. For higher stringency of results we required two different methods for estimation of dN/dS to agree in their prediction, *cf*. [26]. For five of the six *ora* genes, *ora1-5*, about one third of all codons was found to be under negative selection, whereas the frequency of such sites was much reduced in *ora6* (Fig. 4, Additional file 5). Negatively selected sites are distributed across transmembrane regions and loops, but appear to be less frequent in the N-terminal and C-terminal extensions (Fig. 4).

Interestingly, two positively selected sites were observed, one in an extracellular loop of ORA3 and one in an intracellular loop of ORA6 (Fig. 4). While the functional significance of these sites is so far not clear, this result again shows a less stringent conservation within the *ora* gene family than previously assumed, based on a considerably smaller data set [3].

## Discussion

Olfactory receptor families are among the fastest evolving gene families [2]. In particular, the V1R family is known to rapidly evolve in tetrapods [27], whereas the sister group in teleost fish, the ORA family, consists of a near constant repertoire of six canonical genes [3], all of which are present in the MRCA of ray-finned and lobe-finned fish, but with one exception have successively been lost in the lobe-finned lineage [3, 8, 12]. Thus, the six *ora* genes of teleosts were assumed to constitute the ancestral feature of both lineages. Here we have re-examined this assumption by delineating the ORA family in a larger set of teleost genomes than previously available, and in particular by investigation of an early-derived ray-finned fish genome, the first non-teleost ray-finned fish genome to be analyzed.

We find the canonical six *ora* genes in 11 teleost species (*ora1* is lost in both pufferfish [3]) and an early-derived ray-finned fish, but also occasional species-specific duplications of single *ora* genes. We also report a loss of the pairwise genomic arrangement, another characteristic feature of the *ora* gene family, for a single gene pair in two species. We observe two new species-specific intron gains within the ORA family. While there exists some controversy about the extent of intron dynamics in higher eucaryotes, it is generally accepted that there are very few intron gains in the vertebrate lineage [21]. A total of three intron gains in a small family in 14 species then shows considerable intron dynamics. Such intron dynamics appears to be characteristic for olfactory receptor gene families, as it has also been reported for *taar* genes [22] and *or* genes [23]. Finally, rare occurrence of positively selected sites in two *ora* genes again points to a somewhat more dynamic evolution of the *ora* gene family than previously assumed, based on a much smaller data set [3].

Unexpectedly we have identified three additional *ora* genes in spotted gar, which do possess coelacanth, but no teleost orthologs. Two of these genes, *ora8a,* and *ora8b* result from a recent gene duplication, which leaves two genes, *ora7-8* as ancestral genes already present in the MRCA of ray-finned and lobe-finned fish. We conclude that these genes most likely have been lost in the teleost lineage. Thus, the unusual conservation of the six canonical *ora* genes in teleost fish constitutes a secondarily evolved feature of the teleost lineage.

It may be expected that the very different evolutionary dynamics in tetrapod V1Rs *vs*. teleost ORAs reflect a difference in function. V1Rs have been reported as pheromone receptors [28]. So far a single ORA receptor has been deorphanized as receptor for a substance acting as reproductive pheromone [29]. Known reproductive pheromones of teleost fish encompass steroid and prostaglandin hormones and their metabolites [30] and it is conceivable that this double constraint on pheromonal and hormonal quality forces such substances to evolve less rapidly [30] than pheromones not being constrained by a concomitant hormonal function. However, a test of this hypothesis will require considerably more information on receptor/ligand pairing for *ora* genes in different species than currently available.

## Conclusion

**Fig. 5:**
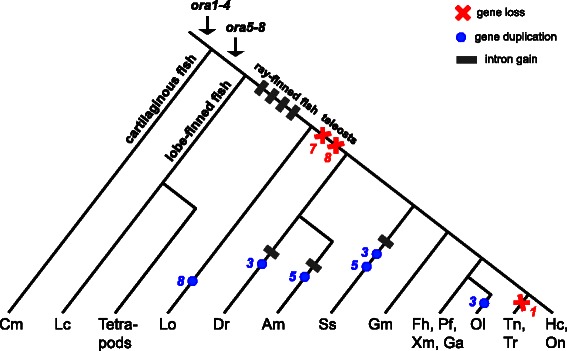
Evolutionary dynamics of ora genes. A taxonomy tree of cartilaginous, lobe-finned and ray-finned fish is shown. Species are indicated by the initials of their Latin names, see Table 1 for full names. Arrows indicate the minimal evolutionary age of genes as predicted by parsimony. *Red cross*, gene loss, red numbers indicate the gene lost; blue circle, gene duplication, *blue numbers* indicate the gene duplicated; *grey rectangle*, intron gain; only events in the ray-finned lineage are shown. The relative order of symbols within one segment is arbitrary. Note that ancestral genes ORA7-8 are lost early in the teleost lineage

We have performed a comprehensive analysis of the *ora* gene family. We delineated the ORA repertoire in thirteen teleost fish and one basal ray-finned fish species, and evaluated the presence of gene duplications, intron gains, variability in genomic arrangement, and positive selection (Fig. 5). We confirm the presence of six canonical *ora* genes in all newly analyzed species. Nevertheless, we observe noticeable evolutionary dynamics for this unusual olfactory receptor family in teleost fish. Furthermore, the presence of coelacanth and tetrapod *v1r*-like *ora* genes in a basal ray-finned fish shows the six canonical ora genes of teleost fish to be a secondarily derived feature that resulted from gene losses in a larger ancestral repertoire present in the MRCA of ray-finned and lobe-finned fish.

## Methods

### Data mining and phylogenetic analysis

ORA sequences of 13 teleost species (Table 1) and one early diverging ray-finned fish (spotted gar, *Lepisosteus oculatus*) were retrieved using BLAST searches in genomes available through NCBI, Ensembl and ASalBase [31]. Known ORA amino acid sequences of related species were used as queries. An E-value of 10^−10^ was used as cutoff, and all sequences with a length between 400 and 1250 nucleotides (after splicing) were considered further. Splicing predictions were made by comparing related protein sequences to genomic DNA sequences with the online-tool GeneWise [20]. In several cases the complete open reading frame had to be obtained manually. Predicted protein sequences and genomic locations or database IDs for nucleotide sequences of all ORA receptors identified here are listed in the Additional files 2 and 3, respectively. For spotted gar *ora8a, ora8b,* and salmon *ora6* only partial sequences could be retrieved from the databases.

**Table 1:** Nomenclature of species used in phylogenetic analysis

Abbr.	Latin species name	Vernacular name	Related species
*Am*	*Astyanax mexicanus*	Mexican cave fish	Dr
*Cm*	*Callorhinchus milii*	Elephant shark	—
*Dr*	*Danio rerio*	Zebrafish	Am
*Fh*	*Fundulus heteroclitus*	Mummichog (a killifish)	Pf, Xm, Ol
*Ga*	*Gasterosteus aculeatus*	Stickleback	—
*Gm*	*Gadus morhua*	Atlantic cod	—
*Hc*	*Haplochromis chilotes*	Lake Victoria cichlid	On
*Lc*	*Latimeria chalumnae*	African coelacanth	—
*Lo*	*Lepisosteus oculatus*	Spotted gar	—
*Ol*	*Oryzias latipes*	Medaka	Pf, Xm, Fh
*On*	*Oreochromis niloticus*	Nile tilapia	Hc
*Pf*	*Poecilia formosa*	Amazon molly	Xm, Fh, Ol
*Sc*	*Sebastus caurinus*	Rockfish	Sr
*Sr*	*Sebastus ruberrimus*	Rockfish	Sc
*Ss*	*Salmo salar*	Atlantic salmon	—
*Tn*	*Tetraodon nigroviridis*	Spotted green pufferfish	Tr
*Tr*	*Takifugu rubripes*	Japanese pufferfish (fugu)	Tn
*Xm*	*Xiphophorus maculatus*	Platyfish	Pf, Fh, Ol

The final inclusion criteria for candidate *ora* genes were firstly, a position within the ORA clade in the phylogenetic analysis, secondly, the presence of amino acid motifs characteristic for the Ora family [3], and thirdly, for the full length sequences, the prediction of seven trans-membrane domains. Transmembrane regions were predicted for multiple aligned sequences using PRALINE [32].

For phylogenetic analysis published sequences from elephant shark, African coelacanth, and two rockfish species (Table 1) were included [6, 8, 12, 25]. Sequences were aligned with MAFFT 7 [33], an online version of the multiple alignment tool MAFFT [34], using the E-INS-I strategy with the default parameters. The multiple sequence alignment was manually edited using Jalview [35] to remove regions with gaps in over 90 % of sequences. The phylogenetic tree was calculated using a Maximum likelihood algorithm, PhyML-aLRT with SPR setting for tree optimization and chi square-based aLRT for branch support [36] available online [37, 38]. Branch support above 80 % was considered significant. *T2r* genes of zebrafish, stickleback, spotted green pufferfish and coelacanth served as outgroup (see Additional file 3). The tree was drawn using an online version of TreeDyn [39].

### dN/dS analysis

The dN/dS ratios for the individual codons of the different *ora* family members were calculated using single likelihood ancestor counting (SLAC) described in [40], as well as the fixed effects likelihood method (FEL) that directly estimates nonsynonymous and synonymous substitution rates at each site [41]. Both methods were used as implemented on the datamonkey server [41]. Codon based nucleotide alignments were generated with PAL2NAL [42], and regions with gaps in over 90 % of sequences were removed using Gap Strip/Squeeze v2.1.0 [43]. The salmon ORA6 sequence is incompletely predicted (three transmembrane domains are missing) and was not included in the analysis. ORA1 from cod, a full length sequence, was also excluded from the analysis due to incomplete sequencing.

## Availability of supporting data

The data sets supporting the results of this article are included within the article and its additional files.

## Additional files

Additional file 1:
**A phylogenetic tree containing all sequences shown in Fig.** 1 **plus 89 mouse V1R and 15 frog V1R sequences.** Fish species are indicated by the initials of their Latin names, see Table 1 for full names. (PDF 481 kb) Additional file 2:
**A table containing gene names, protein sizes, genomic locations, Genbank IDs, NCBI reference sequences, first publications and previously reported names of** ***ora/v1r*** **genes of ray-finned fish, elephant shark and African coelacanth.** (XLS 44 kb) Additional file 3:
**A list of ORA/V1R and T2R protein sequences of ray-finned fish, elephant shark, and African coelacanth in fasta format, which were used in construction of the phylogenetic tree shown in Fig.** 1 **and the corresponding tree file in Newick format.** (PDF 173 kb) Additional file 4:
**A multiple sequence alignment of** ***ora1-8*** **genes of ray-finned fish with exon/intron boundaries marked in color.** Species are indicated by the initials of their Latin names, see Table 1 for full names. (PDF 70 kb) Additional file 5:
**Tables containing dN/dS values for all codons of** ***ora1-6*** **genes of ray-finned fish.** (XLS 207 kb)
