# Supplementary figures and images for: Tetrapod V1R-like ora genes in an early-diverging ray-finned fish species: the canonical six ora gene repertoire of teleost fish resulted from gene loss in a larger ancestral repertoire
Source: BMC Genomics. 2016 Jan 27;17:83. doi: 10.1186/s12864-016-2399-6 (PMC4728799; doi:10.1186/s12864-016-2399-6)

0.4

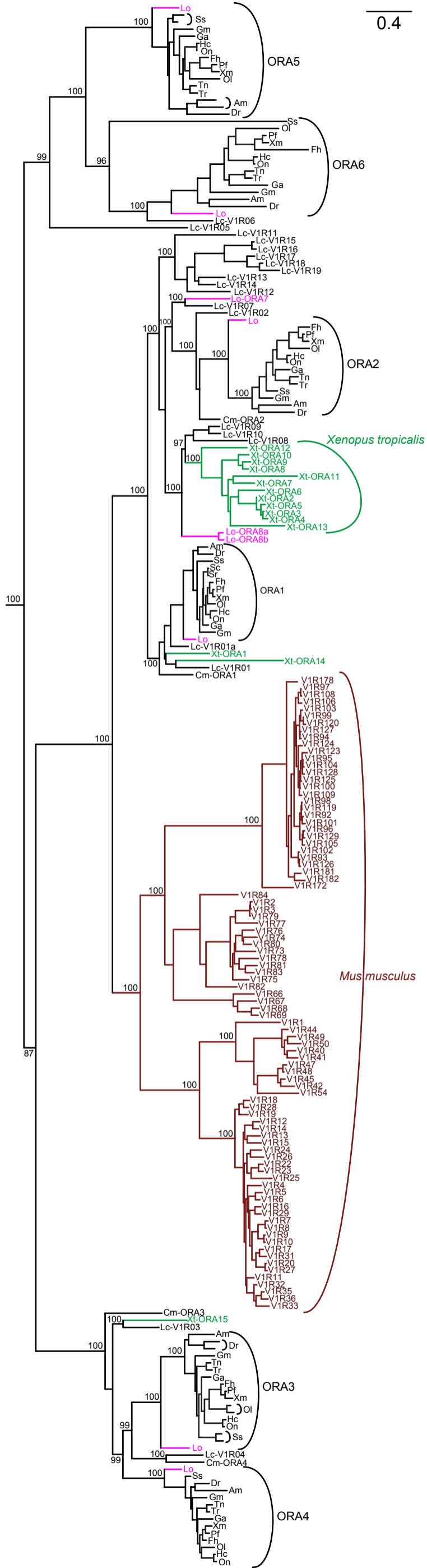

Supplement: Additional file 1: — A phylogenetic tree containing all sequences shown in Fig. 1 plus 89 mouse V1R and 15 frog V1R sequences. Fish species are indicated by the initials of their Latin names, see Table 1 for full names. (PDF 481 kb) [file 12864_2016_2399_MOESM1_ESM.pdf]
