# Supplementary material for: Tetrapod V1R-like ora genes in an early-diverging ray-finned fish species: the canonical six ora gene repertoire of teleost fish resulted from gene loss in a larger ancestral repertoire
Source: BMC Genomics. 2016 Jan 27;17:83. doi: 10.1186/s12864-016-2399-6 (PMC4728799; doi:10.1186/s12864-016-2399-6)
Supplement: Additional file 3: — A list of ORA/V1R and T2R protein sequences of ray-finned fish, elephant shark, and African coelacanth in fasta format, which were used in construction of the phylogenetic tree shown in Fig. 1 and the corresponding tree file in Newick format. (PDF 173 kb) [file 12864_2016_2399_MOESM3_ESM.pdf]

Additional file 3, content:

Part 1 Ray-finned fish ORA protein sequences. 2
Part 2 African coelacanth V1R protein sequences. 14
Part 3 Elephant shark ORA protein sequences. 17
Part 4 Teleost and African coelacanth T2R protein sequences. 18
Part 5 Tree file in Newick format for sequences shown in Figure 1. 21
Part 6 References 24

All parts: Species are indicated by the initials of their Latin names, for full names (Latin and vernacular) see Table 1.

Part 1 Ray-finned fish ORA protein sequences.

Novel sequences reported in this study are marked cyan, corrected sequences are marked yellow. Published sequences for zebrafish, *Danio rerio*, stickleback, *Gasterosteus aculeatus*, Lake Victoria cichlid, *Haplochromis chilotes*, medaka, *Oryzias latipes*, Nile tilapia, *Oreochromis niloticus*, Atlantic salmon, *Salmo salar*, spotted green pufferfish, *Tetraodon nigroviridis*, Japanese pufferfish, *Takifugu rubripes,* and rockfish, *Sebastus caurinus* and *Sebastus ruberrimus* are from [1-5] and marked gray.

>Am-ORA1

MDLCITIKGVSFLLQTGLGIMGNMLVLAAYGHIALVEPRLQPVDQIMAHLAFANLMLLLTRGVPQTMTVFGLRHL
LNDSGCKVVIYTYRITRALSVCFTCMLSVFQALTIAPAGGPRLARLKARLPQLVAPTFAGLWLLNMAVCIAAPFF
SVAPRNGTVPPFTLNLGFCHVDFRDNLSYVINGAAVSARDFSFVGLMLGSSGYILVLLHKHSRQVRAIRRSQGGS
MEMRAAKTVVMLVVLYAVFFGIDNVIWIYMLTVAQVPAVVADMRVFFSSCYATLSPFLMISSNKKLKERMVCAAG
GEQKQDAAEDTDKTNIK

>Fh-ORA1

MDLCVTIKGVSFLLQTGMGILGNGVVLLAYASIICTEPKLLPVDMILCHLAFANLMLLLTRCVPQTMTVFGLKGL
LNDPGCKVVIYAYRVGRALSVCITCMLSVFQAATIAPAGPRLSRLKPTLPSLVLPTFAMLWFLNMAICVAAPLFS
MAPRNGTVPAFTLNLGFCHVDFRDNLSYVVNGVAVSGRDFAFVALMVGSSGYILLLLHRHSEQVRGIRRSHGSGA
ETRAAKTVVTLVVLYVVFFGIDNVIWIYMLTVSKVSPVVADMRVFFSSCYASLSPYFIISSNKKVKRKIVCAAEH
DQPLVDTQESNEK

>Gm-ORA1

MDLCVTIKGVSFLLQTGLGVLGNALVLLAYVHIAHGADHKLLPTDLILCHLAFSNLVLLLTRCVPQTMTVFGLHD
LLDDAGCKVVIYLYRITRALSVCLTCMLSVFQAATLAPDAPRLKAALPALVLPSFAGLWLLNMAVCIAAPFFSIA
PRNGTVPAFTLNLGFCHVDFRDYLSYVINGVAVSVRDFAFVLLMLLSSGYILLILHRHSRQVRSMRRGAAQGVET
RAAKTVVTLVVLYAVFFGIDNVIWIYMLTVAKVSPVVADMRVFFSSSYAFLSPYFIISSNKRIKGKLVCAVEQQQ
SPVETQTSNEK

>Pf-ORA1

MDLCVTIKGVSFLLQTGLGILGNVVVLLAYASIICTEPKLLPVDMILCHLAFANLMLLLTRCVPQTMTVFGLKDL
LNDPGCKVVIYAYRIGRALSVCITCMLSVFQAMTIAPAGPRLSKLKPVLPSLVLPTFAALWLLNMAICIAAPFFS
MAPRNGTIPAFTLNLGFCHVDFRDNMSYVINGVAVSGRDFAFVALMLGSSGYILLLLHRHSEQVKGIRRSHGSRA
ETRAAKTVVILVVLYVVFFGIDNVIWIYMLTVSKVSPVVADMRVFFSSCYASLSPFFIISSNKKVKRKIVCAAEH
DQPLVDTQESNEK

>Xm-ORA1

MDLCVTIKGVSFLLQTGLGILGNAVVLLAYASIICTEPKLLPVDMILCHLAFANLMLLLTRCVPQTMTVFGLKDL
LNDPGCKAVIYAYRIGRALSVCITCMLSVFQAMTIAPAGPKLSKLKPMLPSLVLPTFAALWLLNMAICIAAPFFS
MAPRNGTIPAFTLNLGFCHVDFRDNMSYVINGVAVSGRDFAFVALMLGSSGYILLLLHRHSEQVKGIRRSHGSRS
ETRAAKIVVTLVVLYVVFFGIDNVIWIYMLTVSKVSPVVADMRVFFSSCYASLSPYFIISSNKKVKRKIVCVAEH
DQPLVDTQESNEK

>Dr-ORA1

MDLCVTIKGVSFLLQAGLGILANALVLLAYAHIRLAEARLQPVDAILCHLALVDLLLLLTRGVPQTMTVFGMRNL
LDDTGCKVVIYTYRIARALSVCITCMLSVFQAVTVAPAAGPLLSGVKARLPQLLAPTFAALWFINMAVCIAAPFF
SVAPRNGTVPPFTLNLGFCHVDFHDNLSYVLNGVAVSVRDFAFVGAMLASSGFILLLLHRHRRQVRAVRRSQGST
METRAARTVLMLVILYSVFFGIDNVIWIYMLTVAQVPPVVAHMRVFFSSCYASLSPFLIISSNRKLKARMVCATS
EQERQAEDGKNSSGKN

>Ga-ORA1

MDLCVTIKGVSFLLQTGMGILGNTVVLLAYAQLIYAEPKLLPVDMILCHLAFANLMLLLTRCVPQTMSVFGLRDL
LGDPGCKVVIYAYRIGRALSVCVTCMLSVFQAVTLAPAGPRLSRLKPALPSLVLPTSAGLWLLNMAVCVAAPLFS

MAPRNGTAPAFTLNLGFCHVDFRDNLSYVINGVAVSVRDFAFVALMLGSSGYILLLLHRHSRRVRGIRRSQGGGA
ETRAAKTVITLVVLYAVXFGIDNAIWIYMLTVAKVSPVVADMRVFFSSCYASLSPYFIISSNKKVKAKILCAAEQ
DQPSVDNQETSDK

>Hc-ORA1

MDLCVTIKGVSFLLQTGMGVLGNTVVLLAYTHIVCTGPKLLPVDMILCHLAFTNLLLLLTRSVPPSMTVFGLKAL
LNDPGCKVVIYAYRIGRALSVCITCMLSVFQAVTITPTGPYLSRLKPSLPSLVIPTFAGLWLFNMAICISTPLFS
MAPRNGTVSAFTLNLGFCLVDFRDNLSYVINGVAISGRDFAFVALMVGSSCYILLLLHRHSHQMKGIHRSQGGGA
ETRAAKAVLTLVVLYVVFFGIENVIWIYMLTVEKVSPVVADMRVFFSSCYASLSPYFIISSNKKVKAKIVCTAEH
EQPSADTQDSNDK

>Ol-ORA1

MDLCVTIKGVSFLLQTGLGILGNSVVLLVYSHIMCTGPKLLPVDMILCHLAFANLILLLTRCVPQTMTVFGLKDL
LNDPGCKVVIYAYRIGRALSVCITCMLSVFQAVTIAPAGPFLSRLKLALSSLVFPTFVGLWLLNMAVCIAAPFFS
MAPRNGTVLPFTLNLGFCHVDFRDNLSYVINGVAVSVRDFAFVALMVGSSGYILLLLHRHSHQVRKIRRSHSSGA
ETRAAKTVLILVILYVVFFGIDNVIWIYMLTVSNVSPVVADMRVFFSSCYASLSPYFIISSNKKVKRKIVCAAEQ
DQPSVETQESNDK

>On-ORA1

MDLCVTIKGVSFLLQTGMGILGNTVVLLAYTHIVCTGPKLLPVDMILCHLAFANLLLLLTRCVPQTMTVFGLKDL
LNDPGCKVVIYAYRIGRALSVCITCMLSVFQAVTITPTGPYLSRLKPSLPSLVLPTFAGLWLFNMAICIAAPLFS
MAPRNGTVPAFTLNLGFCHVDFRDNLSYVINGVAVSGRDFAFVALMVGSSCYILLLLHRHSHQVKGIRRSQGGGA
ETRAAKTVLTLVVLYVVFFGIDNVIWIYMLTVAKVSPVVADMRVFFSSCYASLSPYFIISSNKKVKAKIVCAAEH
EQPSADTQDSNDK

>Ss-ORA1

MLDLCVTIKGMSFLLQTGLGFLGNTLVLLAYTQVVCSECRLQPVDIILCQLAFVDLILILTRCIPQTMTVFGLRD
LLNDPGCKVVVYSYRIARALSVCITCMLSVFQAVTIAPAGGPCLSRLKAQLPSLIVPTIAGLWLFNMAVCLAAPL
FSIAPRNGTVPAFTLNLGFCHVDFRDRLSYKINGVVVSTRDFAFVGLMLWSSGYILLLLHRHSHQVRSIRRSSQG
GGAETRAAKTVITLVVLYAVFFGIDNIIWVYMLTVDKVSPVVNDMRVFFSCCYACLSPFFIISSNKKVKSKLVCV
AADQEQPSVNTQDSNDKM

>Sc-ORA1

CITCMLSVFQAVTIAPAGPRLSRLKPALPSLVLPTFALLWFLNMAVCIAAPFFSMAPRNGTVPAFTLNLGFCHVD
SRDHLSYVINGVAVSTRDFAFVALMLGSSGYILLLLHRHSRQVRGIRRSQGGGAETRAAKTVITLVVLYVVFFGI
DNVIWIYMLTVPQVP

>Sr-ORA1

CITCMLSVFQAVTIDPAGPRLSRLKPALPSLVLPTFALLWFLNMAVCIAAPFFSMAPRNGTVPAFTLNLGFCHVD
FRDHLSYVINGVAVSTRDFAFVALMLGSSGYILLLLHRHSRQVRGIRRSQGGGAETRAAKTVITLVVLYVVFFGI
DNVIWIYMLTVPQV

>Lo-ORA1

MDLCITIKGVSFLLQTGLGILGNLLVLLAYAHIACSDGRVQPVDKILCHLAFANLLLLLTRCVPQTMTVFGLKDL
LNDSGCKAVIYAYRIARALSVCITSMLSVFQSIIIAPASSRWVGLKVRVSQLLFPSFAALWLINMAVCIAAPFFS
IAPRNGTVPEFTLNLGFCHVDFRDSLSYIINGVAVSGRDFIFVGLMVCSSGYILLLLHRHGKQVRQIRSPDQHSS
RNAAETRAAKTVVTLVTLYVVFFGIDNIIWIYMLTVAQVPPVIADMRVFFSSCYASLSPFLMITSNKKIKNKLSC
AAANPEQMSLNTEDSTRI

>Am-ORA2

MDLYFLTRGLLYLFLPVFGVPGNCAVIWAFLLALRQEGTLLPADAIVLHLACANLLVVSCRCVFEVFANFQVFNG
FNDPGCKGIYFIYRTFRGLSIWLTFTLSSYQCLSIAPPGSHWATLRSLFGRYLWLIFLLLWIINTSASAPTLVFA
VAARNDSKLLENSINIQFCFINFPSVFAKDANGALQVVRDVIPMSLMTTASFIILVFLYRHSRQVSNLRSGTGTG
GGGASAERRAAISVVVLVTFYVLMYGVDNGLWVYTLTVKQTLSSALISDLRIFFSMLFAAISPIIIIITTNMKVKK
QLL

>Fh-ORA2

MRGNHCLSGTRSASELPAFLPSAMPSDKLVRGWLFLSLAVVGIPGNIAVIVAFLLLILQECFLLAADAIVLHLAF

ANLLVVLVRCLLEALASFHLANVFGDVGCKAVIFIYRTSRALSIWLTFLLSAYQCLSIAPPGSSWASARVLVAQN
LPAVFFILWVLHTSMSVGAVLFSVSSRNYTAMTTSAVNAEFCYVNFRSDVLKEAYGAIQVSRDVVPMALMTLTSL
VILVLLYKHSQHLKGLRGAGSAGAGSCGAEQRAAKVVVVLVTVYVVLYGVDNGFWVYTLTVRNTLSSSLISDLRV
FFASLYAALSPLVIIASNRKVNGRLRCVAQERPLLGKTARLRSI

>Gm-ORA2

MPSEELIRAMLYLTLTVVGVPGNLAVIWAFLLALHQERRLLPADTILLHLASVNLLVVGVRCLLETLASFRLASV
FGDTGCKSVIFVYRTARSLSIWLTFVLSAYQCLSIAPPGSRWAAARALAARYMAAIFLALWLGNTCMSSAAVLFS
VGAGNGNGSSSLGSNGINVQFCVVRFPTRLSKDANGAVQVARDVVPMGLMATASLVILVFLYRHSRQVKGLRSGG
GGGRDGAERRAAKAVVALVTLYVGLYGVDNGLWVHTLTVRRTMGSSLVSDLRIFFSSLYAALSPAVIIATNRKVQ
RRLRCGRGEKHRGESATEATAVSTM

>Pf-ORA2

MIFYLYLLFSLSAGMPSNVNVRGMLFLSLTVVGVPGNMAVIVAFLLLVLQESWLLAADAIVLHLSCTNLLVVLVR
CLMETLASFHLANVFGDVGCKAVIFIYRTSRALSIWLTFLLSAYQCLSIAPPGSKWASVRILVAQNLPIVFLFLW
VLHTSTSAGAILFSVSSKNGTAVTTSAVNLEFCYVNFPSDIIKEVYGAIQVSRDVVPMALMTLTSLIILVLLYKH
SQHLKGLRGAGHAGSGTCGSKQRAAKVVVVLVTIYVVLYGVDNGLWVYTLTVRHTMSSSLISDLRVFFASLYAAL
SPLVIIASNRKVNSRLRCVAQEKPLLGKTAHLRSI

>Xm-ORA2

MPSNEDIRGMLFLSLTVVGVPGNMAVIVAFLLLILQESCLLAADAIVLHLSCTNLVVVLVRCLMETLASFHLANV
FGDIGCKGVIFIYRTSRALSIWLTFLLSAYQCLSIAPPGSKWASLRTLVAQSLPIVFVFLWVLHSSLSAGAILFS
VSSKNVTAVATSAVNVEFCYVNFPSDILKKVYGAIQVSRDVVPMALMTLTSLIILVLLYKHSQHLKGLRGAGHAG
SGTGGSKQRAAKVVVVLVTIYVVLYGVDNCLWVYTLTLRHTMSSSLISDLRVFFASLYAALSPLVIIVSNRKVNS
RLRCVAQEKPLLGKTAHLHSI

>Dr-ORA2

MIAEAVIRGLLFLSLVLVGVPGNTAVICGFILLVRREGRLSPADAIVLHLCSANLVVVSVRCLLEVLATFRIHNV
FDDAGCRAVIFLHRTARSLSIWLTFLLTALQCLSVAPPGSRRAAARALLARSLPAIFLALWLINTSMSVASLLYS
IGARNDSRLLQNAINVEFCFLSFPSRLARDANGAAQVARDVVPMVLMAAGSLVLLVYLVRQRRRVQGLRGTAGGA
AERRAAVTVVTLVSLYLLVFGLDNGLWVYTLTVSHTLSSALITDLRLFFTSLYTAVSPLLILVSNTRLRCGKQPE
TMH

>Ga-ORA2

MPSEMFVRGMLYLSLTVLGVPGNATVILAFLLLLYQERRLLPSDAIVLHLAFVNLLVVAARCLPETLASFRLSGI
FGDVGCKAVIFVYRTSRSLSIWLTFVLSAYQCLSIAPPGSRWAHLRVLLAQYLGLVFLILWLLNTCMSSAGILFS
FGTKNVTNLTNFDINVQFCYVNFPSKLSIQANGASQVGRDVVPMALMTLDSLIILVFLYKHSQQAKDLRGSRGGG
AERRAAKVVVALVTLYVVLYGVDNGLWVYTLTSRKAMESSLISDLRVFFASLYAALSPAVVIASNRKVNSRLRCD
VKRKPVEEKDTCLSTV

>Hc-ORA2

MASEVFVRGMLFLFLTVVGIPGNATVIVAFLLLLYQEKRLLAADSILLHLACVNLLVVVVRALTETLASFRLADI
FGDTGCKSVIFIYRATRGLSIWLTFLLSTYQCLSIAPPGSSWASVRALLGHYLAFVFLFLWVLNACMTTAAILFS
FSTKNETSPIDNGINVQFCYLNFPSKLSRDANGAIQVGRDVVPMALMTLASLIILVFLYKHSQQVKGLRSSGGGG
AGNSGAEQRAAKAVVALVTLYVVFYGADNGLWVYTLTVKKTMSSSLISDLRLFFGSLYAALSPLVIIASNRKVNS
RLGCVAHEKSAVEKIKNLSSM

>Ol-ORA2

MAHSFVFLEPFFCAGMPSDDLVRRGLYASLTIVGVPGNILVIMAFLLLSYEENRLLAAEAIVLHLTCANLLVVVV
RCLTETLASFHVVNVFGDAGCKGVIFIYRTSRGLSIWLTFLLSTYQCLSVSPPGSYWASVRVLLAQNLVFVFLFL
WVLNTTMSSGAILFSLSSKNDSSPINNAVNLEFCFVSFPSDLSKEIFGAVQVSRDVVPMALMTLTSVIILVLLYQ
HNQHMNDLHRNSNASGGRCGAKRRAAKVVVVLVTLYLGLYGVDCGLWVYTLTVKKTMSSSLISDLRVFFASMYAA
LSPLVIIVSNRKVNSRLMCISQKLA

>On-ORA2

MASEVFVRGMLYLSLTIVGIPGNATVIVAFLLLLYQEKRLLAADAILLHLACVNLLVVVVRALTETLASFRLADI
FGDTGCKSVIFIYRTTRALSIWLTFLLSTYQCLSIAPPGSSWASVRALLGHYLAFVFLFLWVLNTCMTTAAILFS
FSTKNETSPIDNGINVQFCYVNFPSMLSRDANGAVQVGRDVVPMALMTLASLIILVFLYKHSQQVKGLRSSGGGG

AGNGGAEQRAAKAVVALVTLYVVFYGVDNVLWVYTLTVKKTMSSSLISDLRIFFGSLYAALSPLVIIASNRKVNS
RLGCVAHEKSAVEKTKNLSSM

>Ss-ORA2

MQSEEVVRGMLYLSLTVVGVPGNTAVIVAFLLALYQEHQLLPADAIVLHLACANLLVVGVRCLLETLATFRLVNI
FGDTGCQGVIFVYRTSRSLSIWLTFVLSAYQCLSIATPGSRWASIRVLVARYLAVIFLTLWVINTSMSSAAIAFS
LGSRNDSVNMQHSINVQFCYVRFPTMQSKQVNGAVQVGRDVVPMGMMTLASLVILVFLYRYSQQVKGLRSSSGAS
GGAERRAAKAVVVLVTLYVVLYGVDNGLWVYTLTVRKTMSSSLISDLRIFFSSLYAALSPLVIIATNRKVNSRLR
CVVQERPVQDKATTLSTV

>Tn-ORA2

MIVVTIILRDLLSISPGMQSTEFVRGILYLSLAVVGAPGNTCVILAYFILLYQEKRLLPADVIILHLACANLLVV
VARCFLEFLASFRLALIFGDVGCKSVIFVYRTSRSLSIWLTFILSAYQCLCIAPLGSQLATLRMIVAKYLFYVFF
FLWLLTTTMSTAAILFSFSTQNGTNLVNNSINVQFCYVQFPSKLSKDANGAAQVGRDVVPMTLMTLASLIILAFL
YKNSQQVKGLRSSSSDRAEKRAAKAVVTLVSLYVLLYGVDNGLWVYTLTVREAMASSLISEMRIFFSSMYAALSP
IVIIVSNRKVNNILRCAGQEKHVQEKTT

>Tr-ORA2

MKDYGGIILRDLFSISTGMQSVEFVRGILYLSLTVVGAPGNICLILAYLILLHQENRLLPADVIILHLSCVNLLV
VVARCLLEFLASFHLAIIFGDVGCKSVIFVYRTSRSLSIWLTFILSAYQCLCIAPPGSQWTTLRIVFASYLFYVF
FFLWLLTTSMSSAAVLFSFGTQNDTNLINHSVNVQFCFVHFPSKMSRDANGAAQVGRDVVPMALMTLASLIILAF
LYKNSQQVKGLRSRDGGSGRAERRAAKAVVTLVTLYVLLYGVDNGLWVYTLTVREAMRSSLISDLRVFFSSLYAA
LSPIVIIISNRKVNSILRCAEQQKHVQ

>Lo-ORA2

MDPQVVIRGMLYLFLVVVGVPGNLAVIWAFCHIMRSERKLMPADAIVLHLAAVNLLVAAVRCSFEALAAFGVLYV
FNNTGCKTIIFIYRTSRSLSIWLTFVLSTFQCISIVPPGSRGYSIKSHAPRYLGGVFVFLWILNSWLSSAALAFA
VSSGDNSTRTQYGINIEFCIVNFPSSTWKNAVGAVQVARDAVPIFLMVAASLFILLFLYRHSQQVKGLRSAKRTQ
KESAESRAAKTVVTLVTLYVLFYGIDNGLWVYTLTVTQTLSTSLISDLRIFFASLYAAVSPLVIIASNKKVKSQL
GCMKTEKGPVSVDTVLSTV

>Am-ORA3

MWVNGTVTIIKATGGQLSSVPMALYMILVLLGIFGNAIVISVVGESILREPGGGRNSDMILVNMAFSNLMVSMTR
NMLLVISDTGLEVLPGKDWCQILMGVWVWLRSVNVWSTFFLSAFHFHTLRRTAPPITSLSGPRGLPRGILTGFGL
IWSSNLLYSVPAFIYSTSGGKNATETLMLVSSTTRPLLGCLWDFPSVYSGLAFATTSMVIHEIIPIVLMSVTNLG
SLLTLYAHGSKLHATNKSQQDPTMNRVPAERRAAKVILALILLFIVSWGASVISVNYFNYNRGASSTYLLVLARF
FNSLFIALSPLILAVGHRRLRQFFKSIISH

>Fh-ORA3

MGLRTPVSPAQSAFYIILVALGIVGNAIVIGVIGKSVMVDHGGGHNSDIIIINLAVSNFMVSVVRNVLLIVSDLG
IQMYSSKGWCQFLMGVWVWLRSVNVWSTFFLSAFHLHTLKRVAPTIGNIQGPWSTYRTLLLSLGVIWILNLLYSI
PAHIFSTSGNENTTETLMLVSSTTRPLLGCVWNFPSNYSGLAYATTSMAIHEIFPIILMAVTNMTSLYTLYTHGR
SRSSVQDAPVIKRVPAERRAAKVILALVMLFIVSWGTSIISVNYFNYNRGSSAEYLLVIARFANIIFIAMSPVVL
AFGHRRLRSCMKSSVSD

>Gm-ORA3

MSEAEAELVGMGLRTDASPVQTTFYILLVLFGIVGNTTVIGVIGHSVLMNPGVGRNSDIIIINMAVSNLMVSVLR
NALLVISDIGIALYSSKECCQFLMGVWVWLRSVNVWSTLLLSAFHFHTLRRVAPPLGNLHGPRGLPKLLLLGLGL
IWVLNFLYSIPAHVFSINGNQNSTETLMLVSSTTRPLLGCIWNFPLKNGLLYATISMVIHETLPIVLMAFTNAGS
LYSLYAHSKMRSMVNDVHVIKKVPAERRAAKVILALIMLFIASWGTSIISVNYFNYNRGQSAEFLLVIARFANIF
FIAMSPIILSIGHRRLRSFFTSLV

>Pf-ORA3

MQQESTTKLLGMGLRTGVSPAQSAFYIILVALGIVGNSIVIGVIGKSVLMDRGPGHNSDIIIVNLAVSNLMVSIV
RNLLLIISDLGFKLYSSKGWCQFLMGVWVWLRSVNVWSTFFLSAFHLHTLKRVAPTIGHLQGPWSTYRTLLLSLA
IMWILNLLYSIPAHIFSTSGNENTTETLMLVSSTTRPLLGCVWNFPSNYSGLAYATTSMAIHEIFPIILMAVTNM
TSLYTLYTHGRSRSSVQDAPVLKRVPAERRAAKVILALVLLFIVSWGTSIISVNYFNYNRGSSAEFLLVIARFAN
IIFITMSPVVLAIGHRRLRSCMKSSVSD

>Xm-ORA3

MEQETTTKLVGMGFRTAVSPAESDFYIILVALGIVGNSIVIGVIGKNVMMDRGPGHNSDIIIVNLAVSNLMVSIM
RNLLLIISDLGFKLYSSKGWCQFLMGVWVWLRSVNVWSTFFLSAFHLHTLKRVTPTIGDLQGPRSTYRTLLLSLA
IIWILNFLYSIPAHIFSTSGNENTTETLMLVSSTTRPLLGCVWNFPSNYSGLAYATTSMMVHEIFPIILMVVTNM
MSLYILNTYGRSRGSVQDVPVLKRVPAEKRAAKVILALVMLFIVSWGTSIISINYFNYNRGSSAEFLLVVARFGN
SIFITMSPVVLAIGHRRLRSCMKSSVSD

>Dr-ORA3a

MAPQKKPVNISQRITSSPFYIMLYVVLVLLGNAGNTTVIAVVGQSLLQETGTVRSSDVILVNMAFSNLMVSLLRN
TVLMVSDLGVEIFLSRDMCQFMMGLWVWVRSANVWSTFFLSAFHFQTLRRVAPPVINLHGPRGPPLSLILGFCLI
WSLNLIYSIPAFIFSKNGNENSTETLMLVSSTTRPLLGCIWDFPSAYSGLAFATSSMILHESIPICLMNITNLGS
LCTLYAHGHKRTVASQGEDAPVVSRIPAERRAAKVILALNILFISSWGTNVISVNYFNYNRGQSTEFLLIIARFV
NMSFIAFSPIILAVGHRKLRAFIKSVLSHMI

>Dr-ORA3b

MATTAKPLTVSQRALSSPLYIAFYVILVLLGNLGNSLVIGVVGEGLLREPGVARSSDIILVNMALSNLMVSLTRN
SLLVISDMGVQVFLNRNWCRFMMGIWVWVRSANVWSTFFLSAFHFQTLRRVAPPVSNVHGHPGPPRSLIFGLCLI
WSLNLIYSIPAFIFSKNGDANSTETLMLVSSTTRPLLGCIWNFPSAYSGLAFATSSMILHESIPICLMSITNMGS
LLALYAHGEARRAAKKSSDAPVVSRIPAERRAAKVILALNILFILSWGTSVISVNYFNYNRGSSTDWLLIAARIG
NITFIALSPIVLAVGHRRLRAFLASILTHSIALCRHLWTYKLQK

>Ga-ORA3

MAEHIGEDGESTLIGMGLRVSVSPVQTAFYIMLVTLGILGNFTVVGVIGKSIATDHVGGRNSDIIIINMALSSLL
VSVMRNIPLVISDIGLELYSSKEWCQVLMGLWVWLRSVNVWSTLFLSAFHLQTLRRVAPTAVSRNGPRGLPKTLL
LSLTLIWLLNLVYSIPAHIFSTSGDVNSTETLMLVSSTTRPLLGCVWNFPSSYSGLAYATTSMVIHETIPIILMA
ITNLGSLYTLYTHSRVRSTDAPVIKRVPAERRAAKVILTLIMLFIVSWGTSIISVNYFNYNRGSSAEYLLIIARF
ANIIFIAMSPIVLTFGHRRLRSFVKSTLSH

>Hc-ORA3

MSSLSDQRKDLAGMRMHIFVSPAQTAFYIILVIMGILGNTTVILVIGKSIILEHNWGRNSDIIIVNMAMSNLLVS
LLRNTLLIISEFGLQIYTAKGFCQLLMGMSVWLRSVNAWSTLFLSAFHLQTLKRVAPGATNGPRGAPKTLLMCLG
LIWIGNLIYSIPAHIFSSNGNKNTTETLMLVSSTTRPLLGCVWNFPSTIGLAYATTSLVIHEMIPIILMAITNLT
SLYTLYTHGRNPQKDAPVLKRVPAEKRAAKVILTLILLFILSWGTSVISVNYFNYNRGSSADYLMVIARFANIIF
IALSPVILAVGHRQLRSCIKSTLVH

>Ol-ORA3a

MSALQAVFYAILVVLGVLGNTTVIVVVGKSVIQDRRVAHNSNIIIINMAASNLMVSVMRNILLVMSDFGIQLFLS
RERCQFLMGVWVWLRSVNVWSTFYLSVFHLQTLRRVAPSVGNLQASRGVPKTLLLNLLSIWILNLLYSIPAHIFS
TNGNANSTETLMLISSTTRPLLGCVWNFPSSYSGLAYATTSMVIHETLPIVLMTVTNLSSLYTLHTYGRTRKSVQ
DAPVVKRVPAEKRAAKVILILVLLFTVSWGTSVISVNYFNYNRGTSSEFLLVIARFAQILFIALSPAVLAVGHRG
LRSCIKSSLTY

>Ol-ORA3b

MSAVSRMKTQLDLRPADEAAAKIKDFVGPEGRDENPKHVGIGLQVPVSDVQIVCHVIMVVLAILGNATVIVVIGK
SVIQDRRVAHNSNIIIINMAVSNLMVSIMRNILLIVSDFGIQLFLSRERCQFLMGVWVWLRSVNVWSTFYLSVFH
LQTLRRVAPSVGNLQASRGVPKTLLLNLLSIWLLNLLYSIPAHIFSTNGNANSTETLMLISTTTRPLLGCVWNFP
SSHSGLAYATTSMVIHEALPILLMTATNLSSLYMLHTYSRTRTRTSIQHAPVIRGVPAERRAARVILILVLLFVV
SWGTSVISVNYFNYNRGTSSEFLLVIARFAQILFIALSPAVLAVGHRGLRSCIKSSLTY

>On-ORA3

MSSLSDQRKEPDLAGMGMRIFVSPAQTAFYIILVIMGILGNTTVILVIGKSIILEHNWGRNSDIIIVNMAMSNLL
VSLLRNTLLIISEIGLQIYTTKGFCQLLMGMSVWLRSVNAWSTLFLSAFHLQTLKRVAPGATNGPRGVPKTLLVC
LGLIWIGNLIYSIPAHIFSSNGNKNATETLMLVSSTTRPLLGCVWNFPSTSGLAYATTSLVIHEMIPIILMAVTN
LTSLYTLYTHGRNPRKDATVLKRVPAEKRAAKVILTLIILFILSWGTSVISVNYFNYNRGSSADYLLVIARFANI
IFIALSPVVLAVGHRQLRSCIRSTLVR

>Ss-ORA3a

METPMPELKELEPVGVGLRVTTSPTQTTFYIILVLLGIVGNTTVIGVMLDSVFKDPSGVRNSDIILMNMALSNLL
VSVLRNVLLVISDLGLELNTSRDGCHVLMGVWVWLRSVNVWSTLFLSAFHFQTLRRVAPPPGTVHGPRRPPKTLL
ISLGLIWFLNLIYAVPAHIYSTKGNKNSTEILPIILMAITNLGSLYTLYTHGRTHNPAHMTQDAPVIKRIPAERR
AAKVILALIVLFIGSWGTSIISINYFNYNRGLSAGFLLVIARFANTIFIAISPIVLALGHRRLRAVIKYFLTH

>Ss-ORA3b

METPMPELKELEPVGVGLGVNRYPFQNALYIIFVLLGIVGNATVVGVISESVFKDPSGGRNSDIILINMALSNLL
LSLLRNILLVISDLGLELNTSRDGCHVLMGVWVWLRSVNVWSTLFLSAFHFQTLRRVAPPSGTVHGPRRPPKTLL
ISLGLIWFLNLIYAVPAHIYSTKGNKNSTETLMLVSSTTRPLLGCVWNFPSSYDTLAYTTTSMVIHEILPVILMA
ITNLGSLYTLYTHGRTRNPAHMTQDAPVIKRIPAERRAAKVILALTILFIVSWGTSIISINYLNYYKGSSATFLP
VIARFANSIFIAISPIVLALGHRRLRAVIKSFLTH

>Tn-ORA3

MAANTEDDTEVVGMGLRVSVSPVQTASYIFLVLLGILGNTTVVGVIGKSILMDRGGGRNSDIIIVNMALSNLLVS
LMRNTLLILSDLGLEMYSSKEWCRFLMGVWVWLRSVNVWSTLFLSAFHFQTLRRVAPVAGPVQGARGAPKILLIN
LFLIWFINLIYSIPAHIFSTSGNINSTETLMLVSSTTRPLLGCIWNFPSRYSGLAYATTSMVLHETVPIVLMALT
NLGSLYTLYTHNGMRSSVQEVPIKKRVPAERRAAKVILALIMLFIISWGTSVISVNYFNYNQGSSAEFLLVIARF
ANIIFIALSPVVLAVGHRGLRSFFKSLLAH

>Tr-ORA3

MAVNSMDEDDMELVGMGLRVSVSPFQTAFYIFLVLMGILGNATVVGVIGKSIIMDRGGGRNSDIIIVNMALSNLL
VSLMRNMLLILSDIGLEMYSSKEWCQFLMGVWVWLRSVNVWSTLFLSAFHLQTLRRVAPMAVNVTGSRGAPKILL
MNMFLIWFINLLYSIPAHVFSTSGNINSTETLMLVSSTTRPLLGCIWNFPTRFSGLAYATTSMVLHETVPIVLMA
LTNLGSLYTLYTHNGMQSSVQDAPVIKRVPAERRAAKVILALIMLFIASWGTSIISVNYFNYNQGSSAEFLLVIA
RFANIIFIAMSPAVLAVGHRGLRTFFKSLLSH

>Lo-ORA3

MGEQTNKVVTVLLKAAIPAQNALYGLLVMLGIVGNGLVMGVVGRGLVKEGLARQHSDIILLNLVLSNLLVSLVRN
IPLLLADVGLQLFTSPGCCQFLMFMWVWLRSVNVWMTMCLSAFHFLTLCRLGPVVPAGPHGPRASLQRLLLVLAL
IWSLNLLYSFPGFFFSTQGGRNSTEELMLVSSTTRPLLGCVWSFPSRRGGLAYATTSLVLHELLPILLMVATNLG
TLHTLARHGRSQRAGETTLTRRIPAERRAAKVVLVLIMLFIISWGASVLSVNYYNYNRGPSTEFLLVMARFTNSL
FIAFSPLVLLAGHSRLKAIFRVIADHVHSFCFSQRCLKKSC

>Am-ORA4

MELLTIEAILFGFLVFSGILGNMLVIYAVFQCALDNPSHHLSPSDIILLNISMANLLTSMFRTIPIFISDLGLKV
SLDTNWCRVFMLLWVWWRAVGCWATLTLSAFHYATLKRKRVSTCPQALRKDRRLTWGALGLVWGTNLLFSIPASV
FTSHVHGNATTEVMVISCTTRPLLGCMWNFPTREQGYAFAAASMALNEVLPLVLMVGTNLATLHTLAKHIRAVAA
GPEMASGHSNSEKKAGHVIMALVTLFVVCWVLQVAAVTYYNYERGKHTDSLLTVSQFSSSLFVGFSPMVVALGHG
KMRKKIMGMLQRWLRKALCRDAEEHKRAPEISSTINSTITQKQTHH

>Fh-ORA4

MSEILTVDAILFGLLVFSGILGNILVIHVVFQSALESPSRRLPPSDTILVHLSLANLLTSLFRTVPIFVSDLGLD
VSLSAGWCRLFMLLWVWWRAVGCWVTLTLSFFHCATLRRHHVSLGPLTLQRERRRVWVVLALVWGANLAFSVPAL
LYTTHVQSNATVELMVISCTTRPLLGCVWEFPSSQQGSAFASASLALNEVLPLVLMVCTNLATLHSLAKHIRAVT
DSGSHGELDKHVSTERKAAHVIMSLVSLFVVCWVLQVAAVTYYNHDRGHHAEGLLTVAHFSASLFVGFSPLVVAL
GHGKLRRKIKSMILMWTDGPRGPESRSVSASPTTKGKQTCFEAQKEIQVIKVKAKVKSQR

>Gm-Ora4

MAEVLTVDAILFGLLVFTGIMGNIMVMHTVCQSAMQSQSRRMPASDTILVHLSLANLLTSLFRTVPIFISDLGLE
VTLSPGWCRVFMLLWVWWRAVGCWVTLALSAFHCSTLRRQHVAFGPLAVQKERRRVWGALGLVWGVNLVLSLPAL
VYTTHVHGNATVELMVISCTTRPLLGCIWEFPSREQGEAFASTSLVLNEVLPLVLMICTNLATLHALAKHIRAVT
AAGGDSGDMDRHLASERKAGHVIMSLVSLFVVCWVLQVAAVTYYNHDGGHHAEGLLTVAHFAASLFVGFSPMVVA
LGHGKLRKRIVGKLVGCSNVVRCRGHEGEDDGGAAGAAQQKEKGKTVFVVQKEAASTKEKTMSKK

>Pf-ORA4

MSEILTVDAILFGLFVFSGILGNILVIHVVFQSALESPSRRLPPSDTILVHLSLANLLTSLFRTVPIFMSDLGLD
VSLSPGWCRIFMLLWVWWRAVGCWVTLTLSIFHCATLRRQHVTFGPLTLQRERRRVWVVLGLVWGANLAFSIPAL
LFTTHIESNATMELMVISCTTRPLLGCVWKFPSDRQGLAFASTSLALNEILPLVVMVFTNLATLHSLAKHIRAVT

SESGSHGELDKHVSTERKAAHVIMCLVSLFVVCWVLQVAAVTYYNHDGGQHAEGLLTVAHFSASLFVGFSPLVVA
LGHGKLRRKIRSMILVWTNVPLSQEAESGRKSPKTSGKQISFVAQKEVKVKKVKDKVIPRR

>Xm-ORA4

MAEVLTVEAILFGLLVFSGILGNILVIHVVFQTAFESPSRRLSPSDTILVHLSLANLLTSLFRTVPIFVSDLGLD
MSLSPVWCRIFMLLWVWWRAVGCWVTLTLSIFHCTTLRRQHVTFGPLTLQRERRRVWVVLGLVWGANLVFSIPAL
LFTTRVESNATVELMVISCTTRPLLGCAWKFPSDQQGSAFASTSLALNEVLPLVVMVFTNLLILHSLVKHIRAVT
SESGSHGELDKQVSTERKAAHVIVSLVSLFVICWVLQVAAVTYYNYDGGRHAEGLLTVAHFSASLFVGFSPLVVA
LGHGKLRRKIRSLILMWTNVPHSQETESGKKSLKTSGKKPSFVAKKEVKVKMVKDKVMPHR

>Dr-ORA4

MSEVLTVDAVLFGLLVFSGIIGNIMVFDCAKLCASRHLPPSDTILVHLCLANLLTSVFRTVPIFVSDLGLQVWLT
AGWCRVFMLLWVWWRAVGCWVTLALSAFHCATLRRQHVSMGPLGHSRERRRVWVVLAVVWAANLLFSLPALVYTT
QVRGNATVELMVISCTTRPLLGCVWEFPTFQQGYAFASSSLALNEVLPLVLMVGTNLATLQALGKHIRTVRAGGS
TGAELDRHVSSERKAGHVIMALVALFVGCWVLQVAAVTYYNHNRGAHAEGLLTVAHFSASLFVGFSPLVVALGHG
KLRRRISGILQSCMHRLKQTQDKPAEITEKDGRTTQSAMK

>Ga-ORA4

MSTRSNLHALRRSLMFSDAQRNCGKCLRLHVFQSAFESPSRRLPPSDTILVHLSLANLLTSLFRTVPIFVSDLGL
DVSLSLGWCRIFMLLWVWWRAVGCWVTLALSIFHCTVLRRQHVACGPLAQERERRHVWIALGLVWGANLAFSIPA
LVYSTHVHGNATVELMVISCTTRPLLGCVWEFPSNQQGSAFASASLALNEVLPLVLMVCTNLATLHALAKHIRAV
AAGAHPGETQKELDKHVSTERKAAHVIVSLVSLFVVCWALQVAAVTYYNHDGGDHAEGLLTVAHFSASLFVGFSP
MVVALGHGKLRRRIMNMILMWSEVLKCRKENSEERIKSPKAKGRRGKRSVFIVQEERMVVQVKGNAQADK

>Hc-ORA4

MSKVLTVEAILFGVLVFSGILGNILVIYVVFQSVTKTPPRRLPPSDLILVHLSLANLLSSLFRTVPIFVSDLGLD
LYLSSGWCRVFMLLWVWWRAVGCWVTLTLSAFQCTTLRRQNVAFGPLTVQRERRLWVVLGVVWGANLAFSVPALV
YSTHVKGNATVELMVISSTTRPLLGCVWEFPSEEQGTVFASTSLAVNEVLPLVLMVCTNVATLHALAKHIRAVAS
GGTHTELDKHLSSERKAAQVIMSLVLLFVVCWVLQVAAVTYYNHNRGHHAEGLLTVSHFSSSLFVGFSPLVVALG
HGKLRRKIMSMMLG

>Ol-ORA4

MSKVLTLDAILFGLLVFSGLLGNTLVIYAVFQSAFETPPGRLSPSDTILVHLSLANLLTSLFRTVPIFVSDLGLD
VSLSPGWCRVFMLLWMWWRAVGCWVTLTLSIFHCTTLKRHHVSLGPLVLQKEKRRVWIILGLVWGANLAFSIPAL
VYSTHVYSNATVDLMVISSTTRPLLGCIWEFPTTQQGSAFAFTSLALNEVFPLVLMICTNVSTLHALAKHIRAVT
SSMESGGSHGEVNKLVSTERKAAHVIMLLVALFVVCWVLQVAAVTYYNHNRGLHAEGLLTVAHFSASTFVGFSPM
VVALGHGKLRKKIMGMILVWTKAFHCSSKDTGRKRPSPKKKKLKVFDVQEEMRVINIRECKALNDRRE

>On-ORA4

MSEVFTVEAILFGLLVFSGILGNILVIYVVFQSVTKTPPRRLPPSDLILVHLSLANLLSSLFRTVPIFVSDLGLD
LSLSSGWCRVFMLLWVWWRAVGCWVTLTLSVFQCTTLRRQNVAFGPITVQRERRRLWVVLGVVWGANLAFSVPAL
VYSTHVKGNATVELMVISSTTRPLLGCVWEFPSEEQGLVFTSTSLAVNEVLPLVLMVCTNVATLHALAKHIRAVA
SGGIHTELDKHLSSERKAARVIISLVLLFVVCWVLQVAAVTYYNHNRGHHVEGLLTVSHFSSSLFVGFSPLVVAL
GHGKLRRKIISMMLG

>Ss-ORA4

MSEVLTVDAILFGFLVFSGILGNILVIHVVFQSAIESLSRRLPPSDTILVNLSLANLLTSLFRTVPIFVSDLGLD
VSLSQGWCRLFMFLWVWWRAVSCWVTLTLSAFHCATLKRQHVAMGPLAQEHERRKVWVALGLVWGLNLAFSLPAL
VYTTHVQGNATVELMVISCTTRPLLGCMWEFPSEEQGSAFASTSLALNEVVPLVLMVGTNLATLHSLAKHIRAVT
SAGEAGGGTHGELDRHVASERKASHVIMLLVMLFVVCWVLQVAAVTYYNHNRGNHAEELLTVAHFSASVFVGFSP
MVVALGHGKLRRRIMRMIAGCADRVKCQQEKIIDESKAPDKRERTAKQTVFTIQKEREVIK

>Tn-ORA4

MSEVLTVDAILFGLLVFSGILGNILVIHVVLQAAFQGTSGRLPLSDTILVHLSLANLLTSLSRTVPIFVSDLGRD
VSLSAGWCRVFMLLWVWWRAVGCWLTLVLSVFHCTTLKRQRLYIGPNAQRRERRRLWVILGLVWGLNLAFSTPAL
IYSTHVHGNATVELMVISCTTRPLLGCIWEFPTAQQGSAFASASLALNEVLPLVLMVCTNLATLHALAKHIRAVV
SSQSGGSQGELDKHLSTERKAAHLIMLLVSLFVTCWVLQVAAVTYYNHDRGHHAEGLLTVAHFSASLFVGFSPLV
VALGHSKLRRRISKVMLSWCRRLKGSSNTKLMQIVFFVPQKQ

>Tr-ORA4

MSEVLTVDAILFGLLVFSGILGNFLVIHVVLQAAFQSASRRLPLSDTILVHLSLANLLTSLFRTVPIFVSDLGSD
VSLSPGWCQVFMLLWVWWRAVGCWLTLALSIFHCTTLKRQRLYMGPDMQRRERRRMWVILGLVWGLNLAFSIPAL
IYSTHVHGNATVELMVISCTTRPLLGCIWEFPTAQQGSAFASASLALNEVLPLVLMVCTNLATLHALAKHIRAVM
SSGQPGGSHVELDKHLSTERKAAQVIMLLVSLFVVCWVLQVAAVTYYNHDGGHHAEGLLTVAHFSASLFVGFSPM
VVALGHSKLRRRISSMMLGWCQCFKGRSEDDPPNTRAAKIISFVQHKQ

>Lo-ORA4

MAQVLPVDAILFGVLVLSGIVGNVLVICAVVQSVLQNSLLRIPPSDLILANLSLANLLTSFFRTVPIFVSDLGLE
VSLAPGWCRLFMFLWVWWRAVGCWATLGLSLFHWAMLRRHSFMSGLQAHRAELRRVCVALALVWALNFAYSLPAL
VYSTHSRGNTTVELMVISCTTRPLLGCVWEFPSEVQGIAFATASLVVNELVPLVLMVGTNLASLCVLRRHIHTVA
GANMELQGHMASERRASHVILVLVTLFVTCWGLQVTAVTHYNYNRGRQAETLLTVSHFAASVFVGFSPLVVALGH
SKLRGRLRRMLRLHCGRREGPGGEDVGDRERDREAATKTPTTTRTCRDKS

>Am-ORA5a

MNAKEWIKSTIRGFMCVSGILGNNWLGFCSLPKSRSQLRTNNILFINLAISNLITNYMVDLPDTLELVKRWPVGR
MYCSAFNFFSDLSETSSIFTTMFITVFWHQKLVGSLKHGGAPVQMDNTRLVMALLAGSWTVSVVFSLPHLFFTSL
NIQNQSSEECLEYFPSQEVKQTYEMVFLMLANVVPIVGIVFASIQITVTLLQSQKRIKNISSRAGPRGDDQRKAA
SNELSSKDYISNATSANAPNTIQKVQKSQDRSNSSSGSSQVRAAKSVVAVATVFVICWLTHLLLSITSTIHDSIV
IHEMTSYIGALYTCIIPYIYLYGVKKLTCLTCSSID

>Am-ORA5b

MDAEGWIKSVIRGLMCVSGIIGNHWLGFSALPKSRAHLKTNDILFVNLASSNLITNYLVDLPDMMDFTYNFLMGQ
MYCSVFNFCSDLSETSSIFTTLFITVFWHQKLVGSLKRGGAPVQMDNIRLVAALLAGSWIVAIAFSLPHIFLASK
NNGNNTYFECLEDYPSLKAKQAYDLMYLVFANIIPIIGIFFASIQITVTLLQNQKRINSNTTAVTTGAKNTKITP
AEPSVHSNQSSQEAVSYSNAVVPNPGQARSSSSSGSLLRAAKSVVTVATIFLICWVVHVILRLISTIQESSLIME
LASYIGAAYTCIIPYIYLHGVKKFSCTCRG

>Fh-ORA5

MDADELIESIIRALMFIAGILGNNWLAITSLPKRRSDIRTNEILLVNLAVSNLITNCLVDAPDTMADFAGRWFLG
ATFCGIFRFSADLSETSSLFTTLFICAFWHQKLVGSLKRGGAPVQLENLRLVGCLLAGSWILSTVFSIPHFFFVS
LEGTNGSREDCIDVFPDALSSQIYEIFFLVLANALPVAGIMVASVQIVVTLLQNQKRIQSRSSDPAKGAIKENHP
ERSVSSSVSVPDHCKDLKGPSSPAHSGKTGHKADSGVEAQANSSRPSQMPSKQSTSAHSQVRAAKSVVAVATVVLI
CWVTHLLLRISNNIQTSSLMMELASYIGASYTCIIPYIFLHGLKKLSCCRKS

>Gm-ORA5

MDVDDIIESAVRALMFLLGMLGNNWLAVRSIPSRLSALRTNELLFLNLAVSNLITNYLVDLPDTMADIAGGWFLG
DGYCGVFRFCADLSETSSIFSTLFISVYWYQKLVGSLKRGGGPVQLDSLRLVGGLLAGSWGVAVVFSIPHYFFVT
VEGENGSSLECNDVFPSEEAKQTYEALYLTLANALPVAGIVYATARIVVTLMQSQKRIQGHGGNQAASEEGRAAP
AAAASIKAAGGAEGGGVAGGGEVRGAKPAPKPSPGSSNQVRAAKSVVAVASIFVVCWVTHLLLRISSNIQTSPIV
VEVASYIAASYTCIIPYIFLYGVKKLSCSCCGAKQ

>Pf-ORA5

MDADKLVESIVRILMFIAGILGNNWLAIASLPRKKSEIRTNEILLINLAVSNLITNYLVDIPVTMEDFAGQWLLG
LTFCGIFRFSADLSETSSLFTTIIICVFWHQKLVGSLKRGGAPVQLDNLRLLGCLLVGSWTLSVVLSMPRLFFVS
LEVTNESLEHCVDVFPNVLSRQTYEVFFLSLANALPIAGIWVASIQIVLALLQHHKRIQSASSHRVNVKQDKSER
SVSSVSVQDSHKDLKESSSPAERVSSCPNGECLGKSPQSSPLANKTDSRAEAQPNHSKSNQIPSKQSNNTSSQVR
AAKSVVAVATVVLLCWMTHLILRISNSIQDSSLMMELASYIGAAYTCIIPYIFLHGVKKLSCSCK

>Xm-ORA5

MDADKLIQSIVRILMFIAGILGNNLLVIASLPRKKSEIRTNEILLINLAVSNLITNYLVDIPVTMEDFAGRWFLG
LSFCGIFHFSSDLSETSSLFTTLIICIFWHQKLVGSLKRGGAPVQLDNLHLLGCLLAGSWTLSAIFSMPRLFFVS
QEVKNESHENCIDVFPDVLSRETYEVIFLSLANALPVAGIWVASIQIVLTLLQHRKRIQSVSSHRVKVKQDKSER
SVSSVSVQDSHKDLKESSSPTEHVSSCPNGKCLGKSPRSSPLANKTDSRAEAQPNHSVCQSSQIPSKQSNNASSQ
VRAAKSVVAVATVVLLCWLTHLILRISNSMQDSPLMMELASYIGAAYTCIIPYIFLHGVKKLSRLCK

>Dr-ORA5

MQLQDWVESSIRAFFCVTGITGNFWLALRSLPRSRSRLRPNDVLFINLAVSNLITNCMVDLPDTLAQFLNSWLLS
RNYCSVLQFSSDLSETSSIFSTMFITLYWHQKLVGSVRRGGAPVQLDNLRLVLWLLLGSWMVALTFSVPHFFIAE
HDGNDTLEVCEEKFPTPAEKKTFDGLYLIVANVVPLVGITYASVQIVVTLIQSQKRVKDHSGGSEQKTEVRSSGN
PSSGTHVRAAKSVVAVASIFIFCWFIHLVLRIYSGFRNSILVVKLTNFIGATYTCFVPYVYLHGVKKLNCSCCW

>Ga-ORA5

MDAEGWIESLIRALMFLAGILGNNWLAIRSLPGHKSSIRTNEVLFINLAVSNLITNYLVDLPDTVADFAGHWFLG
ETFCAAFRFCADLSETSSIYSTFFISVFWHQKLVGSLKRGGAPVQLDRLCLVGCLLAGSWTVAAVFSIPHVFFVA
VEGRNGSKVDCVDVFPSAVARQTYEIFYLTLANALPLAGLVFASAQIVVTLLRNKQRVQGHSSGASEEGENKSGG
GRDGGVAGTASGPGPTEDPKDPSSLTDIYTGVPASARPTGGSPGHAGTLVGDTYSGGGAPEGPGRPSQTRAKTSS
GTQVRAAKSVVAVAAVFLVCWLTHLLLRISNNIHTSSMLVEVASYIAASYTCIIPYIFLYGVKKLGCPCRR

>Hc-ORA5

MEGLIEAIIRALMFIAGILGNNWLAICSLPRHKSSIRTNEVLFINLAISNLITNYLVDLPDTMADFAGRWFLGKT
YCGVFCFCAGLSETSSIFTTFFISVFWHQKLVGSLKRGGAPVQMDSLCLVGWLLAGSWTVAVVFSVPHYFFFTLE
VANDSHEDCIEIFPNPNARQIYEAIYLTLANALPMAGIVFASAQIVITLLQNHQRIRSHNSDQTKEMVKEERKRS
ESKRNKASVSIISGPTTSKDLRDSTSTNHIYTGVPAPSSPNRQPSGHSLHNAPQNCSVGAQPNLSRPSQIPSKPH
PNSSTQVRAAKSVVAVASVVLVCWLTHLLLHITNNIHTSSIVVEVSSYIAASYTCIIPYIFLHGVKKLTCSSKR

>Ol-ORA5

MELNKPIASVISALMFLASILGNNWLAVASLPKDRSAIRTNEVLFINLAVSNLITNYVVNLPETMADIADNWFLG
ETFCCVFLFSIDFSETSSLFSTFLISAFWHQKLVGSLKRGGAPVQLDNLCLVGFLLAGSWTVCAVFSIHHFFLAS
VEGINGSHRYCVDAFPSALAEQTFDIIFLTVANVFPLVGIIVASFQIVVTLLQSQKRIGGHTSVSPKEMIREDKS
SQSKKQVMGPLKGLKVVRTYKSEPVTSSVTLPQTSSQNKTTGSNCSLGAPANHSKPSKANPNSSTQVRAAKSVVA
VGSVVLVCWLTHLLLHITNTVHSSQSTLEVAGYITASYSCIIPYILLHGVKKLTCSHKR

>On-ORA5

MEELIESIIRGLMFIAGILGNNWLAICSLPRHKSSIRTNEVLFINLAISNLITNYLVDLPDTMADFAGRWFLGET
YCGVFCFCAGLSETSSIFTTFFISVFWHQKLVGSLKRGGAPVQMDSLCLVGCLLAGSWTVAVVFSVPHFFFFTLE
GANDSHEDCIEIFPNPNARQTYEAIYLTLANALPMAGIVFASAQIVITLLQNHQRIRSHNSDQTKEMVKEERKRF
ESKRNKASVSIISGPTSSKDLRDSTSTNHIYTGVPAPSSPNRQPSGHSLRNAPQNCSVGAQPNLSRPSQIPSKPH
PNSSTQVRAAKSVVAVASVVLVCWLTHLLLHITNNIHTSSIVVEVSSYIAASYTCIIPYIFLHGVKKLTCSSKR

>Ss-ORA5a

MDATEWIEAFIRGLMCLLGILGNNWLCLRSLPGPKSSLRTNEVLFINLAVSNLITNYLVDLPDTMADFVGHWFLG
EAYCCVVQFCSDLSETSSVFSTLFISVFWYQKLVGSLKRGGAPVQLDSLRLVACLLVGSWTVAVVFSVPQLFFVR
MESGNESHDDCIEIFPSQTARQTYEPLYLTFANALPIAGIAFASIQIVITLLRNQTRIQGLTSDHHKGTANSLPN
NGPSVVHSSISSPLYQVDIGDNIVRAPADLKRSSQPPAKPSPGSGTQVRAAKSVVAVATVFVVCWVTHLLLMMAS
NIHTSSLVLELASYIGSSYTCIIPYIFLYGVKKLSCSCRG

>Ss-ORA5b

MDAKDWIEALIRGLMCLVGILGNNWLGLRSLPGPKSHLRTNELLFINLAVSNLITNYLVDLPDTMADFAGRWFLG
EAYCGVFRFCSDLSETSSIFSTLFISVFWYQKLVGSLKRGGAPVQLDSLRLVAYLLAGSWTVAGVFSVPHFFFVQ
VDSGNESHKDCIEVFPSQTARRTYETLYLTLANALPIAGIVFSSIQIVITLLRNQMRIKGLTSDHHKGTDKALPN
KREKMDVSEKYDETGIKTSDKEADLPIVASPDFPQSCRNQRISDIRPNLPGVSCSVLPCAGPSVVHSSISSPLYQ
VEVGDCRVRASPDLQRTSQPPAKLSPGSGTQVRAAKSVVAVATVFVVCWVTHLLLRISSNIHTSSVVVEVASYIA
SSYTCIIPYIFLYGVKKLSCSCRR

>Tn-ORA5

MDTKELAESIIRGMMFLAGILGNNYLAARSFPTQRTSIRTNEVLFINLAVSNLITNYLVDLPDTMADFAGRWFLG
ETYCGIFRFCADLSETSSIFTTLFISVFWHQKLVGSLRRGGAPVQMDNLCLVACLLAGSWTVAAVFSVPHFFFVK
VEATNESSEDCIDVFPNKLAKQTYEIIYLTLANIFPVAGIVFASMQIVVTLLQNQRRIQSHSSNPTQTTNRTEDR
SSCTSGKDSTLTSPVYTGVPPVSADGGALAQSSGEPQPGGTARSHQECPRPGQAPTKPTLASGSQVRAAKSVVAV
ASVFLVCWLTHLLLRISNNVHTSSVVVEVASYIAASYTCIIPYIFLHGVKKLYCSCKR

>Tr-ORA5

MDAEELVESIVRGLMFLAGVLGNNWLAVRSFPTQRSSVRTNEVLFLNLALSNLITNYLVDLPDTVADFAGRWFLG
ETYCGIFRFCADLSETSSIFTTLFISVFWHQKLVGSLKRGGSPVQMDSLCLVACLLAGSWTVAVVFSIPHFFFVK

VEGSNESSEDCIDVFPNKAAKQTYEIIYLTLANAVPVAGIVFASVQIVITLLRNHRRIRSHGPDPTKISNEPKDR
SSDPGPTPPSQVYTGVPPSGGAVAQSSREPQPGGTDRTPEGGPRPSQAPAKPTMASSSQVRAAKSVVGVASVFLV
CWLTHLLLRITNSVHTSSLVVEVASYIAASYTCIIPYIFLHGVKKLHCSCKR

>Lo-ORA5

MDTVGVIESTVRASMCFLGIMGNSMLVLHSLPSKRSHLKTSEVLFINLAASNLITNCLVDLPDTLADIAGRWFLG
EAYCGIFLFCSDLSETSSVLTTLLISVFWYQKLVGSLKRGNAPVKLDSLGLSCGLLAASWGAALVFSVPLLSFVT
VGSNRSASQDCQAHFPTHASKQTYEATYLTLANAVPVAFMVFTNLQIVITLLTQRKRIEALKKEARLQPAAERAQ
PTSTGPDSPREFSSVSTPAGNQNPSSQALADPAGKGRLDALARAAAVAPPRSRNAPRQHPGAQVRAAMSVVAVAS
VFLVCWVTHLLLRIASNVNESSAIVEIASYIAASYTCIIPFIFLHGVKKLSCRCWK

>Am-ORA6

MESFIFGLLVLRIMLSVIGVLGNTVLIVSILQMTRLKTFEVFLLGLAVSNLEEIMIVDIYDMIVLRSTHSISILS
CGVLKFMTLSGEVASIFFTVLISIYRYQKLHNAAMRIITPIFMDSMKIGVGLSLLCVLVAVLASVPTYIINLDSW
HHMYNSTITDCPADFFQCPRDNCPILNNIYRFLFIFFCYLIPLVIVTGTSSLIIRILMIQQKVAELHHNSEPATI
AANNDHHHHHHNHHHHHDNHHHHHDHHHHDHTNVFHRSTIGILAAMMIFQVYCILYLARHLAFNLYDFPAWSELE
FFIATFYTALIPYVYGMGHNFFSLKHFRRQ

>Fh-ORA6

MCHLSVYLFGLKTFISCIGFVGNIFLIVSIFQTAVSHVKPFELFLLGLASANLEEIVIVNIYDVHVLEVFSATAG
SWRCRLIGFMTVFGEIASILFTVVICIFRYQKLRDINHRGSLPIWLDSITSAGMMSGVCVTLSTLVSLPVFFTLQ
ESVKNTTVNSGGCPSDLFQCGENYCPTLNRVYKYLMMILCHLLPLIIVTVTSCLTITVLLRQIYSVTPANDARCP
DHPSGKSHGLQRTACTQVGRCEHGGVALGLHLPVCVYVRGNLPRLVSAAETPRNSWSFAADVIATACPCGTRRRD
GAAR

>Gm-ORA6

MAKVEYKTMITLVTIRFVMSLIGIMGNMFLVFVIFQTKISRIKSFEVFLLGLAVSNLEELVVVDFYEVIMLIGHI
QNSLLCRTMKFLNLLGEVSSILFTVLICVFRYQKLRDAEKRGNAPIFLDSRKSAWVVSGLCMLLSVMLGLPVYFV
RIDTHVEADNGTSCSPDFFQCHEHFCPPLNRFYKYLFLVSCNLLPLLAVTVSSSLIVKVLLGQKRVVAPALGASG
PPGKKSKGPRLQRSTVGILTAMGVFQIDWTMYLVFHLAFSPVNVPLWGDIEFFITTSYTTLSPYVYGIGYDLFSL
RYFIKR

>Pf-ORA6

MSDLSVDLQGLRMFVSCVGLVGNIFLILSIFQTRVSNIKSFELFLLGLASVNLEEIVIINVYDVIILDTVSTTTG
AWWCRLLKFMTTFGEIASILFTVIICIFRYQKLRDVDHRGSLPICLDSIASAWMLSGVCVTLSTLLSLPMFAVTF
QGSVENVTENRGGCPTDFFQCGENYCPIFNRVYKYLIMLLCHLLPLIIVTVTSCLTIVVLLGRTNTVTPANDIIS
PNHHHGKSHSLFRSTVAVVAAMGLFQVDWTLYLILQWTFSPSDCPIWIEIEFFISTSYMSISPYVYGIGGHLFSL
ENCKHLLKR

>Xm-ORA6

MSHLSVDLLGLRLFVSCVGLVGNIFLILFIFQTRVSHIKSFELFLLGLASFNLEEIVSINVYHVIILDTVFTTTG
AWWCRLLKFMTTFGEIASILFTVVICIFRYQKLRDVDHRGSLPICLDSIASAWTMSGVCVTLSALLSLPMFAIAF
RGSVENVTENREGCPTDFFQCGENYCPILNCVYKYLIMLLCHLLPLIIVTVTSCLTIVVLLGRTNTVTPANDIIS
PDHHPGKSHGFYRSTVAVVAAMGLFQVDWTLYLILQWTFSPSDCPIWVEIEFFISASYMSISPYVYGIGGHLFSL
ENCKHLLKR

>Dr-ORA6

MEQIQVNLLSLRLFISIIGVVGNTLLLVSILHTHTHLKSFELFLLALCSANLQQLVMVDVYDVLLLCSPSCIGVC
SCRALRFLTVFGEVCSVLFTALISIYRHQKLHDVFSHVNVPVLLDSLRWAVCMCVLCVCVALAFGLPTLLVNTHW
SVSNSSLERCPVDFFQCPSSSPCLTHIYKYVFLLVCVVLPLLVVTVTSVLMVRVLLAQQRAVRVREAEEPHPHHH
HSSLLRSTLAILAAMLLFLLDWSVYLLLHLAFDPYSFPLWAEVEFFITTIYTALSPYVYGIGNDLFSIKRLYC

>Ga-ORA6

MVGLSVDLLGLKVFILCVGLMGNVFLMVAVAQTKFPRVKSFELFLLGLAAANLEEIAITTVFDVDFLQASSRGVD
TWSCRSLKFLSKFGEVASIFFTVLISVFRQQKLSDAAKRANLPIYLDSIGSARMASGVCVLLATLLSLPVFAIEP
KEPAGNATGNATGNATGNAIGCPPDFFQCSKSRCPALNGLYKHVFILVCNLLPLAVVTVTGCLILAVLLGQRSTV
TPASVGSRSGGSTLRRSSVAVLAAMGLFQVEWTLYLILQLTAVYVDFAFRAEAELLISFSYTCISPYVYGIGNDL
FSLKNFKRN

>Hc-ORA6

MAELSVNLLGLRLVFSSIGLMGNTVLIASIIKINFFHIKSFEIFLFGLAAANLWEIVIINIYDIIILQTPSTATG
TWSCYLLEFVTVIGEINSIFFTVLICIFRYQKLRDLNTRVNFPLFLDNIRSAWTVSGISVMLSVLLSVPMFVIDQ
ESKAENVTRNSSMCPPDFFHCTQNHCPVFNRIYKYLFIVSCHLLPLIIVTVTSCLILTVLLSQRKTVTPAVNETG
SSQFSRKSKDTKIQWSTIAVLGAMGLFQVDWTTYLIFQLAFNPYEFPFWSEAQFFITISYTSISPYMYMIGHNMI
PPHSCKKG

>Ol-ORA6

MEGIYVNLLGLRIAVSFTGLVGNVCLILSIIHVKWSHIKSFEVFLLGLAAANLEEIVILNVYDAFMLQTSSSDTW
WCRFLKFMTMFGETASIFFTVIISIFRYQKLGVSLSVHPDRIGVAQLLSGVCVMFSFLLSFPVVAIKPAALTESA
ANNSGGGCPADSFHCGKNYCPAPNRAYKYLFILVSYLLPLIVITVTNCLILAVLLVQRRTITPEISVHQSNHSHT
NGRDLRFQHSMMAVVAAMGLFLVNWTFYLLFLFLLKPNNLPSWREIEFFTLTSYSCFSPYVYGIGHNLFSLENFK
IIRNKF

>On-ORA6

MAELSVNLLGLRLVFSSVGLMGNTILIASIIKINFFHIKSFEIFLFGLAAANLWEIVITNIYDIIILQTSSTATG
TWSCYLLEFMTVIGEINSIFFTVLICIFRYQKLRDVNTRVNFPLFLDNIRSAWMVSGISVMLSVLLSVPMFVIDQ
ESKAENVTRNSSVCPPDFFHCTQNHCPVFNSIYKYLFIVLCHLLPLIIVTVTSCLILAVLLSQRKTVTPAVNETG
SSQFSRKSKDTKIQWSTIAVLGAMGLFQVDWTIYLIFQLAFNPYEFLFWSEVQFFITISYTSISPYMYMIGHNMI
PLHSCKKGSFKGTVSDFVEVI

>Ss-ORA6

MVDTLELLVFRIFISTVGIVGYVVLILSLIKNQISCLKTFEVFLLGLAASNLVAPNHNVHPHHLVLPAPQVLHNG
LAFLGQWWAISRLLLVLLVSYTAEGYMSLRPGQAEIKLCVWLYLFGMLVFGKIASILFTVLFSIFRYQKR

>Tn-ORA6

MLALSVQLLATRIIISCIGIFGNVFLIISVVQTKFSRIKSFELFLLELAAANLEEIFIVNVYDIILLCTSYATVG
TWSCRTLKFLTSLGETASILITVLISIFRYQKLRDASRRVPIYLDSIRSAWTVSGILLMFTVLLASPIFVLNIKE
MSQNVTINGSGCPPDFFQCNKDNCPELNGIYKYLFILLFNLLPLIIVTVTSCLIIMVLLSQRKTVAPVVNASSQT
AQRSKCQKFQRSTIAVLTAMGLFQVDWTLNLIFQLTSSPGAFTSGAEIKFFISSSYTAISPYVYGIGNNLFSLKK
FRKT

>Tr-ORA6

MLGLSVQLLATRIIISCIGIFGNVFLIVSVVQNKFSQIKSFELFLLELAAANLEEILIVNIYDMIILQTSFATVG
TWSCRLLKFLTMLGENASILVTVLISIFRYQKLRDASRRVNLPIYLDSIRSVWTVSGILTVFTILLSSPIFVLNI
KETSQNFTNNGSGCPPDFFQCNKEDCPELNGIYKYLFILLFNLLPLIIVTVTSCLIIAVLLSQRKTVTPVESGSS
QISRKSKGLKFQRSTIAVLTAMGLFQVDWTVYLIFQLTISPGDSSSWAEIKFFISTSYTSISPYVYGIGNNLFSL
KKLRKN

>Lo-ORA6

MDLMNPVLLVLRYLISIVGIIGNITLVVSILSHSHMKTFEIFLLGLSFSNLEGIFLVSIFDITTRLALQSLEEWS
FKILRFMASLGETATIFFTVLISVFRYQKLRHAEARGNLPTSWDNTSTAWALSGMSLFLSFCFCLPGYFIESDER
VDNHTRSRNFLTDPFQCPRINCPAINLIYKTLFLLFSNLIPLLIITATSGLILKVLLHRRKTVSDVYDSSHHHHQ
NLYFSKSTKTVLAAMCIFQLDWIMYLVLHLAFDSSKMDNWSEIEFFIVTTYTTISPYVYGIGTNIFSCRQIVKVL
SCLSFSACGLYRQHSMCAKWNQ

>Lo-ORA7

MDLQNLAKAVATLLQNMVGIPANLTVLGVFVHVARTERRLLPTDAIVSHLVSVNLLLILTRGIPQSLSALDYRGF
YDSATCKFLIFTYRTTRAMSISLTFVLSAYQCITIAPASSRLSRLKPWLYRCLLPLNLFFWLLNGGTTYTSILYT
SQVRNTTLSTNTLNLGYCLVVFPSEESYFANGVMYLTRDLFFVILMVLASFYILLLLYRHQRRVKGLQSSNMSQG
SRAETRAAKTVVTLVTLYVLFFGIDNLIWAYTLTTEKVPLLMNDVRVFFSSLYASVCPVVVIVSNRKVNRRLSCI
KLA

>Lo-ORA8a

GLNVAVCQASLLYSSAPTNSSLSEYTLNLEFCIVAFPSFEAYMGNGVMHIVRDFVFVGMMASAGGYIVVILYRHR
KQTRGLQGAARMQRKTVEASKAVLTLIAMYVILFGLDNVVWIYTLCVSRVHPIASDTRVFFASCYSALSPIFIIT
TNKKIVASLSCCKGKDQKHLIAESTVSHLPSGQ

>Lo-ORA8b

VMYIVRDFVFVGMMASAGGYIVVILYRHRRQTRGLQGADRMQRKTVEASKAVLTLIAMYVILFSLDNVMWIYPLC
VSHVHPIVSDIRVFFASCYSALSPVFIITTNKKIAASLSCCKGKDQKHLIAESTVSHLSPGQ

Part 2 African coelacanth V1R protein sequences.

African coelacanth V1R sequences were taken from [6].

>Lc-V1R01

MDLCITIKGVSFLLQTGLGVLANLIVLLAYTHIAYSDHKLVAVDMILFHLAFVNMMSLLTRGIPLTMTIFGLRHI
LNDAGCVLVVFIYRVVRALSVCITCLLSWFQAITIVPATSKLSRFKIKVPNYIIPSFVVLWLINIFIYSGVPYYT
TAPTKNSSVPKYTMSTGFCYVIFPNQMSFSLYGVVITSRDLIFVILMILASGYILLILYRHRQQVKALRNPEYST
KSTAESKAAKIVLTLVLFYIAVFGIENIIGLYVTSLSQVHEYIIDLRVFVSSSYASFSPLIILNFNKKIKSRLRC
AFLE

>Lc-V1R01a

RMDLCTMIKGVSFLLETGLGTLGNFLILLAYAYIVFREHKLSPVDMIFCHLAFANMMVLLTRGVPQTMTVFGLCN
LLNDVECKIVIYTYRIVRALSVCITCLLSMFQAITVAPVTSTWASIKMKAPNNIIPSFLALWLINMAVCIAAPFS
SKAPRNGTVPEYTLNLGFCHVDFKDQVSYVINGIAVTMRDCIFVILMVVASCYILLLLHRHSQKVKSIRSSDRNQ
KTTAETRAAKIVIMLVLLYVVFFGIDNIIWIAMLTIAKVSPVIADMRVFFSSCYAALSPFLIISSNKKIKAVLRC
NSQQREPRDQATDISYVTT

>Lc-V1R07

MDLRAIMKATSFLVLTVIGIPGNFTVLAVFSHIAFTEYKLLPTDIIVTNLALVNFILVISRGFPQILTAFQLRNL
FDTFGCKLIIFAFRIARALSISMTFLLSASQSVTISPATSRLSFLKQRLPKYLWPLIVFFWLLSGATSVTSILYS
TADPNSTASQFTFNLEYCYVAFPGKDAYEGNGTMYVSRDLTFVILMALASIYILFVLYRHSQQVKSIRNPNRNQG
TSAESRAAKTVVTLVALYVIFFGVDNLIWIYSISISRVSPMISDIRVFFSSLYATVSPIVIICSNKKIINKLYCT
RRNQVSQAVEIIFTTV

>Lc-V1R11

SMDAYSFLKGVLFLLLAVIGIPCNFAILGAFGKMIYLGKNLFPVEGIICLLALVNTMMILTRGVPHILFVFEIRR
LYSQHGCAVIIYMARVSRAMAICLTCLLSCSQFLSITPPPSKWISLKAILSKTKNLALIVVCLLFLNLGLCVCSV
LYAMPETNSTNLNFTYNLGYCIVKFPNRHAYLGFGLSLLARDLTFVTSMVIASIAIVMTLLRHRQQVSSIRRSSQ
SHEATVETQAAKSVVTLVTLYVLFFGIENTIFLYTMTGHQVNSVLSDVRFFFSTCYASVFPIVAIVASSKIREQL
KCFTQSKE

>Lc-V1R12

MNLYNLMKGLIFLLMGIIAIFGNLIIMVLFIHIVYVEGKLMPTEFILLNLAWTNMQMVASRGIPQSLYVFGLKKL
FNDSGCRAIIYSARISRAMVICLTCHLSCFQCVTIATSNPKWILVKTKMQKYLVHTIFSLWVFNMFICISRILFT
FSPFNSTSPENTFNLGYCVVPFPDKVSFEVNGYILFTRDIVFVALMALASACILFLLHRHKRQMQSVRRSGRNQE
SNAENQAAKIVVTLVSLYVFFFGIETTISLYQTTIPASVVSIVSDVRYFFSICYCTIFPFILCRFNQKIKNKLKS
SVGETEKKQIKAFFCN

>Lc-V1R13

MELYNLMKGIIFFIVAIIGTVGNLVVLALFLHIAYQHKLTAAERLLLNLAWSNSIMLLTRGVPHSLFVFGLRDLF
SDIGCKIVVYLSRVSRAMSICLTCFLSCLQCITIATSTLKWVYYIKVKMQKYVIPITVFVCLLNMIICIGSVIFS
ISGTNSNYTQFAFNLGYCIVNFPDKLTLHFNGFAPFARDIVFVVAMALASAYILLILYRHGKKVKGIRNSEHSHE
STAEGQATKTVVTLVTLYIIFFGIDNTIFVYQIAVSKEVHTIVSDIRFFLSICYGLVFPFIIIGFNSKIRTKLKP
SSSEQQTEVQEVSSI

>Lc-V1R14

MDLYNLVKGIIFILTAIVGVSGNLVILVSFYHIALQERKFVTAQVILLNLAWANMIMALTRGVPHSLFIFGLRFL
FDDIGCKIVVFASRVSRAMSICLTCLLSCFQCITITKSTLKWLSLKGRMQKYVILIIIGLCVMNMLVCIAAVLFS
VSSTNTTNLEYTFNLGYCLVTFPDQLSFQVNGFAIFARDIIFVVLMALASAYILLVLFRHGRQVKGIRSSDRNNV
TTAEGQATKTVVTLVTLYVLFFGIDNTIWFYQITVSKEVHSIVSDIRFFFSVCYASICPIVIIMFNPQIRNKLKA
SSSEQETQVQEISSTQ

>Lc-V1R15

RVIFILITLIGTAGNLVILVSLTHIAYQEHKLLAIEKIVFNLSGANLVISLVRGIPHNLFLFGFRNLYSDVGCKI

TGYVHITFRAVAIVLTCLLSCFQCATIARGRSQWTFVKLNLQKHLEWIIFGLYLFCMISTIDVIPFSISGQNVTN
LKFAVSLGYCFVVFPDNIMFQLVGYGIFARDFLFVFLMTLASSYILLILYKHRKQVQGIRSTERNHETTAEGQAA
KTVVTLVSFYVSVFGLDNTIWFYEIVSKIMFPVAFDIRNLFSMCYASFFPIVIITFNKKIRHQLKCPRGEQKPQI
ACLSS

>Lc-V1R16

VMDLYEILRRVIFILITLIGTAGNLVILVSLTHIAYQEHKLLAIEKIVFNLSGANLVISLVRGIPHNLFLFGFRN
LYSDVGCKITGYVHITFRAVAIVLTCLLSCLQCATIARGGPQWTFIKLSLQKHLEWIIFGLYLFCMITTIDVIPL
SISGQNVTNFKFAVSLGYCFVVFPDNIMFQLVGYVIFTRDFLFMILMTLASSYILLVLYRHGKQVQGIRSTERNH
EPTAEGQAAKTVVTLVSFYVSVFGLDTTIWFYEIVSKRMFPVAFDIRNLFSMCYASFFPIVIITFNKKIRHQLKC
PRGEQK

>Lc-V1R17

KMDPEIVNGAVHFVISIIGIAGNIVILASFSHITYHKRKIMAVEKILVNLSGANLIILVSRGLPLSLYAFGLRNL
FNDFCCQFIGYVHITFRALSVALTCLLSCFQSIMLAKNSPKTAKLKLKLQTHIVPILSLLCIFSMLCSVDVIVFS
VSSYNVTVPQYTAAMGYCLVVFPNIVTFHLVGYGIFTRDFLFVFLMALASSYILLVLYRHGKQVQGIRSMERNHE
STAEGQAAKTVVTLVSFYVTLFGIDNTIWFYQIISKGLISKVFDIRTLISILYAVVFPFVIIAFNKKIRSKLKCF
TSEQKSEISIIEEMKHIQ

>Lc-V1R18

SMDPETVRGAGYFVITIIGIIGNVVILTSFSHIAYQERKIMAVEKILVNLSGANLIILVTRGLPLSLYAFGLRNL
FNDLCCKIISFVHISFRALSVALTCLLSCFQSIMLAKNSPKTAKLKVKLQAHIVPILSLLFIFCILCSVDMIVFC
ISSYNVTVLEYTDTMGYCLEVYPNAVMFHLVGYGIFARDFLFVFLMALASSYILLVLYRHGKQVQGIRSAEQNRE
STAEGQAAKMVVTLV

TFYATLFGIDNIVWFYQTISEHMISQEFDTRTLISLLYAAVFPLVIIVFNKKIQNKIKYF

VSLQKSELSLTE

>Lc-V1R19

MVSVTGRGAVYFVITIIGIIGNVVTLTSFSHIAYQERKIMAVEKILVNLSGANLILLMTEGLPMSMYSFGLRHCF
NDACHEFIYYVKMTFRGLSVVLTCLLSCFQSIMLAPNGPKTAKLKMKLQVHIVPILSLLFIFCILFNVDMSVFCI
SSYNITVLEYTDTTEYCFAVMFDLMGYGVFARDSLFVFLMALASFYILLVLYRHGKQVQGIRSAEQNRKSTAEGQ
AAKMVVTLVSFYVTLFGIDNIVWFYKTFAYQLISQEFDIRMLTSLLYVTLFPFVIVVFNKKIQNKIKYFVSLQKS
ELSLTE

>Lc-V1R08

MEVRLIFKAAGSIFLEALGIPGNALILATFVFIGISNRKLLPADILLMKLAFVNLIVMLTFNIPTTVSAFGVRKL
FNDDGCKTVIFLFRVGRAMSICITSLLSSYQCIVLAPSFNACIILKQKCPQSLFHITAFLWCLNTIIYAVCVPSM
AEQSFAKSNYSIPMAYCVVSFPSYTFFAVLGMVFIVRDLLFIAIMVVSSSCIVFILYKHMKQVKGIRSSAKNHGR
TAETQAAKAVVMLVILYVFLFGLDNIIWAYSLNLSVLVPEITDVRHFLASCFPSISPIIIIITTNKKLQNKLRFIS
QRKKLQNAETVVSNVHTISE

>Lc-V1R09

MDIQVIFKVISFLLLVVIGIPGNITVMAAFVHLRLSDSKLMPPDIILTKLAFVNLLVVFTRGVPQVLTALGIKKL
FNNNGCRAIIFLFRVSRALSICMTALLSCYQSIVLAPSSKRWRILKQKMPQKLLLIVIIFWCVNMLIYSWTLIFS
YGPLNSTTEYTLNLEFCFVVFPTFAFYIGNGTLYLFRDFLFVGLMVLASGYIVFILYQHRKQVKGIRSSDRGQEN
TAETRAAKAVVMLVALYVILFGLDNIIWIYTLQVSKVATVVSDARVFFASCYSALSPILIIATNKKIQMKLKSLG
QNQHHQTPETSVSHVQVEI

>Lc-V1R10

MNIRVTLKAIGFFLMVVIGIPGNFTILAVFALIKLSNGKLLSTDIILTKLAFVNLLIVLVKGIPQAFTAIGIRKL
FNDNGCRAVLLLYRVSRALSICMTALLSCYQSIIIAPSSNRWRALKQKMPQKLVFIMIILWCVNIFIYSWTLSFS
FAQLNSTTTEYTLNLEFCFVVFPSFQFYIGNGTLYLFRDFLFVGLMVLASGYIVFILYQHRKQVKGIRSSDRGQE
TRAETRAAKAVVMLVALYVILFGLDNIIWIYTLQLSKVAPEISDARVFFASCYSAISPILIIATNKKIQMKLKYL
GQKHHHQTPETTISHVQMEM

>Lc-V1R05

MVLSASISEWITYMVFTFIGIVGNSILIHGILTCPTGRLRPSFLLLFSLAAVHMARNVVVNLLSIIYSAGGVSVF

GSAGCKVFKFASALTGTLGIWFTLYTVVFYSVKLEQAVHPLNCAVNTNWRGYHLAGIFVLWVAGLVVCCPIAVFA
EKAKVQVVGNVTHPYRSSVYVGCRCNYPAPEVALIYGTLALTAIDLVPLVVLAIFSIRIMLLLRKRTGVQFGDIW
IGERTETDVFRAAKCALLLVLLVTALWVSHFTILQCLRRLDEYYFIPTVLAVLSSGYATLSPYLLMIINYRIRAN
L

>Lc-V1R02

TMGTWSIIKGTAHFILTVIGMPGNLIIVYAFSYAAYSDHKLMPADIIVLNLALVNLMVVLVRCIPEMLAAYGINE
LFSDSGCKIVICIYRTTRALSIWLTFLLSGFQCISIAPITTKWAAFKLQAPGHLFGVLAFLWVFNVGFSIPALLY
GISSSGNSTGNGFSINLEYCFVKFPSQYVKITVGNLQISRDIIPIFLMIFASVYILLILYHHSQQVKHLQSSNRK
RGSSAEIRAAKAVITLVLLYVIFFGIDNVLWVYTLTIKAMTTSVISDLRVFFSSLYAAVSPIVIIVSNKKVQKRL
KCEREQQGSLAPETVTSTVRM

>Lc-V1R03

MRFSADNVFYGILVLLGIVGNLLVVVTITVAGYEVGTILASDFILANLAIVNFLISTIRNVPLFISDLGLKIYLS
RDYCKIFMFLWVWLRSVSIWATFCISFFHFLVIRRHHFSVLRKGKELRNIIITTSVIWIGNFFYAFPTCFYSTRA
YGNETDTIQLLSATTRPFLGCIWKFPSLYSGVAYATASLVIHEVFPIFLMVVINLGTLYILYRHSRAVGVETLVT
RVASERRAAKVILILVTLFVICWVTNVLMVNYNHNTEKSIRVFVMLANFGASLFIGFSPVVLMVGHSKLRKKLMN
FTLYLNGIKQKRGKRLTTGKC

>Lc-V1R04

MSQGEHPVQLFFYTMLVALGIVGNSLVIWIVLDTARAANTIPSSDFILLNIAVVNLLISLTRNTLLLALDIGYTF
SMSDGACRILMCIWVWFRCVGVWVTLCLSFFHFMVIRSSHSALGKINERRNVIVIVAVLWALNLLYSSTALALTS
NTSTNISSLVVISSTIRPLLGCVWIFSTDTAALFYGILSFIVHEIIPIFLMVITNCGTLFLLYKHHRQVHRADIA
ITRVESEWKAAKTILALILLFVFCWGTHIVSVNYYNFYSSSSTRYMLIIARFSASGFLGFYPLVVTFGHSKLKRK
FHSAVLFWRKQNEVS

>Lc-V1R06

MELTRIIFLVIRILVCLFGMTGNVAILSVLIKRAFTFRLKTFEMLLVGLTASNFTQELLVDVPEIMKELSGVTIH
RWFCKTLKFTFTFGRANSIIFTILICIFRYQKLRHAVSRVNLPVPLDNVKIIHVISASVLAFTFLFSVPVLLHET
NVEMTLKNQTSCPALFFDCPKINCEVSYMAYKLIYLACIDILPILIIILVITTHLLRILYKNYKLVSVTLDGFSPN
KSKIKDSKVRFWKSTKAVLAALLLFQISWTMQLIIEFAVSSKKFDYWSETDFLIVALYTSLSPYVFGIGNNILT

Part 3 Elephant shark ORA protein sequences.

Elephant shark sequences were taken from [7] and [8].

>Cm-ORA1

MELCTVVKGTSFLLQTAIGILGNLTILLSYAHIGYSDRKLLPVDVILTHLAFVNLMVVLTRGVPQTLVTFDVANL
LNDAGCKIVIYAYRVVRALSVCETCLLSVFQGLTLMPVTSKLANYKRKAGRSIGPLAAALWALNIALCIAAPLFS
VAVANSTVSNFTLDLGFCLVGFPDRVSYVVNGVFISTRDFLFVGLMTFASGYILLILHRHRRQVRAIQRAGSAQQ
AKAENRAAKSVISLVSLYVVFFGIDNLIWIYMLTVPLVPPDIADLRVFFSSCYASFSPLLIISSTKKIKSKLFRA
TPQIGPRPPSTEMSHVQQ

>Cm-ORA2

MEGRSAIKGTLYLLLTVLGIPGNLTIISSFVHIAQHDRRLHPADKIVGNLALVNLAVVLVRCVPETTAAFGLEQL
FDDNGCKIVIFIYRTTRSLSIWLTFLLSGFQGITLAPSTPKWSIAKRLAPRCLPGALALLWLLNMCLSTSAILFS
ISSGNNSTRKKFAVNLEYCLVKFPSRAVKIAIGALQTSRDVIPIALMILASAYILLILYKHHQQVKGLRSSSKGQ
RSSAETRAAKAVLTLVTLYVLFFGIDNIMWVYTLTVAETMQTSVTSDIRVFFSSLYASVSPIVIIASNKKIQNKM
KCTKAKPEMEASETVPSHN

>Cm-ORA3

MTYQFNPHIIFYGALIFLGTLGNFLVLATILAIVLEDHSFPSSDILLTNLTIVNLLISIFWNIPEFILESGVEML
LPLSWCRLFMFIWVWLRCVSIWVTFLLSLFYFLKIKQHVRLSTKAREATFVSATLLAIWLVNFAYAIPALFYAKA
TSGNETVSLMIVSTTVKPFLGCVWNFPNAQAALAVSKSYLVIQEILPMLLMLGTNLSTVYYLQKHMRSIGVERLH
SLQVERKAAKVIMALVTLFVLCWGTHLLAVNYYSYNRGSATSFILTLANYSASLFIGFSPLILAMGHGKLRGKIC
KILRIKLT

>Cm-ORA4

MAEHPVLVVAYGLLVSCGILGNSAVISVTMDSTCERNRLSSSDLILINIAVANLLLSLTRNTLLLTLDAGLPVSF
SSEGCRLMMFVWTWLRSTSIWVTLSLSWFHFVTVRTSRGALERLSERRRVIALMAAEWGVNLVYSGFALGYSSNS
TNSTNHLAVISSTIRPLLGCVWTFPTKQSGFIYALVSVVVHEAIPVVLMLYANAGTLLFLYQHHRKVQDSQLAGG
DRISHEWRAAKTILYLIGLFVFCWGTHVLSVNYYNFKGSPSTHYLLVIARFSASGFIGLYPLVVAMGHSKLKHRL
KNILKCLCP

Part 4 Teleost and African coelacanth T2R protein sequences.

Teleost T2R sequences were taken from [9] and [10], African coelacanth sequences were from [6].

>Dr-T2R5

MLSTQDKLVAWLLIGVLAILTIFFNMYLLLVNYRSYRKKHKLNPADFIITAIAIASISQQVLTYSWQTMDVIDTV
CQISLVEAILLVLVFSVKLIIFWSTAFLTFYYGTKLVVEPVHCYTRIQEAIVKHVHIVLTVIVVSGFANCVPLLS
VLTYYNGTTELGDCGSIMPSDTPGLVYVFYYVVISDIIPGIVMFKCSISISYHLAKHLLDMKASSNGTHGPKLGT
QMRVIKMTLSLVFVYSCFVVVDIYTQTTVVLMRQNTLALIMLFASIYTTVSAFVLIYGKKSYWKELIASYNLFLD
EYPCLNKMKVEEVKHEPHEHSHGH

>Dr-T2R4

MEPWLYALISSPLCLIGMVFNLLFFFCLMRPVSGVTLRNPLRFLLIVVLVNSTFQYLVIAVTIIMLLFDYIFWLE
TVTRALIYQFFCGNFLCNAWISIFYYISIVPQHHAIFIWIKRNIKAILYGGFILNQIVLTFAISTGAVTYFFLGP
VPVNFTALELNSTALAQTLEADMFLFHVANFSYLLYCTCPLVTLIVSWGKTFFYLRGHMKKMGQSGESFSQPQQK
SQMRVTVTGMVQAALFLPSSLWTVAAALLYITGLFEEVDPSRFITMTFCSLSSLGNLLCFGFSQSVFRRGIVSVI
KKLKG

>Dr-T2R2

MSYQCRTLKRKMSTDVGDVLFFLGVGVVGVSGNIFNLIFTVQQQVKTRTIQTVGLILDVISISNIILALAILSMV
VGIFLNPQIWCIKPYPFDLRLEIYLMLTCGFISFWAIAWLSLFYCIKVVNFSSEIFRTLKKNISTVINTAVLLSC
LFSCLFFIPLFSLDTVDSTEQNDNAYGNVTCPMPSFTIQMNQDAYSAAVLFLLCPIPLMIMLPTSVRMVVHLCAH
TRALQKNQTQVQGSDSYLLVCKLTISLVGVYLFNLFFVSLFILMKLIGAYITYQYLVSTFTFYCGVTSALLTASN
RYLKDKLWSLFCCRKAKEPASKSHTVVTGDV

>Dr-T2R3

MGFFFVYISFLAYALVNVPVSIITILMNVFFVYCMFSSEKGQANSVKPPLNVLLWSLIGCSLLHNIFNLLFVLYE
LVYPPVWLYIISGATILFAMRTSFTACLGLQICYFLQIVPVRWPCFIWMKKHIKLFMYVLLFLDRLYFLSQYVIR
VFLEIRRVVMSFNSSSVYDNTTSQSADFGYYMFIADFWLKCCYFFICLGIMLTSGITTVVYLWKHMKRMKENTSS
LSALCKRQQMRVTIMGIIQTVLFFFASGWLMTEEFIECYFGGYDVGTHLASTVMALYSLGTTLLLGIGQSKFRLL
AKDICKKTRKPKS

>Dr-T2R1

MQSNFAYEDHSPFLHKITCILPDVIQYQRRTLKRKMSTDVGNVLFFVGVGVVGVSGNIFNLIFSLQQQVKTRSIQ
TVGLILDVISISNIILVLSTLAMVVSVFLNAHIWCIKPYPLGLRFEMYLMMTCGFISFWAIAWLSLFYCIKVVNF
SSEIFRTLKKNISTVINTAVTLSCLFSFLLFLPAFSLDLPDSADKNISETNITTCPQPTFTLQIDINAYAAAVLL
LICPIPLMIMLPTSVRMVVHLCAHTRALQKNQTQVQGSDSYLLVCKLTISLVGVYLSTLFMVALYFIIKVLGAFM
TYQALVSAFTFYCGMTSVLLTASNRYLKDKLWSLFCCRKAKEPVSKSQTVVTQDV

>Ga-T2R1

MDFQTYAALNGSLAVLNIVTIAFYIFCLVRPLHGEKIKQPLKLLLWTLIGCTKTFLLSGFVSGVTDGFSFQSAVS
SKINQIFYLLMISSVSTSMTSSVWLNFFYYSQIVPAHSALFIWIKNNVKSIIYGFWITERIYCLFDFTSMFLRFT
DFDLLLIRNNFTMVDDMPENNFYKEMFWIVFYTLRAHFVFCLCVMVMSSGSTVLYLCGHMRHMAANGQPASSPRF
RNQVRVTVTGLLQGVLYVFSASWIIYSFFPKNDLYIDFTMIDSTVIMSYMSATLFNLGAGQAVFRQRAEHIWLRA
AHCFKAPQVQQTDQGA

>Ga-T2R2

MDFQTYAALNGSLAVLNTMIIAFYIFCMIRPLHGEKIKQPLKLLLWTLIGCTITYLLSGVVAFFSQISAVSSKIN
QIFDLLMICSVSTSMTSSVWLNFFYNSQIVPAHSALFIWIKNNVKSIIYGFWITERIYSLLDFTSMFLLFTNIDV
SLISNNLTMVDDMSENNFYEEMFWIVFFTLKAHFVFCLCVMVMSSGSTVLYLCGHMRHMAANGQPASSPRFRNQV
RVTVTGLLQGVLYVFSASWTIHSTFQKDGIIYIGFTMIDSTVINLYMSATLFNLGAGQAVFRQRAEHIWLRAAQC
FKAPQVQQTDQGA

>Ga-T2R3ie5

MYVMNKSTIWVLTGLLAVTTVFFNVYIFLMSLLSYRQNKKWSPCETIILALSLADVAHQLVCYLWMTMDEVDSKC
RIADLPYAVMLLLIFSLKFTIMWDTGFLTFYYSTKLVNAPNHCYTRIQAVILKHVSLAVLLIPLCALGTCMPMLV
VFQSTNVTDGNKDCGLLMPASTAGMVYEVIYLLLADVLPGVLMVKCCISISVHLALHLRHMKATTNGTHGPKLGS
QMRVIRMALSLVAVFVLFLVIDLYVNYQITMNHESILTLTFLFTSVYTTVTAMVLIYGKKTFWKALIHEVNVCLD
EYPCLSCLKVPEHKAQTGTTAKD

>Tn-T2R2

MNVDTAELIRVNLPIVILNALSNLFFVFCLMRPSQGEMLKQPLKLLLWTMVCSNLSFLVMLLVRFHFVNDSIVTS
LIGFATFIFSLTMSLNASVWLNFFYYMQIVPSKSALFVWIKRNLKPIIYYICVAEKVQIWLLIGSIIVYNITEKN
YKLEYLNIVNTSYVASFATSSHLRTFTLTMIFSDGVYYIITLCVMMASSLCTVAYLSRHMHQMASQGLSGSRFRS
QVRVTASGVLQGALYVAIFVLTLPQFQFNILDGATAYMTLSDITMINVYMMGTSVNLGMGQIVFRQRAVDLLHRA
VRSCTTIKAPQPQPGGS

>Tn-T2R3

MDIFLIIRINVPMVILNLLSNLFFVFCLMRPSQGETLKQPLKLLLWTIICSTLSFLVTLLVLFFMDHDIPEANLA
ICGMFLLSLSISLNASVWLNFFYYMQIVPSKSAIFVWIKRNLKLIIYYIWVAEKVINGLIVWSILTFSISITNYL
NDFKFPNSTNASYVIFMPSHLVNMRSSIIATQIYILICLCIMLASSWCTVAYLSRHMHQMASQGLSGSHFRRQVR
VTATGVMQGVLYMMVTMCIVSVNLLSDVLSTQASYFSLSNITMINVYMMGTSVNLGMGQTVFRQRAVDLWHRAVR
SCTTIKAPQPQPGGS

>Tn-T2R4

MSVDTAELIRVNLPIVILNALSNLYFIFCLMRPSQGEMLKQPLKLLLWTMVCSTLSFLVTLLVLFHLFSDSIVTS
FTSYGMFVITLSISMNTSVWLNFFYYMQIVPSKSAVFVWIKRNLKPIIYYIWVAEKIYIGLFVSSIVLFNITVIK
YELEYLNIVNTSSVAPFETASHLRTLTTTIVLSGEIYFIFNLCIMSASSWCTVVYLSRHMRQMASQGLSGSRFRS
QVRVTATGILQGVLYLVISVWTLSQFQFSDIIADYAACVSLSNITMINIYMMGTSVNLGVGQTVFRQKAVDLWHR
AVRSCTTIKAQPK

>Tn-T2R5

MNVDTAELIRVNLPIVILNALSNLFFVFCLMRPSQGETLKQPLKLLLWTMVCSNLSFLVMLLVRFHFVNDSIVTS
LIGFATFIFSLSISLNASVWLNFFYYMQIVPSKSAVFVWIKRNLKPILYCIWVAEKVQIWLLIGSIIVYNIIEKK
YELEYLNIVNTSYVASFATSSHLRTFTLTMIFSDGIYYIITLCVMSASSLCTVVYLSRHMHQMASQGLSGSRFRS
QVRVTASGVLQGALFVAIFVLTLPQFEFNVLDGATAYMTLSDITMISVYMMGTSVNLGMGQIVFRQRAVDLWHRA
VRSCTTIKAPQPQPGGS

>Tn-T2R6

MERCYIDLDKRTFQLIMYPLCGINLFSNFFFGYCLVSNRRRLRQPLKMLLTFLVLCTIAFVVHLIISHPLFAEMT
NNDVRHHLSWMITLLIMHSSMTGAVSMSFYYYVQIVPSQRALLIWIKRNIKSFIYVIFLFGEIFLAFSSFVNVLS
VVLDSWVVSANNCTNNELPVVGPTATDTVVSIFVRIHILCCMAIMGVCNFSMTHYLLRHIKSRTRQGFAASETQM
RVAISDLIQAVFFLICGLLYSVCTFIFEYSKQFSFGPLLYLTGVLLYMTGTTASLATGQAIFRQGAVDLWKVLTA
SF

>Lc-T2R01

MLPSVQFSLWIINSFIIVASIFGNLFVFLMNYRSYRRKGYFLPCELICCALSAISGFLEVVFYLWMSMSELDRSC
LISQACYISLLLVIFSLHSALLWITAFLLFFYSAKIVIEPIHCYTKVQDAILKHAPTVLAAIFFFSFTFNIPLII
LSKHTANETNTQQCGNLVVRDKIIFLSIYLPITAILPAVVMVKSSISILVHLMIHLRHLKANTNGFHTPKLSSQM
RVVRMTLVLTIVYLIALITYVLCMTFAMINNNRLFEIAGTAASIYTMASSLILSYGKQSHWNELAKLWREFVALF
PMVCSHRPFGCRRE

>Lc-T2R02

MELSVRVSFLVAFAVAYLLALCMNLFILVRYFHAVRKGEVLQPSDLLLIGLIVCNVVHQTSILALSVLGLFHVGC
YAGGYMFKTLSLVFTSASSAQFWLMAWLCTFYCLNIVRISRRFFIRMRQCVSGLVPHLLAGSVIGTFAVSLPYFV
YVPVVSNNGSLANSTICLMDFRSPAPFYLSLYLTLNCLLPLVLMVASSSVIIMFLHCHVRRMEENTSGFSSPRSD
AYLRITKMLLSLVCLYISFNVATILMNLIKCNFCGLIISSFAEVYPSLCAII IILGTSKLRQGSTPHCPRCLCL

>Lc-T2R03

MNTAYNIGILVIATALTAIGAPGNLFILAINLSTVRSQQVLPPTDLIISGLALSGIVFQTFLVYLVYMELLGMTC
QMEISTFMMIFYITDALGSVNFWFISWLCIFYCAKIVRSGSRLVIRFQQWINGAVRHLLAWSAVGSFTVPVCYYV
FSLGMNHTDILNNCTNLNIAEHKQIVDDSYYCLYLVLCCLLPLVIMTTSSSFILLFLRRHAMRMQRSAADGFSSP

SSEAHVRVSKMVLSLMGIYTVFDISIMIFIFTSNEILVLITILLCYLCPGVTPVI IIWGTAKLRNRLPTACWSK

>Lc-T2R04

MYFDFLIAFCVSIFVLLVIGVSGNLFILVSNLNTVRSQQGLPPSDFILTGLSTCNIIFQISNGTIGVLLLLDVIY
TMQNYAWRLYFYITELLVSIGFWFTAWLCVFYCAKIIRSSSRLVIQIQQWIVGAVPHLLVWSAVGNFVVCTPKLV
LIFLRNETKTSCNSSFGHFRESKWKLDYMNTFYLALCCLSPLVIMITSSSFIILFLRRHVAKIRRNTTNGFSSPS
SEAYVRVSKMVLSLMGIYSTFDVCFMISLFTTNEVLFLIIIFLCHVYSSVTAALIILGTTKLRKRFNTVCWSKCC
NLLKIGI

Part 5 Tree file in Newick format for sequences shown in Figure 1.

(((((Hc_ORA5/1-292:0.025891,On_ORA5/1-292:0.000001)0.999850:0.115729,
((Tr_ORA5/1-294:0.071344,Tn_ORA5/1-294:0.068860)0.999850:0.064032,
(((((((Lc_V1R05/1-292:1.555522,((((((LC_T2R04/1-291:0.319128,
LC_T2R03/1-293:0.422544)0.999850:0.301036,LC_T2R02/1-293:0.699631)
0.999850:0.352664,(Dr_T2R1/34-329:0.100066,Dr_T2R2/10-305:0.102929)
0.999850:1.516280)0.993727:0.178072,((Dr_T2R3/6-286:0.941741,
(((Tn_T2R3/1-287:0.199313,(Tn_T2R4/1-288:0.090901,(Tn_T2R5/1-289:0.022745,
Tn_T2R2/1-289:0.026171)0.999850:0.139829)0.999850:0.133825)0.999850:0.423064,
(Ga_T2R2/1-284:0.043038,Ga_T2R1/1-288:0.060334)0.999850:0.603660)
0.999850:0.220361,Tn_T2R6/6-285:1.045838)0.806714:0.066130)0.999850:0.213120,
Dr_T2R4/1-282:0.982660)0.999850:0.818057)0.744109:0.141197,
((Dr_T2R5/3-302:0.408945,Ga_T2R3ie5/2-302:0.436234)0.999850:0.389266,
LC_T2R01/2-297:0.492591)0.999850:0.812569)0.999850:0.413533,
(((((((Ss_ORA2/1-308:0.110057,(((Tr_ORA2/1-304:0.073026,Tn_ORA2/1-306:0.076923)
0.999850:0.129912,Ga_ORA2/1-306:0.146906)0.987816:0.022595,
((Ol_ORA2/1-304:0.193657,((Xm_ORA2/1-309:0.061025,Pf_ORA2/15-323:0.013873)
0.999850:0.041252,Fh_ORA2/24-332:0.092193)0.999850:0.093860)0.999850:0.114349,
(Hc_ORA2/1-309:0.038427,On_ORA2/1-309:0.008698)0.999850:0.098331)
0.999850:0.044006)0.999850:0.062894)0.991100:0.056815,Gm_ORA2/1-309:0.142100)
0.974001:0.061344,(Dr_ORA2/1-298:0.332525,Am_ORA2/1-298:0.321737)
0.986950:0.064237)0.999850:0.219183,Lo_ORA2/1-309:0.172730)0.999850:0.254048,
(Cm_ORA2/1-309:0.210308,Lc_V1R02/2-310:0.300987)0.339823:0.034496)
0.445654:0.028566,((((Cm_ORA1/1-309:0.311689,(Lc_V1R01/1-304:0.405854,
(Lc_V1R01a/2-310:0.169730,(Lo_ORA1/1-309:0.124883,(((Gm_ORA1/1-303:0.153795,
(Ga_ORA1/1-306:0.056684,(((Hc_ORA1/1-306:0.059797,On_ORA1/1-306:0.000001)

0.999850:0.044944,(Ol_ORA1/1-306:0.073734,((Xm_ORA1/1-306:0.019084,
Pf_ORA1/1-306:0.009971)0.999850:0.028938,Fh_ORA1/1-306:0.030678)
0.999850:0.024495)0.970784:0.015372)0.999850:0.021912,(Sr_ORA1/1-164:0.006235,
Sc_ORA1/1-165:0.007107)0.999850:0.041703)0.889947:0.005132)0.999850:0.034255)
0.992719:0.021645,Ss_ORA1/2-308:0.146575)0.999850:0.042882,
(Dr_ORA1/1-306:0.142605,Am_ORA1/1-306:0.104147)0.999850:0.044504)
0.999850:0.094573)0.999850:0.120938)0.993097:0.052023)0.996604:0.062314)
0.999850:0.174872,(Lc_V1R11/2-305:0.578101,(Lc_V1R12/1-308:0.434657,
((Lc_V1R14/1-308:0.147782,Lc_V1R13/1-308:0.250696)0.994874:0.036637,
((Lc_V1R17/2-309:0.071693,(Lc_V1R18/2-307:0.029732,Lc_V1R19/1-301:0.187340)
0.999850:0.117684)0.999850:0.219360,(Lc_V1R16/2-305:0.039215,
Lc_V1R15/1-299:0.021480)0.999850:0.257617)0.999850:0.279661)0.996854:0.059526)
0.999850:0.115290)0.999850:0.159897)0.999850:0.065054,((Lc_V1R08/1-305:0.492536,
(Lc_V1R10/1-309:0.120550,Lc_V1R09/1-308:0.091326)0.999850:0.138687)
0.859806:0.060401,(Lo_ORA8b/1-125:0.045014,Lo_ORA8a/1-171:0.041007)
0.999850:0.385357)0.999850:0.151643)0.999850:0.056258,(Lc_V1R07/1-309:0.246345,
Lo_ORA7/1-303:0.442535)0.999850:0.127521)0.999850:0.182962)0.999850:0.820478,
(((Lc_V1R04/2-308:0.356525,Cm_ORA4/1-307:0.354635)0.999850:0.326638,
(Lo_ORA3/13-325:0.279786,(((Dr_ORA3b/13-328:0.124232,Dr_ORA3a/13-327:0.132052)
0.999850:0.126896,Am_ORA3/14-327:0.246275)0.898716:0.044675,
(((((Tr_ORA3/19-329:0.046624,Tn_ORA3/17-327:0.053390)0.999850:0.089710,
Ga_ORA3/19-328:0.094010)0.801132:0.023700,(((Ol_ORA3b/44-354:0.069572,
Ol_ORA3a/1-308:0.035903)0.999850:0.165337,(Fh_ORA3/4-314:0.027247,
(Pf_ORA3/15-325:0.013146,Xm_ORA3/15-325:0.069396)0.999850:0.033493)
0.999850:0.108108)0.989649:0.029358,(Hc_ORA3/17-322:0.026901,
On_ORA3/19-324:0.017972)0.999850:0.187065)0.999850:0.032602)0.926022:0.025716,

Gm_ORA3/14-321:0.193113)0.993940:0.036965,(Ss_ORA3b/19-332:0.000001,
Ss_ORA3a/19-295:0.000001)0.999850:0.166562)0.999850:0.072961)0.999850:0.221018)
0.999850:0.286331)0.754270:0.046175,((Cm_ORA3/1-305:0.587830,
Lc_V1R03/1-305:0.435647)0.742873:0.075983,((Am_ORA4/1-316:0.255356,
(Dr_ORA4/1-311:0.138997,((Ga_ORA4/1-320:0.151383,(((Ol_ORA4/1-322:0.139303,
(Tr_ORA4/1-321:0.029551,Tn_ORA4/1-320:0.057188)0.999850:0.082025)
0.858952:0.014562,Ss_ORA4/1-322:0.115946)0.989398:0.009803,
((Hc_ORA4/1-312:0.013341,On_ORA4/1-312:0.028329)0.999850:0.120732,
((Xm_ORA4/1-319:0.062598,Pf_ORA4/1-319:0.032279)0.999850:0.030776,
Fh_ORA4/1-318:0.042357)0.999850:0.036737)0.998859:0.020347)0.928807:0.014959)
0.999850:0.053549,Gm_Ora4/1-319:0.133832)0.996504:0.045142)0.995019:0.058330)
0.999850:0.205802,Lo_ORA4/1-317:0.168302)0.999850:0.386914)0.923614:0.067604)
0.999850:0.575203)0.997764:0.172013)0.969433:0.195984)0.999850:0.327016,
(((((Dr_ORA6/1-292:0.530390,Am_ORA6/1-294:0.437447)0.999850:0.133054,
(Gm_ORA6/1-295:0.465359,(((((Xm_ORA6/1-300:0.057075,Pf_ORA6/1-300:0.030828)
0.999850:0.070328,Fh_ORA6/1-290:0.463988)0.999850:0.206459,
Ol_ORA6/1-294:0.426793)0.943429:0.067516,(Hc_ORA6/1-297:0.035629,
On_ORA6/1-302:0.012507)0.999850:0.264463)0.967453:0.034732,
(Ga_ORA6/1-300:0.362072,(Tr_ORA6/1-297:0.064267,Tn_ORA6/1-295:0.086598)
0.999850:0.160149)0.935740:0.041535)0.995752:0.119868)0.999850:0.140737)
0.486476:0.059750,Ss_ORA6/1-106:0.730380)0.967121:0.166329,
Lo_ORA6/1-301:0.407695)0.999850:0.264482,Lc_V1R06/1-290:0.616847)
0.999850:0.612092)0.999850:0.614691,Lo_ORA5/1-294:0.227177)0.999850:0.152917,
Gm_ORA5/1-294:0.192129)0.925696:0.048964,(Ss_ORA5b/1-294:0.041105,
Ss_ORA5a/1-294:0.101137)0.999850:0.060593)0.955185:0.030527,
(Dr_ORA5/1-289:0.404683,(Am_ORA5b/1-293:0.250523,Am_ORA5a/1-295:0.185641)

0.995590:0.096371)0.999850:0.161105)0.999850:0.061690,Ga_ORA5/1-294:0.147018)
0.994044:0.036586)0.998545:0.021325)0.917902:0.042847,Ol_ORA5/1-292:0.294414)
0.999850:0.090063,Fh_ORA5/1-294:0.084234)0.999850:0.119472,
Xm_ORA5/1-293:0.068018,Pf_ORA5/1-293:0.049265);

Part 6 References

1. Saraiva LR, Korsching SI: **A novel olfactory receptor gene family in teleost fish**. *Genome Research* 2007, **17**(10):1448-1457.
2. Ota T, Nikaido M, Suzuki H, Hagino-Yamagishi K, Okada N: **Characterization of V1R receptor (ora) genes in Lake Victoria cichlids**. *Gene* 2012, **499**(2):273-279.
3. Nikaido M, Ota T, Hirata T, Suzuki H, Satta Y, Aibara M, Mzighani SI, Sturmbauer C, Hagino-Yamagishi K, Okada N: **Multiple Episodic Evolution Events in V1R Receptor Genes of East-African Cichlids**. *Genome Biology and Evolution* 2014, **6**(5):1135-1144.
4. Johnstone KA, Lubieniecki KP, Chow W, Phillips RB, Koop BF, Davidson WS: **Genomic organization and characterization of two vomeronasal 1 receptor-like genes (ora1 and ora2) in Atlantic salmon Salmo salar**. *Marine Genomics* 2008, **1**(1):23-31.
5. Johnstone KA, Lubieniecki KP, Koop BF, Davidson WS: **Identification of olfactory receptor genes in Atlantic salmon Salmo salar**. *Journal of Fish Biology* 2012, **81**(2):559-575.
6. Syed AS, Korsching SI: **Positive Darwinian selection in the singularly large taste receptor gene family of an 'ancient' fish, Latimeria chalumnae**. *Bmc Genomics* 2014, **15**.
7. Grus WE, Zhang J: **Origin of the Genetic Components of the Vomeronasal System in the Common Ancestor of all Extant Vertebrates**. *Molecular Biology and Evolution* 2009, **26**(2):407-419.
8. Venkatesh B, Lee AP, Ravi V, Maurya AK, Lian MM, Swann JB, Ohta Y, Flajnik MF, Sutoh Y, Kasahara M *et al*: **Elephant shark genome provides unique insights into gnathostome evolution (vol 505, pg 174, 2014)**. *Nature* 2014, **513**(7519).
9. Dong D, Jones G, Zhang S: **Dynamic evolution of bitter taste receptor genes in vertebrates**. *Bmc Evolutionary Biology* 2009, **9**.
10. Oike H, Nagai T, Furuyama A, Okada S, Aihara Y, Ishimaru Y, Marui T, Matsumoto I, Misaka T, Abe K: **Characterization of ligands for fish taste receptors**. *Journal of Neuroscience* 2007, **27**(21):5584-5592.
