# Supplementary material for: Tetrapod V1R-like ora genes in an early-diverging ray-finned fish species: the canonical six ora gene repertoire of teleost fish resulted from gene loss in a larger ancestral repertoire
Source: BMC Genomics. 2016 Jan 27;17:83. doi: 10.1186/s12864-016-2399-6 (PMC4728799; doi:10.1186/s12864-016-2399-6)
Supplement: Additional file 4: — A multiple sequence alignment of ora1-8 genes of ray-finned fish with exon/intron boundaries marked in color. Species are indicated by the initials of their Latin names, see Table 1 for full names. (PDF 70 kb) [file 12864_2016_2399_MOESM4_ESM.pdf]

CLUSTAL format alignment by MAFFT (v7.243)
Exon-exon borders are color-coded as follows: ora2 .XX. – ora3 .XX..XX..XX. – ora4 .XX.

```
Am-ORA1         --------------------------------------------MDLC----ITIKGVSF
Fh-ORA1         --------------------------------------------MDLC----VTIKGVSF
Pf-ORA1         --------------------------------------------MDLC----VTIKGVSF
Xm-ORA1         --------------------------------------------MDLC----VTIKGVSF
Sc-ORA1         ------------------------------------------------------------
Sr-ORA1         ------------------------------------------------------------
Hc-ORA1         --------------------------------------------MDLC----VTIKGVSF
On-ORA1         --------------------------------------------MDLC----VTIKGVSF
Ol-ORA1         --------------------------------------------MDLC----VTIKGVSF
Ga-ORA1         --------------------------------------------MDLC----VTIKGVSF
Ss-ORA1         M-------------------------------------------LDLC----VTIKGMSF
Dr-ORA1         --------------------------------------------MDLC----VTIKGVSF
Gm-ORA1         --------------------------------------------MDLC----VTIKGVSF
Lo-ORA1         --------------------------------------------MDLC----ITIKGVSF
Lo-ORA8a        -------------------------------------------G----------------
Lo-ORA8b        ------------------------------------------------------------
Lo-ORA7         --------------------------------------------MDLQ----NLAKAVAT
Am-ORA2         --------------------------------------------MDLY----FLTRGLLY
Fh-ORA2         MRG---------------------NHCLSGTRSASELPAFLPSAMPSD----KLVRGWLF
Pf-ORA2         ------------------------------MIFYLYLLFSLSAGMPSN----VNVRGMLF
Xm-ORA2         --------------------------------------------MPSN----EDIRGMLF
Gm-ORA2         --------------------------------------------MPSE----ELIRAMLY
Ga-ORA2         --------------------------------------------MPSE----MFVRGMLY
Hc-ORA2         --------------------------------------------MASE----VFVRGMLF
On-ORA2         --------------------------------------------MASE----VFVRGMLY
Ss-ORA2         --------------------------------------------MQSE----EVVRGMLY
Ol-ORA2         M-----------------------------AHSFVFLEPFFCAGMPSD----DLVRRGLY
Tn-ORA2         MIV---------------------------VTIILRDLLSISPGMQST----EFVRGILY
Tr-ORA2         MKD----------------------Y----GGIILRDLFSISTGMQSV----EFVRGILY
Dr-ORA2         --------------------------------------------MIAE----AVIRGLLF
Lo-ORA2         --------------------------------------------MDPQ----VVIRGMLY
Am-ORA3         -------------------------------MWVNGTVTIIKATGGQL----SSVPMALY
Fh-ORA3         -----------------------------------------MGLRTPV----SPAQSAFY
Pf-ORA3         ------------------------------MQQESTTKLLGMGLRTGV----SPAQSAFY
Xm-ORA3         ------------------------------MEQETTTKLVGMGFRTAV----SPAESDFY
Ga-ORA3         MAE--------------------------HIGEDGESTLIGMGLRVSV----SPVQTAFY
Tn-ORA3         MAA----------------------------NTEDDTEVVGMGLRVSV----SPVQTASY
Tr-ORA3         MAV--------------------------NSMDEDDMELVGMGLRVSV----SPFQTAFY
Ol-ORA3a        -----------------------------------------------M----SALQAVFY
Ol-ORA3b        MSAVSRMKTQLDLRPADEAAAKIKDF-VGPEGRDENPKHVGIGLQVPV----SDVQIVCH
Gm-ORA3         -------------------------------MSEAEAELVGMGLRTDA----SPVQTTFY
Hc-ORA3         MSS--------------------------LSDQRK--DLAGMRMHIFV----SPAQTAFY
On-ORA3         MSS--------------------------LSDQRKEPDLAGMGMRIFV----SPAQTAFY
Ss-ORA3a        MET--------------------------PMPELKELEPVGVGLRVTT----SPTQTTFY
Ss-ORA3b        MET--------------------------PMPELKELEPVGVGLGVNR----YPFQNALY
Dr-ORA3a        --------------------------------MAPQKKPVNISQRITS----SPFYIMLY
Dr-ORA3b        --------------------------------MATTAKPLTVSQRALS----SPLYIAFY
Lo-ORA3         --------------------------------MGEQTNKVVTVLLKAA----IPAQNALY
Am-ORA4         --------------------------------------------M-EL----LTIEAILF
Fh-ORA4         --------------------------------------------MSEI----LTVDAILF
Pf-ORA4         --------------------------------------------MSEI----LTVDAILF
Xm-ORA4         --------------------------------------------MAEV----LTVEAILF
Hc-ORA4         --------------------------------------------MSKV----LTVEAILF
On-ORA4         --------------------------------------------MSEV----FTVEAILF
Ss-ORA4         --------------------------------------------MSEV----LTVDAILF
Ol-ORA4         --------------------------------------------MSKV----LTLDAILF
Tn-ORA4         --------------------------------------------MSEV----LTVDAILF
Tr-ORA4         --------------------------------------------MSEV----LTVDAILF
Gm-Ora4         --------------------------------------------MAEV----LTVDAILF
Ga-ORA4         --------------------------------------------MSTR----SNLHALRR
Dr-ORA4         --------------------------------------------MSEV----LTVDAVLF
Lo-ORA4         --------------------------------------------MAQV----LPVDAILF
Am-ORA6         --------------------------------------------MESF----IFGLLVLR
Dr-ORA6         --------------------------------------------MEQI----QVNLLSLR
Fh-ORA6         --------------------------------------------MCHL----SVYLFGLK
Pf-ORA6         --------------------------------------------MSDL----SVDLQGLR
Xm-ORA6         --------------------------------------------MSHL----SVDLLGLR
Ga-ORA6         --------------------------------------------MVGL----SVDLLGLK
Hc-ORA6         --------------------------------------------MAEL----SVNLLGLR
On-ORA6         --------------------------------------------MAEL----SVNLLGLR
Tn-ORA6         --------------------------------------------MLAL----SVQLLATR
Tr-ORA6         --------------------------------------------MLGL----SVQLLATR
Gm-ORA6         --------------------------------------------MAKVEYKTMITLVTIR
Ol-ORA6         --------------------------------------------MEGI----YVNLLGLR
Lo-ORA6         --------------------------------------------MDLM----NPVLLVLR
Ss-ORA6         --------------------------------------------M-----VDTLELLVFR
Am-ORA5a        --------------------------------------------MNAK----EWIKSTIR
Am-ORA5b        --------------------------------------------MDAE----GWIKSVIR
Fh-ORA5         --------------------------------------------MDAD----ELIESIIR
Pf-ORA5         --------------------------------------------MDAD----KLVESIVR
Xm-ORA5         --------------------------------------------MDAD----KLIQSIVR
Gm-ORA5         --------------------------------------------MDVD----DIIESAVR
Ga-ORA5         --------------------------------------------MDAE----GWIESLIR
Tn-ORA5         --------------------------------------------MDTK----ELAESIIR
Tr-ORA5         --------------------------------------------MDAE----ELVESIVR
Hc-ORA5         ----------------------------------------------ME----GLIEAIIR
On-ORA5         ----------------------------------------------ME----ELIESIIR
Ss-ORA5a        --------------------------------------------MDAT----EWIEAFIR
Ss-ORA5b        --------------------------------------------MDAK----DWIEALIR
Ol-ORA5         --------------------------------------------MELN----KPIASVIS
Dr-ORA5         --------------------------------------------MQLQ----DWVESSIR
Lo-ORA5         --------------------------------------------MDTV----GVIESTVR
```

```
Am-ORA1         LLQTGLGI---MGNMLVLAAYGHIAL-VE-PRLQPVDQIMAHLAFANLML--------LL
Fh-ORA1         LLQTGMGI---LGNGVVLLAYASIIC-TE-PKLLPVDMILCHLAFANLML--------LL
Pf-ORA1         LLQTGLGI---LGNVVVLLAYASIIC-TE-PKLLPVDMILCHLAFANLML--------LL
Xm-ORA1         LLQTGLGI---LGNAVVLLAYASIIC-TE-PKLLPVDMILCHLAFANLML--------LL
Sc-ORA1         ------------------------------------------------------------
Sr-ORA1         ------------------------------------------------------------
Hc-ORA1         LLQTGMGV---LGNTVVLLAYTHIVC-TG-PKLLPVDMILCHLAFTNLLL--------LL
On-ORA1         LLQTGMGI---LGNTVVLLAYTHIVC-TG-PKLLPVDMILCHLAFANLLL--------LL
Ol-ORA1         LLQTGLGI---LGNSVVLLVYSHIMC-TG-PKLLPVDMILCHLAFANLIL--------LL
Ga-ORA1         LLQTGMGI---LGNTVVLLAYAQLIY-AE-PKLLPVDMILCHLAFANLML--------LL
Ss-ORA1         LLQTGLGF---LGNTLVLLAYTQVVC-SE-CRLQPVDIILCQLAFVDLIL--------IL
Dr-ORA1         LLQAGLGI---LANALVLLAYAHIRL-AE-ARLQPVDAILCHLALVDLLL--------LL
Gm-ORA1         LLQTGLGV---LGNALVLLAYVHIAHGAD-HKLLPTDLILCHLAFSNLVL--------LL
Lo-ORA1         LLQTGLGI---LGNLLVLLAYAHIAC-SD-GRVQPVDKILCHLAFANLLL--------LL
Lo-ORA8a        ------------------------------------------------------------
Lo-ORA8b        ------------------------------------------------------------
Lo-ORA7         LLQNMVGI---PANLTVLGVFVHVAR-TE-RRLLPTDAIVSHLVSVNLLL--------IL
Am-ORA2         LFLPVFGV---PGNCAVIWAFLLALR-QE-GTLLPADAIVLHLACANLLV--------VS
Fh-ORA2         LSLAVVGI---PGNIAVIVAFLLLIL-QE-CFLLAADAIVLHLAFANLLV--------VL
Pf-ORA2         LSLTVVGV---PGNMAVIVAFLLLVL-QE-SWLLAADAIVLHLSCTNLLV--------VL
Xm-ORA2         LSLTVVGV---PGNMAVIVAFLLLIL-QE-SCLLAADAIVLHLSCTNLVV--------VL
Gm-ORA2         LTLTVVGV---PGNLAVIWAFLLALH-QE-RRLLPADTILLHLASVNLLV--------VG
Ga-ORA2         LSLTVLGV---PGNATVILAFLLLLY-QE-RRLLPSDAIVLHLAFVNLLV--------VA
Hc-ORA2         LFLTVVGI---PGNATVIVAFLLLLY-QE-KRLLAADSILLHLACVNLLV--------VV
On-ORA2         LSLTIVGI---PGNATVIVAFLLLLY-QE-KRLLAADAILLHLACVNLLV--------VV
Ss-ORA2         LSLTVVGV---PGNTAVIVAFLLALY-QE-HQLLPADAIVLHLACANLLV--------VG
Ol-ORA2         ASLTIVGV---PGNILVIMAFLLLSY-EE-NRLLAAEAIVLHLTCANLLV--------VV
Tn-ORA2         LSLAVVGA---PGNTCVILAYFILLY-QE-KRLLPADVIILHLACANLLV--------VV
Tr-ORA2         LSLTVVGA---PGNICLILAYLILLH-QE-NRLLPADVIILHLSCVNLLV--------VV
Dr-ORA2         LSLVLVGV---PGNTAVICGFILLVR-RE-GRLSPADAIVLHLCSANLVV--------VS
Lo-ORA2         LFLVVVGV---PGNLAVIWAFCHIMR-SE-RKLMPADAIVLHLAAVNLLV--------AA
Am-ORA3         MILVLLGI---FGNAIVISVVGESILREP-GGGRNSDMILVNMAFSNLMV--------SM
Fh-ORA3         IILVALGI---VGNAIVIGVIGKSVMVDH-GGGHNSDIIIINLAVSNFMV--------SV
Pf-ORA3         IILVALGI---VGNSIVIGVIGKSVLMDR-GPGHNSDIIIVNLAVSNLMV--------SI
Xm-ORA3         IILVALGI---VGNSIVIGVIGKNVMMDR-GPGHNSDIIIVNLAVSNLMV--------SI
Ga-ORA3         IMLVTLGI---LGNFTVVGVIGKSIATDH-VGGRNSDIIIINMALSSLLV--------SV
Tn-ORA3         IFLVLLGI---LGNTTVVGVIGKSILMDR-GGGRNSDIIIVNMALSNLLV--------SL
Tr-ORA3         IFLVLMGI---LGNATVVGVIGKSIIMDR-GGGRNSDIIIVNMALSNLLV--------SL
Ol-ORA3a        AILVVLGV---LGNTTVIVVVGKSVIQDR-RVAHNSNIIIINMAASNLMV--------SV
Ol-ORA3b        VIMVVLAI---LGNATVIVVIGKSVIQDR-RVAHNSNIIIINMAVSNLMV--------SI
Gm-ORA3         ILLVLFGI---VGNTTVIGVIGHSVLMNP-GVGRNSDIIIINMAVSNLMV--------SV
Hc-ORA3         IILVIMGI---LGNTTVILVIGKSIILEH-NWGRNSDIIIVNMAMSNLLV--------SL
On-ORA3         IILVIMGI---LGNTTVILVIGKSIILEH-NWGRNSDIIIVNMAMSNLLV--------SL
Ss-ORA3a        IILVLLGI---VGNTTVIGVMLDSVFKDP-SGVRNSDIILMNMALSNLLV--------SV
Ss-ORA3b        IIFVLLGI---VGNATVVGVISESVFKDP-SGGRNSDIILINMALSNLLL--------SL
Dr-ORA3a        VVLVLLGN---AGNTTVIAVVGQSLLQET-GTVRSSDVILVNMAFSNLMV--------SL
Dr-ORA3b        VILVLLGN---LGNSLVIGVVGEGLLREP-GVARSSDIILVNMALSNLMV--------SL
Lo-ORA3         GLLVMLGI---VGNGLVMGVVGRGLVKEG-LARQHSDIILLNLVLSNLLV--------SL
Am-ORA4         GFLVFSGI---LGNMLVIYAVFQCALDNPSHHLSPSDIILLNISMANLLT--------SM
Fh-ORA4         GLLVFSGI---LGNILVIHVVFQSALESPSRRLPPSDTILVHLSLANLLT--------SL
Pf-ORA4         GLFVFSGI---LGNILVIHVVFQSALESPSRRLPPSDTILVHLSLANLLT--------SL
Xm-ORA4         GLLVFSGI---LGNILVIHVVFQTAFESPSRRLSPSDTILVHLSLANLLT--------SL
Hc-ORA4         GVLVFSGI---LGNILVIYVVFQSVTKTPPRRLPPSDLILVHLSLANLLS--------SL
On-ORA4         GLLVFSGI---LGNILVIYVVFQSVTKTPPRRLPPSDTILVHLSLANLLS--------SL
Ss-ORA4         GFLVFSGI---LGNILVIHVVFQSAIESLSRRLPPSDTILVNLSLANLLT--------SL
Ol-ORA4         GLLVFSGL---LGNTLVIYAVFQSAFETPPGRLSPSDTILVHLSLANLLT--------SL
Tn-ORA4         GLLVFSGI---LGNILVIHVVLQAAFQGTSGRLPLSDTILVHLSLANLLT--------SL
Tr-ORA4         GLLVFSGI---LGNFLVIHVVLQAAFQSASRRLPLSDTILVHLSLANLLT--------SL
Gm-Ora4         GLLVFTGI---MGNIMVMHTVCQSAMQSQSRRMPASDTILVHLSLANLLT--------SL
Ga-ORA4         S-LMFSDAQRNCGKCLRLH-VFQSAFESPSRRLPPSDTILVHLSLANLLT--------SL
Dr-ORA4         GLLVFSGI---IGNIM----VFDCAKLCASRHLPPSDTILVHLCLANLLT--------SV
Lo-ORA4         GVLVLSGI---VGNVLVICAVVQSVLQNSLLRIPPSDLILANLSLANLLT--------SF
Am-ORA6         IMLSVIGV---LGNTVLIVSILQM------TRLKTFEVFLLGLAVSNLEE--------IM
Dr-ORA6         LFISIIGV---VGNTLLLVSILHTH-----THLKSFELFLLALCSANLQQ--------LV
Fh-ORA6         TFISCIGF---VGNIFLIVSIFQTAV----SHVKPFELFLLGLASANLEE--------IV
Pf-ORA6         MFVSCVGL---VGNIFLILSIFQTRV----SNIKSFELFLLGLASVNLEE--------IV
Xm-ORA6         LFVSCVGL---VGNIFLILFIFQTRV----SHIKSFELFLLGLASFNLEE--------IV
Ga-ORA6         VFILCVGL---MGNVFLMVAVAQTKF----PRVKSFELFLLGLAAANLEE--------IA
Hc-ORA6         LVFSSIGL---MGNTVLIASIIKINF----FHIKSFEIFLFGLAAANLWE--------IV
On-ORA6         LVFSSVGL---MGNTILIASIIKINF----FHIKSFEIFLFGLAAANLWE--------IV
Tn-ORA6         IIISCIGI---FGNVFLIISVVQTKF----SRIKSFELFLLELAAANLEE--------IF
Tr-ORA6         IIISCIGI---FGNVFLIVSVVQNKF----SQIKSFELFLLELAAANLEE--------IL
Gm-ORA6         FVMSLIGI---MGNMFLVFVIFQTKI----SRIKSFEVFLLGLAVSNLEE--------LV
Ol-ORA6         IAVSFTGL---VGNVCLILSIIHVKW----SHIKSFEVFLLGLAAANLEE--------IV
Lo-ORA6         YLISIVGI---IGNITLVVSILSH------SHMKTFEIFLLGLSFSNLEG--------IF
Ss-ORA6         IFISTVGI---VGYVVLILSLIKNQI----SCLKTFEVFLLGLAASNLVAPNHNVHPHHL
Am-ORA5a        GFMCVSGI---LGNNWLGFCSLPKSR----SQLRTNNILFINLAISNLIT--------NY
Am-ORA5b        GLMCVSGI---IGNHWLGFSALPKSR----AHLKTNDILFVNLASSNLIT--------NY
Fh-ORA5         ALMFIAGI---LGNNWLAITSLPKRR----SDIRTNEILLVNLAVSNLIT--------NC
Pf-ORA5         ILMFIAGI---LGNNWLAIASLPRKK----SEIRTNEILLINLAVSNLIT--------NY
Xm-ORA5         ILMFIAGI---LGNNLLVIASLPRKK----SEIRTNEILLINLAVSNLIT--------NY
Gm-ORA5         ALMFLLGM---LGNNWLAVRSIPSRL----SALRTNELLFLNLAVSNLIT--------NY
Ga-ORA5         ALMFLAGI---LGNNWLAIRSLPGHK----SSIRTNEVLFINLAVSNLIT--------NY
Tn-ORA5         GMMFLAGI---LGNNYLAARSFPTQR----TSIRTNEVLFINLAVSNLIT--------NY
Tr-ORA5         GLMFLAGV---LGNNWLAVRSFPTQR----SSVRTNEVLFLNLALSNLIT--------NY
Hc-ORA5         ALMFIAGI---LGNNWLAICSLPRHK----SSIRTNEVLFINLAISNLIT--------NY
On-ORA5         GLMFIAGI---LGNNWLAICSLPRHK----SSIRTNEVLFINLAISNLIT--------NY
Ss-ORA5a        GLMCLLGI---LGNNWLCLRSLPGPK----SSLRTNEVLFINLAVSNLIT--------NY
Ss-ORA5b        GLMCLVGI---LGNNWLGLRSLPGPK----SHLRTNELLFINLAVSNLIT--------NY
Ol-ORA5         ALMFLASI---LGNNWLAVASLPKDR----SAIRTNEVLFINLAVSNLIT--------NY
Dr-ORA5         AFFCVTGI---TGNFWLALRSLPRSR----SRLRPNDVLFINLAVSNLIT--------NC
Lo-ORA5         ASMCFLGI---MGNSMLVLHSLPSKR----SHLKTSEVLFINLAASNLIT--------NC
```

```
Am-ORA1     TRGVPQTMTVFGLRHLLNDSG------------------------------CKVVIY--T
Fh-ORA1     TRCVPQTMTVFGLKGLLNDPG------------------------------CKVVIY--A
Pf-ORA1     TRCVPQTMTVFGLKDLLNDPG------------------------------CKVVIY--A
Xm-ORA1     TRCVPQTMTVFGLKDLLNDPG------------------------------CKAVIY--A
Sc-ORA1     ------------------------------------------------------------
Sr-ORA1     ------------------------------------------------------------
Hc-ORA1     TRSVPPSMTVFGLKALLNDPG------------------------------CKVVIY--A
On-ORA1     TRCVPQTMTVFGLKDLLNDPG------------------------------CKVVIY--A
Ol-ORA1     TRCVPQTMTVFGLKDLLNDPG------------------------------CKVVIY--A
Ga-ORA1     TRCVPQTMSVFGLRDLLGDPG------------------------------CKVVIY--A
Ss-ORA1     TRCIPQTMTVFGLRDLLNDPG------------------------------CKVVVY--S
Dr-ORA1     TRGVPQTMTVFGMRNLLDDTG------------------------------CKVVIY--T
Gm-ORA1     TRCVPQTMTVFGLHDLLDDAG------------------------------CKVVIY--L
Lo-ORA1     TRCVPQTMTVFGLKDLLNDSG------------------------------CKAVIY--A
Lo-ORA8a    ------------------------------------------------------------
Lo-ORA8b    ------------------------------------------------------------
Lo-ORA7     TRGIPQSLSALDYRGFYDSAT------------------------------CKFLIF--T
Am-ORA2     CRCVFEVFANFQVFNGFNDPG------------------------------CKGIYF--I
Fh-ORA2     VRCLLEALASFHLANVFGDVG------------------------------CKAVIF--I
Pf-ORA2     VRCLMETLASFHLANVFGDVG------------------------------CKAVIF--I
Xm-ORA2     VRCLMETLASFHLANVFGDIG------------------------------CKGVIF--I
Gm-ORA2     VRCLLETLASFRLASVFGDTG------------------------------CKSVIF--V
Ga-ORA2     ARCLPETLASFRLSGIFGDVG------------------------------CKAVIF--V
Hc-ORA2     VRALTETLASFRLADIFGDTG------------------------------CKSVIF--I
On-ORA2     VRALTETLASFRLADIFGDTG------------------------------CKSVIF--I
Ss-ORA2     VRCLLETLATFRLVNIFGDTG------------------------------CQGVIF--V
Ol-ORA2     VRCLTETLASFHVVNVFGDAG------------------------------CKGVIF--I
Tn-ORA2     ARCFLEFLASFRLALIFGDVG------------------------------CKSVIF--V
Tr-ORA2     ARCLLEFLASFHLAIIFGDVG------------------------------CKSVIF--V
Dr-ORA2     VRCLLEVLATFRIHNVFDDAG------------------------------CRAVIF--L
Lo-ORA2     VRCSFEALAAFGVLYVFNNTG------------------------------CKTIIF--I
Am-ORA3     TRNMLLVISDTGLEVLPGKDW------------------------------CQILMG--V
Fh-ORA3     VRNVLLIVSDLGIQMYSSKGW------------------------------CQFLMG--V
Pf-ORA3     VRNLLLIISDLGFKLYSSKGW------------------------------CQFLMG--V
Xm-ORA3     MRNLLLIISDLGFKLYSSKGW------------------------------CQFLMG--V
Ga-ORA3     MRNIPLVISDIGLELYSSKEW------------------------------CQVLMG--L
Tn-ORA3     MRNTLLILSDLGLEMYSSKEW------------------------------CRFLMG--V
Tr-ORA3     MRNMLLILSDIGLEMYSSKEW------------------------------CQFLMG--V
Ol-ORA3a    MRNILLVMSDFGIQLFLSRER------------------------------CQFLMG--V
Ol-ORA3b    MRNILLIVSDFGIQLFLSRER------------------------------CQFLMG--V
Gm-ORA3     LRNALLVISDIGIALYSSKEC------------------------------CQFLMG--V
Hc-ORA3     LRNTLLIISEFGLQIYTAKGF------------------------------CQLLMG--M
On-ORA3     LRNTLLIISEIGLQIYTTKGF------------------------------CQLLMG--M
Ss-ORA3a    LRNVLLVISDLGLELNTSRDG------------------------------CHVLMG--V
Ss-ORA3b    LRNILLVISDLGLELNTSRDG------------------------------CHVLMG--V
Dr-ORA3a    LRNTVLMVSDLGVEIFLSRDM------------------------------CQFMMG--L
Dr-ORA3b    TRNSLLVISDMGVQVFLNRNW------------------------------CRFMMG--I
Lo-ORA3     VRNIPLLLADVGLQLFTSPGC------------------------------CQFLMF--M
Am-ORA4     FRTIPIFISDLGLKVSLDTNW------------------------------CRVFML--L
Fh-ORA4     FRTVPIFVSDLGLDVSLSAGW------------------------------CRLFML--L
Pf-ORA4     FRTVPIFMSDLGLDVSLSPGW------------------------------CRIFML--L
Xm-ORA4     FRTVPIFVSDLGLDMSLSPVW------------------------------CRIFML--L
Hc-ORA4     FRTVPIFVSDLGLDLYLSSGW------------------------------CRVFML--L
On-ORA4     FRTVPIFVSDLGLDLSLSSGW------------------------------CRVFML--L
Ss-ORA4     FRTVPIFVSDLGLDVSLSQGW------------------------------CRLFMF--L
Ol-ORA4     FRTVPIFVSDLGLDVSLSPGW------------------------------CRVFML--L
Tn-ORA4     SRTVPIFVSDLGRDVSLSAGW------------------------------CRVFML--L
Tr-ORA4     FRTVPIFVSDLGSDVSLSPGW------------------------------CQVFML--L
Gm-Ora4     FRTVPIFISDLGLEVTLSPGW------------------------------CRVFML--L
Ga-ORA4     FRTVPIFVSDLGLDVSLSLGW------------------------------CRIFML--L
Dr-ORA4     FRTVPIFVSDLGLQVWLTAGW------------------------------CRVFML--L
Lo-ORA4     FRTVPIFVSDLGLEVSLAPGW------------------------------CRLFMF--L
Am-ORA6     IVDIYDMIVLRST-HSISILS------------------------------CGVLKF--M
Dr-ORA6     MVDVYDVLLLCSP-SCIGVCS------------------------------CRALRF--L
Fh-ORA6     IVNIYDVHVLEVFSATAGSWR------------------------------CRLIGF--M
Pf-ORA6     IINVYDVIILDTVSTTTGAWW------------------------------CRLLKF--M
Xm-ORA6     SINVYHVIILDTVFTTTGAWW------------------------------CRLLKF--M
Ga-ORA6     ITTVFDVDFLQASSRGVDTWS------------------------------CRSLKF--L
Hc-ORA6     IINIYDIIILQTPSTATGTWS------------------------------CYLLEF--V
On-ORA6     ITNIYDIIILQTSSTATGTWS------------------------------CYLLEF--M
Tn-ORA6     IVNVYDIILLCTSYATVGTWS------------------------------CRTLKF--L
Tr-ORA6     IVNIYDMIILQTSFATVGTWS------------------------------CRLLKF--L
Gm-ORA6     VVDFYEVIMLIG--HIQNSLL------------------------------CRTMKF--L
Ol-ORA6     ILNVYDAFMLQTS--SSDTWW------------------------------CRFLKF--M
Lo-ORA6     LVSIFDITTRLAL-QSLEEWS------------------------------FKILRF--M
Ss-ORA6     VLPAPQVL--HNGLAFLGQWWAISRLLLVLLVSYTAEGYMSLRPGQAEIKLCVWLYLFGM
Am-ORA5a    MVDLPDTL-ELVKRWPVGRMY------------------------------CSAFNF--F
Am-ORA5b    LVDLPD-MMDFTYNFLMGQMY------------------------------CSVFNF--C
Fh-ORA5     LVDAPDTMADFAGRWFLGATF------------------------------CGIFRF--S
Pf-ORA5     LVDIPVTMEDFAGQWLLGLTF------------------------------CGIFRF--S
Xm-ORA5     LVDIPVTMEDFAGRWFLGLSF------------------------------CGIFHF--S
Gm-ORA5     LVDLPDTMADIAGGWFLGDGY------------------------------CGVFRF--C
Ga-ORA5     LVDLPDTVADFAGHWFLGETF------------------------------CAAFRF--C
Tn-ORA5     LVDLPDTMADFAGRWFLGETY------------------------------CGIFRF--C
Tr-ORA5     LVDLPDTVADFAGRWFLGETY------------------------------CGIFRF--C
Hc-ORA5     LVDLPDTMADFAGRWFLGKTY------------------------------CGVFCF--C
On-ORA5     LVDLPDTMADFAGRWFLGETY------------------------------CGVFCF--C
Ss-ORA5a    LVDLPDTMADFVGHWFLGEAY------------------------------CCVVQF--C
Ss-ORA5b    LVDLPDTMADFAGRWFLGEAY------------------------------CGVFRF--C
Ol-ORA5     VVNLPETMADIADNWFLGETF------------------------------CCVFLF--S
Dr-ORA5     MVDLPDTLAQFLNSWLLSRNY------------------------------CSVLQF--S
Lo-ORA5     LVDLPDTLADIAGRWFLGEAY------------------------------CGIFLF--C
```

```
Am-ORA1      YRITRALSVCFTCMLSVFQALTIAPAGGPRL---ARLKARLPQLVAPTFAGLWLLNMAVC
Fh-ORA1      YRVGRALSVCITCMLSVFQAATIAPA-GPRL---SRLKPTLPSLVLPTFAMLWFLNMAIC
Pf-ORA1      YRIGRALSVCITCMLSVFQAMTIAPA-GPRL---SKLKPVLPSLVLPTFAALWLLNMAIC
Xm-ORA1      YRIGRALSVCITCMLSVFQAMTIAPA-GPKL---SKLKPMLPSLVLPTFAALWLLNMAIC
Sc-ORA1      ---------CITCMLSVFQAVTIAPA-GPRL---SRLKPALPSLVLPTFALLWFLNMAVC
Sr-ORA1      ---------CITCMLSVFQAVTIDPA-GPRL---SRLKPALPSLVLPTFALLWFLNMAVC
Hc-ORA1      YRIGRALSVCITCMLSVFQAVTITPT-GPYL---SRLKPSLPSLVIPTFAGLWLFNMAIC
On-ORA1      YRIGRALSVCITCMLSVFQAVTITPT-GPYL---SRLKPSLPSLVLPTFAGLWLFNMAIC
Ol-ORA1      YRIGRALSVCITCMLSVFQAVTIAPA-GPFL---SRLKLALSSLVFPTFVGLWLLNMAVC
Ga-ORA1      YRIGRALSVCVTCMLSVFQAVTLAPA-GPRL---SRLKPALPSLVLPTSAGLWLLNMAVC
Ss-ORA1      YRIARALSVCITCMLSVFQAVTIAPAGGPCL---SRLKAQLPSLIVPTIAGLWLFNMAVC
Dr-ORA1      YRIARALSVCITCMLSVFQAVTVAPAAGPLL---SGVKARLPQLLAPTFAALWFINMAVC
Gm-ORA1      YRITRALSVCLTCMLSVFQAATLAPD-AP------RLKAALPALVLPSFAGLWLLNMAVC
Lo-ORA1      YRIARALSVCITSMLSVFQSIIIAPA-SSRW---VGLKVRVSQLLFPSFAALWLINMAVC
Lo-ORA8a     ------------------------------------------------------LNVAVC
Lo-ORA8b     ------------------------------------------------------------
Lo-ORA7      YRTTRAMSISLTFVLSAYQCITIAPA-SSRL---SRLKPWLYRCLLPLNLFFWLLNGGTT
Am-ORA2      YRTFRGLSIWLTFTLSSYQCLSIAPP-GSHW---ATLRSLFGRYLWLIFLLLWIINTSAS
Fh-ORA2      YRTSRALSIWLTFLLSAYQCLSIAPP-GSSW---ASARVLVAQNLPAVFFILWVLHTSMS
Pf-ORA2      YRTSRALSIWLTFLLSAYQCLSIAPP-GSKW---ASVRILVAQNLPIVFLFLWVLHTSTS
Xm-ORA2      YRTSRALSIWLTFLLSAYQCLSIAPP-GSKW---ASLRTLVAQSLPIVFVFLWVLHSSLS
Gm-ORA2      YRTARSLSIWLTFVLSAYQCLSIAPP-GSRW---AAARALAARYMAAIFLALWLGNTCMS
Ga-ORA2      YRTSRSLSIWLTFVLSAYQCLSIAPP-GSRW---AHLRVLLAQYLGLVFLILWLLNTCMS
Hc-ORA2      YRATRGLSIWLTFLLSTYQCLSIAPP-GSSW---ASVRALLGHYLAFVFLFLWVLNACMT
On-ORA2      YRTTRALSIWLTFLLSTYQCLSIAPP-GSSW---ASVRALLGHYLAFVFLFLWVLNTCMT
Ss-ORA2      YRTSRSLSIWLTFVLSAYQCLSIATP-GSRW---ASIRVLVARYLAVIFLTLWVINTSMS
Ol-ORA2      YRTSRGLSIWLTFLLSTYQCLSVSPP-GSYW---ASVRVLLAQNLVFVFLFLWVLNTTMS
Tn-ORA2      YRTSRSLSIWLTFILSAYQCLCIAPL-GSQL---ATLRMIVAKYLFYVFFFLWLLTTTMS
Tr-ORA2      YRTSRSLSIWLTFILSAYQCLCIAPP-GSQW---TTLRIVFASYLFYVFFFLWLLTTSMS
Dr-ORA2      HRTARSLSIWLTFLLTALQCLSVAPP-GSRR---AAARALLARSLPAIFLALWLINTSMS
Lo-ORA2      YRTSRSLSIWLTFVLSTFQCISIVPP-GSRG---YSIKSHAPRYLGGVFVFLWILNSWLS
Am-ORA3      WVWLRSVNVWSTFFLSAFHFHTLRRT-APPI-TSLSGPRGLPRGILTGFGLIWSSNLLYS
Fh-ORA3      WVWLRSVNVWSTFFLSAFHLHTLKRV-APTI-GNIQGPWSTYRTLLLSLGVIWILNLLYS
Pf-ORA3      WVWLRSVNVWSTFFLSAFHLHTLKRV-APTI-GHLQGPWSTYRTLLLSLAIMWILNLLYS
Xm-ORA3      WVWLRSVNVWSTFFLSAFHLHTLKRV-TPTI-GDLQGPRSTYRTLLLSLAIIWILNFLYS
Ga-ORA3      WVWLRSVNVWSTLFLSAFHLQTLRRV-APTA-VSRNGPRGLPKTLLLSLTLIWLLNLVYS
Tn-ORA3      WVWLRSVNVWSTLFLSAFHFQTLRRV-APVA-GPVQGARGAPKILLINLFLIWFINLIYS
Tr-ORA3      WVWLRSVNVWSTLFLSAFHLQTLRRV-APMA-VNVTGSRGAPKILLMNMFLIWFINLLYS
Ol-ORA3a     WVWLRSVNVWSTFYLSVFHLQTLRRV-APSV-GNLQASRGVPKTLLLNLLSIWILNLLYS
Ol-ORA3b     WVWLRSVNVWSTFYLSVFHLQTLRRV-APSV-GNLQASRGVPKTLLLNLLSIWLLNLLYS
Gm-ORA3      WVWLRSVNVWSTLLLSAFHFHTLRRV-APPL-GNLHGPRGLPKLLLLGLGLIWVLNFLYS
Hc-ORA3      SVWLRSVNAWSTLFLSAFHLQTLKRV-AP---GATNGPRGAPKTLLMCLGLIWIGNLIYS
On-ORA3      SVWLRSVNAWSTLFLSAFHLQTLKRV-AP---GATNGPRGVPKTLLVCLGLIWIGNLIYS
Ss-ORA3a     WVWLRSVNVWSTLFLSAFHFQTLRRV-APPP-GTVHGPRRPPKTLLISLGLIWFLNLIYA
Ss-ORA3b     WVWLRSVNVWSTLFLSAFHFQTLRRV-APPS-GTVHGPRRPPKTLLISLGLIWFLNLIYA
Dr-ORA3a     WVWVRSANVWSTFFLSAFHFQTLRRV-APPV-INLHGPRGPPLSLILGFCLIWSLNLIYS
Dr-ORA3b     WVWVRSANVWSTFFLSAFHFQTLRRV-APPV-SNVHGHPGPPRSLIFGLCLIWSLNLIYS
Lo-ORA3      WVWLRSVNVWMTMCLSAFHFLTLCRL-GPVVPAGPHGPRASLQRLLLVLALIWSLNLLYS
Am-ORA4      WVWWRAVGCWATLTLSAFHYATLKRK-RVST--CPQALRKDRRLTWGALGLVWGTNLLFS
Fh-ORA4      WVWWRAVGCWVTLTLSFFHCATLRRH-HVSL--GPLTLQRERRRVWVVLALVWGANLAFS
Pf-ORA4      WVWWRAVGCWVTLTLSIFHCATLRRQ-HVTF--GPLTLQRERRRVWVVLGLVWGANLAFS
Xm-ORA4      WVWWRAVGCWVTLTLSIFHCTTLRRQ-HVTF--GPLTLQRERRRVWVVLGLVWGANLVFS
Hc-ORA4      WVWWRAVGCWVTLTLSAFQCTTLRRQ-NVAF--GPLTVQRE-RRLWVVLGVVWGANLAFS
On-ORA4      WVWWRAVGCWVTLTLSVFQCTTLRRQ-NVAF--GPITVQRERRRLWVVLGVVWGANLAFS
Ss-ORA4      WVWWRAVSCWVTLTLSAFHCATLKRQ-HVAM--GPLAQEHERRKVWVALGLVWGLNLAFS
Ol-ORA4      WMWWRAVGCWVTLTLSIFHCTTLKRH-HVSL--GPLVLQKEKRRVWIILGLVWGANLAFS
Tn-ORA4      WVWWRAVGCWLTLVLSVFHCTTLKRQ-RLYI--GPNAQRRERRRLWVILGLVWGLNLAFS
Tr-ORA4      WVWWRAVGCWLTLALSIFHCTTLKRQ-RLYM--GPDMQRRERRRMWVILGLVWGLNLAFS
Gm-Ora4      WVWWRAVGCWVTLALSAFHCSTLRRQ-HVAF--GPLAVQKERRRVWGALGLVWGVNLVLS
Ga-ORA4      WVWWRAVGCWVTLALSIFHCTVLRRQ-HVAC--GPLAQERERRHVWIALGLVWGANLAFS
Dr-ORA4      WVWWRAVGCWVTLALSAFHCATLRRQ-HVSM--GPLGHSRERRRVWVVLAVVWAANLLFS
Lo-ORA4      WVWWRAVGCWATLGLSLFHWAMLRRH-SFMS--GLQAHRAELRRVCVALALVWALNFAYS
Am-ORA6      TLSGEVASIFFTVLISIYRYQKLHNA-AMRI--ITPIFMDSMKIGVGLSLLCVLVAVLAS
Dr-ORA6      TVFGEVCSVLFTALISIYRHQKLHDV-FSHV--NVPVLLDSLRWAVCMCVLCVCVALAFG
Fh-ORA6      TVFGEIASILFTVVICIFRYQKLRDI-NHRG--SLPIWLDSITSAGMMSGVCVTLSTLVS
Pf-ORA6      TTFGEIASILFTVIICIFRYQKLRDV-DHRG--SLPICLDSIASAWMLSGVCVTLSTLLS
Xm-ORA6      TTFGEIASILFTVVICIFRYQKLRDV-DHRG--SLPICLDSIASAWTMSGVCVTLSALLS
Ga-ORA6      SKFGEVASIFFTVLISVFRQQKLSDA-AKRA--NLPIYLDSIGSARMASGVCVLLATLLS
Hc-ORA6      TVIGEINSIFFTVLICIFRYQKLRDL-NTRV--NFPLFLDNIRSAWTVSGISVMLSVLLS
On-ORA6      TVIGEINSIFFTVLICIFRYQKLRDV-NTRV--NFPLFLDNIRSAWMVSGISVMLSVLLS
Tn-ORA6      TSLGETASILITVLISIFRYQKLRDA-SRRV----PIYLDSIRSAWTVSGILLMFTVLLA
Tr-ORA6      TMLGENASILVTVLISIFRYQKLRDA-SRRV--NLPIYLDSIRSVWTVSGILTVFTILLS
Gm-ORA6      NLLGEVSSILFTVLICVFRYQKLRDA-EKRG--NAPIFLDSRKSAWVVSGLCMLLSVMLG
Ol-ORA6      TMFGETASIFFTVIISIFRYQKL------GV--SLSVHPDRIGVAQLLSGVCVMFSFLLS
Lo-ORA6      ASLGETATIFFTVLISVFRYQKLRHA-EARG--NLPTSWDNTSTAWALSGMSLFLSFCFC
Ss-ORA6      LVFGKIASILFTVLFSIFRYQK--------------------------------------
Am-ORA5a     SDLSETSSIFTTMFITVFWHQKLVGS-LKHG--GAPVQMDNTRLVMALLAGSWTVSVVFS
Am-ORA5b     SDLSETSSIFTTLFITVFWHQKLVGS-LKRG--GAPVQMDNIRLVAALLAGSWIVAIAFS
Fh-ORA5      ADLSETSSLFTTLFICAFWHQKLVGS-LKRG--GAPVQLENLRLVGCLLAGSWILSTVFS
Pf-ORA5      ADLSETSSLFTTIIICVFWHQKLVGS-LKRG--GAPVQLDNLRLLGCLLVGSWTLSVVLS
Xm-ORA5      SDLSETSSLFTTLIICIFWHQKLVGS-LKRG--GAPVQLDNLHLLGCLLAGSWTLSAIFS
Gm-ORA5      ADLSETSSIFSTLFISVYWYQKLVGS-LKRG--GGPVQLDSLRLVGGLLAGSWGVAVVFS
Ga-ORA5      ADLSETSSIYSTFFISVFWHQKLVGS-LKRG--GAPVQLDRLCLVGCLLAGSWTVAAVFS
Tn-ORA5      ADLSETSSIFTTLFISVFWHQKLVGS-LRRG--GAPVQMDNLCLVACLLAGSWTVAAVFS
Tr-ORA5      ADLSETSSIFTTLFISVFWHQKLVGS-LKRG--GSPVQMDSLCLVACLLAGSWTVAVVFS
Hc-ORA5      AGLSETSSIFTTFFISVFWHQKLVGS-LKRG--GAPVQMDSLCLVGWLLAGSWTVAVVFS
On-ORA5      AGLSETSSIFTTFFISVFWHQKLVGS-LKRG--GAPVQMDSLCLVGCLLAGSWTVAVVFS
Ss-ORA5a     SDLSETSSVFSTLFISVFWYQKLVGS-LKRG--GAPVQLDSLRLVACLLVGSWTVAVVFS
Ss-ORA5b     SDLSETSSIFSTLFISVFWYQKLVGS-LKRG--GAPVQLDSLRLVAYLLAGSWTVAGVFS
Ol-ORA5      IDFSETSSLFSTFLISAFWHQKLVGS-LKRG--GAPVQLDNLCLVGFLLAGSWTVCAVFS
Dr-ORA5      SDLSETSSIFSTMFITLYWHQKLVGS-VRRG--GAPVQLDNLRLVLWLLLGSWMVALTFS
Lo-ORA5      SDLSETSSVLTTLLISVFWYQKLVGS-LKRG--NAPVKLDSLGLSCGLLAASWGAALVFS
```

```
Am-ORA1         IAAPFFSVAPR----NGTVP-----PFTLNLGFCHVDFR------DNLSYVINGAA-VSA
Fh-ORA1         VAAPLFSMAPR----NGTVP-----AFTLNLGFCHVDFR------DNLSYVVNGVA-VSG
Pf-ORA1         IAAPFFSMAPR----NGTIP-----AFTLNLGFCHVDFR------DNMSYVINGVA-VSG
Xm-ORA1         IAAPFFSMAPR----NGTIP-----AFTLNLGFCHVDFR------DNMSYVINGVA-VSG
Sc-ORA1         IAAPFFSMAPR----NGTVP-----AFTLNLGFCHVDSR------DHLSYVINGVA-VST
Sr-ORA1         IAAPFFSMAPR----NGTVP-----AFTLNLGFCHVDFR------DHLSYVINGVA-VST
Hc-ORA1         ISTPLFSMAPR----NGTVS-----AFTLNLGFCLVDFR------DNLSYVINGVA-ISG
On-ORA1         IAAPLFSMAPR----NGTVP-----AFTLNLGFCHVDFR------DNLSYVINGVA-VSG
Ol-ORA1         IAAPFFSMAPR----NGTVL-----PFTLNLGFCHVDFR------DNLSYVINGVA-VSV
Ga-ORA1         VAAPLFSMAPR----NGTAP-----AFTLNLGFCHVDFR------DNLSYVINGVA-VSV
Ss-ORA1         LAAPLFSIAPR----NGTVP-----AFTLNLGFCHVDFR------DRLSYKINGVV-VST
Dr-ORA1         IAAPFFSVAPR----NGTVP-----PFTLNLGFCHVDFH------DNLSYVLNGVA-VSV
Gm-ORA1         IAAPFFSIAPR----NGTVP-----AFTLNLGFCHVDFR------DYLSYVINGVA-VSV
Lo-ORA1         IAAPFFSIAPR----NGTVP-----EFTLNLGFCHVDFR------DSLSYIINGVA-VSG
Lo-ORA8a        QASLLYSSAPT----NSSLS-----EYTLNLEFCIVAFP------SFEAYMGNGVM-HIV
Lo-ORA8b        ----------------------------------------------------VM-YIV
Lo-ORA7         YTSILYTSQVR----NTTLS-----TNTLNLGYCLVVFP------SEESYFANGVM-YLT
Am-ORA2         APTLVFAVAARN---DSKLL-----ENSINIQFCFINFP------SVFAKDANGAL-QVV
Fh-ORA2         VGAVLFSVSSRN---YTAMT-----TSAVNAEFCYVNFR------SDVLKEAYGAI-QVS
Pf-ORA2         AGAILFSVSSKN---GTAVT-----TSAVNLEFCYVNFP------SDIIKEVYGAI-QVS
Xm-ORA2         AGAILFSVSSKN---VTAVA-----TSAVNVEFCYVNFP------SDILKKVYGAI-QVS
Gm-ORA2         SAAVLFSVGAGNGNGSSSLG-----SNGINVQFCVVRFP------TRLSKDANGAV-QVA
Ga-ORA2         SAGILFSFGTKN---VTNLT-----NFDINVQFCYVNFP------SKLSIQANGAS-QVG
Hc-ORA2         TAAILFSFSTKN---ETSPI-----DNGINVQFCYLNFP------SKLSRDANGAI-QVG
On-ORA2         TAAILFSFSTKN---ETSPI-----DNGINVQFCYVNFP------SMLSRDANGAV-QVG
Ss-ORA2         SAAIAFSLGSRN---DSVNM-----QHSINVQFCYVRFP------TMQSKQVNGAV-QVG
Ol-ORA2         SGAILFSLSSKN---DSSPI-----NNAVNLEFCFVSFP------SDLSKEIFGAV-QVS
Tn-ORA2         TAAILFSFSTQN---GTNLV-----NNSINVQFCYVQFP------SKLSKDANGAA-QVG
Tr-ORA2         SAAVLFSFGTQN---DTNLI-----NHSVNVQFCFVHFP------SKMSRDANGAA-QVG
Dr-ORA2         VASLLYSIGARN---DSRLL-----QNAINVEFCFLSFP------SRLARDANGAA-QVA
Lo-ORA2         SAALAFAVSSGD---NSTRT-----QYGINIEFCIVNFP------SSTWKNAVGAV-QVA
Am-ORA3         VPAFIYSTSGGK---NATETLMLVSSTTRPLLGCLWDFP------SVYSGLAFATTSMVI
Fh-ORA3         IPAHIFSTSGNE---NTTETLMLVSSTTRPLLGCVWNFP------SNYSGLAYATTSMAI
Pf-ORA3         IPAHIFSTSGNE---NTTETLMLVSSTTRPLLGCVWNFP------SNYSGLAYATTSMAI
Xm-ORA3         IPAHIFSTSGNE---NTTETLMLVSSTTRPLLGCVWNFP------SNYSGLAYATTSMMV
Ga-ORA3         IPAHIFSTSGDV---NSTETLMLVSSTTRPLLGCVWNFP------SSYSGLAYATTSMVI
Tn-ORA3         IPAHIFSTSGNI---NSTETLMLVSSTTRPLLGCIWNFP------SRYSGLAYATTSMVL
Tr-ORA3         IPAHVFSTSGNI---NSTETLMLVSSTTRPLLGCIWNFP------TRFSGLAYATTSMVL
Ol-ORA3a        IPAHIFSTNGNA---NSTETLMLISSTTRPLLGCVWNFP------SSYSGLAYATTSMVI
Ol-ORA3b        IPAHIFSTNGNA---NSTETLMLISTTTRPLLGCVWNFP------SSHSGLAYATTSMVI
Gm-ORA3         IPAHVFSINGNQ---NSTETLMLVSSTTRPLLGCIWNFP------LK-NGLLYATISMVI
Hc-ORA3         IPAHIFSSNGNK---NTTETLMLVSSTTRPLLGCVWNFP------ST-IGLAYATTSLVI
On-ORA3         IPAHIFSSNGNK---NATETLMLVSSTTRPLLGCVWNFP------ST-SGLAYATTSLVI
Ss-ORA3a        VPAHIYSTKGNK---NST------------------------------------------
Ss-ORA3b        VPAHIYSTKGNK---NSTETLMLVSSTTRPLLGCVWNFP------SSYDTLAYTTTSMVI
Dr-ORA3a        IPAFIFSKNGNE---NSTETLMLVSSTTRPLLGCIWDFP------SAYSGLAFATSSMIL
Dr-ORA3b        IPAFIFSKNGDA---NSTETLMLVSSTTRPLLGCIWNFP------SAYSGLAFATSSMIL
Lo-ORA3         FPGFFFSTQGGR---NSTEELMLVSSTTRPLLGCVWSFP------SRRGGLAYATTSLVL
Am-ORA4         IPASVFTSHVHG---NATTEVMVISCTTRPLLGCMWNFP------TREQGYAFAAASMAL
Fh-ORA4         VPALLYTTHVQS---NATVELMVISCTTRPLLGCVWEFP------SSQQGSAFASASLAL
Pf-ORA4         IPALLFTTHIES---NATMELMVISCTTRPLLGCVWKFP------SDRQGLAFASTSLAL
Xm-ORA4         IPALLFTTRVES---NATVELMVISCTTRPLLGCAWKFP------SDQQGSAFASTSLAL
Hc-ORA4         VPALVYSTHVKG---NATVELMVISSTTRPLLGCVWEFP------SEEQGTVFASTSLAV
On-ORA4         VPALVYSTHVKG---NATVELMVISSTTRPLLGCVWEFP------SEEQGLVFTSTSLAV
Ss-ORA4         LPALVYTTHVQG---NATVELMVISCTTRPLLGCMWEFP------SEEQGSAFASTSLAL
Ol-ORA4         IPALVYSTHVYS---NATVDLMVISSTTRPLLGCIWEFP------TTQQGSAFAFTSLAL
Tn-ORA4         TPALIYSTHVHG---NATVELMVISCTTRPLLGCIWEFP------TAQQGSAFASASLAL
Tr-ORA4         IPALIYSTHVHG---NATVELMVISCTTRPLLGCIWEFP------TAQQGSAFASASLAL
Gm-Ora4         LPALVYTTHVHG---NATVELMVISCTTRPLLGCIWEFP------SREQGEAFASTSLVL
Ga-ORA4         IPALVYSTHVHG---NATVELMVISCTTRPLLGCVWEFP------SNQQGSAFASASLAL
Dr-ORA4         LPALVYTTQVRG---NATVELMVISCTTRPLLGCVWEFP------TFQQGYAFASSSLAL
Lo-ORA4         LPALVYSTHSRG---NTTVELMVISCTTRPLLGCVWEFP------SEVQGIAFATASLVV
Am-ORA6         VPTYIINLDSWHHMYNSTIT------------DCPADFFQCPRDNCPILNNIYRFLFIFF
Dr-ORA6         LPTLLVNTHWSVS--NSSLE------------RCPVDFFQCPSS-SPCLTHIYKYVFLLV
Fh-ORA6         LPVFFTLQESVK---NTTVNSG----------GCPSDLFQCGENYCPTLNRVYKYLMMIL
Pf-ORA6         LPMFAVTFQGSVE--NVTENRG----------GCPTDFFQCGENYCPIFNRVYKYLIMLL
Xm-ORA6         LPMFAIAFRGSVE--NVTENRE----------GCPTDFFQCGENYCPILNCVYKYLIMLL
Ga-ORA6         LPVFAIEPKEPAG--NATGNATG--NATGNAIGCPPDFFQCSKSRCPALNGLYKHVFILV
Hc-ORA6         VPMFVIDQESKAE--NVTRNSS----------MCPPDFFHCTQNHCPVFNRIYKYLFIVS
On-ORA6         VPMFVIDQESKAE--NVTRNSS----------VCPPDFFHCTQNHCPVFNSIYKYLFIVL
Tn-ORA6         SPIFVLNIKEMSQ--NVTINGS----------GCPPDFFQCNKDNCPELNGIYKYLFILL
Tr-ORA6         SPIFVLNIKETSQ--NFTNNGS----------GCPPDFFQCNKEDCPELNGIYKYLFILL
Gm-ORA6         LPVYFVRIDTHVE----ADNGT----------SCSPDFFQCHEHFCPPLNRFYKYLFLVS
Ol-ORA6         FPVVAIKPAALTE--SAANNSGG---------GCPADSFHCGKNYCPAPNRAYKYLFILV
Lo-ORA6         LPGYFIESDERVD--NHTRSR-----------NFLTDPFQCPRINCPAINLIYKTLFLLF
Ss-ORA6         ------------------------------------------------------------
Am-ORA5a        LPHLFFTSLNIQ---NQSSE------------ECLEYFP------SQEVKQTYEMVFLML
Am-ORA5b        LPHIFLASKNNG---NNTYF------------ECLEDYP------SLKAKQAYDLMYLVF
Fh-ORA5         IPHFFFVSLEGT---NGSRE------------DCIDVFP------DALSSQIYEIFFLVL
Pf-ORA5         MPRLFFVSLEVT---NESLE------------HCVDVFP------NVLSRQTYEVFFLSL
Xm-ORA5         MPRLFFVSQEVK---NESHE------------NCIDVFP------DVLSRETYEVIFLSL
Gm-ORA5         IPHYFFVTVEGE---NGSSL------------ECNDVFP------SEEAKQTYEALYLTL
Ga-ORA5         IPHVFFVAVEGR---NGSKV------------DCVDVFP------SAVARQTYEIFYLTL
Tn-ORA5         VPHFFFVKVEAT---NESSE------------DCIDVFP------NKLAKQTYEIIYLTL
Tr-ORA5         IPHFFFVKVEGS---NESSE------------DCIDVFP------NKAAKQTYEIIYLTL
Hc-ORA5         VPHYFFFTLEVA---NDSHE------------DCIEIFP------NPNARQIYEAIYLTL
On-ORA5         VPHFFFFTLEGA---NDSHE------------DCIEIFP------NPNARQTYEAIYLTL
Ss-ORA5a        VPQLFFVRMESG---NESHD------------DCIEIFP------SQTARQTYEPLYLTF
Ss-ORA5b        VPHFFFVQVDSG---NESHK------------DCIEVFP------SQTARRTYETLYLTL
Ol-ORA5         IHHFFLASVEGI---NGSHR------------YCVDAFP------SALAEQTFDIIFLTV
Dr-ORA5         VPHFFIAEHDG----NDTLE------------VCEEKFP------TPAEKKTFDGLYLIV
Lo-ORA5         VPLLSFVTVGSN---RSASQ------------DCQAHFP------THASKQTYEATYLTL
```

```
Am-ORA1      RDFSFVGLMLGSSGYILVLLHKHS--RQVRAIRR--------------------------
Fh-ORA1      RDFAFVALMVGSSGYILLLLHRHS--EQVRGIRR--------------------------
Pf-ORA1      RDFAFVALMLGSSGYILLLLHRHS--EQVKGIRR--------------------------
Xm-ORA1      RDFAFVALMLGSSGYILLLLHRHS--EQVKGIRR--------------------------
Sc-ORA1      RDFAFVALMLGSSGYILLLLHRHS--RQVRGIRR--------------------------
Sr-ORA1      RDFAFVALMLGSSGYILLLLHRHS--RQVRGIRR--------------------------
Hc-ORA1      RDFAFVALMVGSSCYILLLLHRHS--HQMKGIHR--------------------------
On-ORA1      RDFAFVALMVGSSCYILLLLHRHS--HQVKGIRR--------------------------
Ol-ORA1      RDFAFVALMVGSSGYILLLLHRHS--HQVRKIRR--------------------------
Ga-ORA1      RDFAFVALMLGSSGYILLLLHRHS--RRVRGIRR--------------------------
Ss-ORA1      RDFAFVGLMLWSSGYILLLLHRHS--HQVRSIRRS-------------------------
Dr-ORA1      RDFAFVGAMLASSGFILLLLHRHR--RQVRAVRR--------------------------
Gm-ORA1      RDFAFVLLMLLSSGYILLILHRHS--RQVRSMRR--------------------------
Lo-ORA1      RDFIFVGLMVCSSGYILLLLHRHG--KQVRQIRSPDQH----------------------
Lo-ORA8a     RDFVFVGMMASAGGYIVVILYRHR--KQTRGLQGA-------------------------
Lo-ORA8b     RDFVFVGMMASAGGYIVVILYRHR--RQTRGLQGA-------------------------
Lo-ORA7      RDLFFVILMVLASFYILLLLYRHQ--RRVKGLQSSNM-----------------------
Am-ORA2      RDVIPMSLMTTASFIILVFLYRHS--RQVSNLRSGTGTG---------------------
Fh-ORA2      RDVVPMALMTLTSLVILVLLYKHS--QHLKGLRGAGSAG---------------------
Pf-ORA2      RDVVPMALMTLTSLIILVLLYKHS--QHLKGLRGAGHAG---------------------
Xm-ORA2      RDVVPMALMTLTSLIILVLLYKHS--QHLKGLRGAGHAG---------------------
Gm-ORA2      RDVVPMGLMATASLVILVFLYRHS--RQVKGLRSGGG-----------------------
Ga-ORA2      RDVVPMALMTLDSLIILVFLYKHS--QQAKDLRGSRG-----------------------
Hc-ORA2      RDVVPMALMTLASLIILVFLYKHS--QQVKGLRSSGGGG---------------------
On-ORA2      RDVVPMALMTLASLIILVFLYKHS--QQVKGLRSSGGGG---------------------
Ss-ORA2      RDVVPMGMMTLASLVILVFLYRYS--QQVKGLRSSSG-----------------------
Ol-ORA2      RDVVPMALMTLTSVIILVLLYQHN--QHMNDLHRNSNAS---------------------
Tn-ORA2      RDVVPMTLMTLASLIILAFLYKNS--QQVKGLRSS-------------------------
Tr-ORA2      RDVVPMALMTLASLIILAFLYKNS--QQVKGLRSRDG-----------------------
Dr-ORA2      RDVVPMVLMAAGSLVLLVYLVRQR--RRVQGLRG--------------------------
Lo-ORA2      RDAVPIFLMVAASLFILLFLYRHS--QQVKGLRSAKR-----------------------
Am-ORA3      HEIIPIVLMSVTNLGSLLTLYAHG--SKLHAT----------------------------
Fh-ORA3      HEIFPIILMAVTNMTSLYTLYTHG--RSRSS-----------------------------
Pf-ORA3      HEIFPIILMAVTNMTSLYTLYTHG--RSRSS-----------------------------
Xm-ORA3      HEIFPIILMVVTNMMSLYILNTYG--RSRGS-----------------------------
Ga-ORA3      HETIPIILMAITNLGSLYTLYTHS--RVRST-----------------------------
Tn-ORA3      HETVPIVLMALTNLGSLYTLYTHN--GMRSS-----------------------------
Tr-ORA3      HETVPIVLMALTNLGSLYTLYTHN--GMQSS-----------------------------
Ol-ORA3a     HETLPIVLMTVTNLSSLYTLHTYG--RTRKS-----------------------------
Ol-ORA3b     HEALPILLMTATNLSSLYMLHTYSRTRTRTS-----------------------------
Gm-ORA3      HETLPIVLMAFTNAGSLYSLYAHS--KMRSM-----------------------------
Hc-ORA3      HEMIPIILMAITNLTSLYTLYTHG--RNP-------------------------------
On-ORA3      HEMIPIILMAVTNLTSLYTLYTHG--RNP-------------------------------
Ss-ORA3a     -EILPIILMAITNLGSLYTLYTHG--RTHNPA----------------------------
Ss-ORA3b     HEILPVILMAITNLGSLYTLYTHG--RTRNPA----------------------------
Dr-ORA3a     HESIPICLMNITNLGSLCTLYAHG--HKRTVA----------------------------
Dr-ORA3b     HESIPICLMSITNMGSLLALYAHG--EARRAA----------------------------
Lo-ORA3      HELLPILLMVATNLGTLHTLARHG--RSQRA-----------------------------
Am-ORA4      NEVLPLVLMVGTNLATLHTLAKHI--RAVAAGPEMA------------------------
Fh-ORA4      NEVLPLVLMVCTNLATLHSLAKHI--RAVTD-----------------------------
Pf-ORA4      NEILPLVVMVFTNLATLHSLAKHI--RAVTS-----------------------------
Xm-ORA4      NEVLPLVVMVFTNLLILHSLVKHI--RAVTS-----------------------------
Hc-ORA4      NEVLPLVLMVCTNVATLHALAKHI--RAVAS-----------------------------
On-ORA4      NEVLPLVLMVCTNVATLHALAKHI--RAVAS-----------------------------
Ss-ORA4      NEVVPLVLMVGTNLATLHSLAKHI--RAVTSAGEA-------------------------
Ol-ORA4      NEVFPLVLMICTNVSTLHALAKHI--RAVTSSME--------------------------
Tn-ORA4      NEVLPLVLMVCTNLATLHALAKHI--RAVVSS-Q--------------------------
Tr-ORA4      NEVLPLVLMVCTNLATLHALAKHI--RAVMSSGQ--------------------------
Gm-Ora4      NEVLPLVLMICTNLATLHALAKHI--RAVTA-----------------------------
Ga-ORA4      NEVLPLVLMVCTNLATLHALAKHI--RAVAAGAH--------------------------
Dr-ORA4      NEVLPLVLMVGTNLATLQALGKHI--RTVRA-----------------------------
Lo-ORA4      NELVPLVLMVGTNLASLCVLRRHI--HTVAG-----------------------------
Am-ORA6      CYLIPLVIVTGTSSLIIRILMIQQ--KVAELHHNSEPATIAANND--------HHHHHHN
Dr-ORA6      CVVLPLLVVTVTSVLMVRVLLAQQ--RAVRVREA--------------------------
Fh-ORA6      CHLLPLIIVTVTSCLTITVLLRQI--YSVTPANDAR------------------------
Pf-ORA6      CHLLPLIIVTVTSCLTIVVLLGRT--NTVTPANDII------------------------
Xm-ORA6      CHLLPLIIVTVTSCLTIVVLLGRT--NTVTPANDII------------------------
Ga-ORA6      CNLLPLAVVTVTGCLILAVLLGQR--STVTPA----------------------------
Hc-ORA6      CHLLPLIIVTVTSCLILTVLLSQR--KTVTPAVNET------------------------
On-ORA6      CHLLPLIIVTVTSCLILAVLLSQR--KTVTPAVNET------------------------
Tn-ORA6      FNLLPLIIVTVTSCLIIMVLLSQR--KTVAPVVNAS------------------------
Tr-ORA6      FNLLPLIIVTVTSCLIIAVLLSQR--KTVTPVESGS------------------------
Gm-ORA6      CNLLPLLAVTVSSSLIVKVLLGQK--RVVAPALGAS------------------------
Ol-ORA6      SYLLPLIVITVTNCLILAVLLVQR--RTITPEISVH------------------------
Lo-ORA6      SNLIPLLIITATSGLILKVLLHRR--KTVSDVYD--------------------------
Ss-ORA6      ------------------------------------------------------------
Am-ORA5a     ANVVPIVGIVFASIQITVTLLQSQ--KRIKNISSRAGPRGDD-----------QRKAASN
Am-ORA5b     ANIIPIIGIFFASIQITVTLLQNQ--KRINSNTTAVTTGAK------------NTKITPA
Fh-ORA5      ANALPVAGIMVASVQIVVTLLQNQ--KRIQSRSSDPAKGAIKEN---------HPERSVS
Pf-ORA5      ANALPIAGIWVASIQIVLALLQHH--KRIQSASSHRVNVKQD-----------KSERSVS
Xm-ORA5      ANALPVAGIWVASIQIVLTLLQHR--KRIQSVSSHRVKVKQD-----------KSERSVS
Gm-ORA5      ANALPVAGIVYATARIVVTLMQSQ--KRIQGHGGNQAASEE------------GRAAPAA
Ga-ORA5      ANALPLAGLVFASAQIVVTLLRNK--QRVQGHSSGASEEGENKSGGG------RDGGVAG
Tn-ORA5      ANIFPVAGIVFASMQIVVTLLQNQ--RRIQSHSSNPTQTTN------------RTEDRSS
Tr-ORA5      ANAVPVAGIVFASVQIVITLLRNH--RRIRSHGPDPTKISN------------EPKDRSS
Hc-ORA5      ANALPMAGIVFASAQIVITLLQNH--QRIRSHNSDQTKEMVKEERKRSESK--RNKASVS
On-ORA5      ANALPMAGIVFASAQIVITLLQNH--QRIRSHNSDQTKEMVKEERKRFESK--RNKASVS
Ss-ORA5a     ANALPIAGIAFASIQIVITLLRNQ--TRIQGLTSDHHKGTANSLPNN-------------
Ss-ORA5b     ANALPIAGIVFSSIQIVITLLRNQ--MRIKGLTSDHHKGTDKALPNKREKMDVSEKYDET
Ol-ORA5      ANVFPLVGIIVASFQIVVTLLQSQ--KRIGGHTSVSPKEMIRED---------KSSQSKK
Dr-ORA5      ANVVPLVGITYASVQIVVTLIQSQ--KRVKDHSGGSE-----------------------
Lo-ORA5      ANAVPVAFMVFTNLQIVITLLTQR--KRIEALKKEARLQP-------------AAERAQP
```

```
Am-ORA1         ------------------------------------------------------------
Fh-ORA1         ------------------------------------------------------------
Pf-ORA1         ------------------------------------------------------------
Xm-ORA1         ------------------------------------------------------------
Sc-ORA1         ------------------------------------------------------------
Sr-ORA1         ------------------------------------------------------------
Hc-ORA1         ------------------------------------------------------------
On-ORA1         ------------------------------------------------------------
Ol-ORA1         ------------------------------------------------------------
Ga-ORA1         ------------------------------------------------------------
Ss-ORA1         ------------------------------------------------------------
Dr-ORA1         ------------------------------------------------------------
Gm-ORA1         ------------------------------------------------------------
Lo-ORA1         ------------------------------------------------------------
Lo-ORA8a        ------------------------------------------------------------
Lo-ORA8b        ------------------------------------------------------------
Lo-ORA7         ------------------------------------------------------------
Am-ORA2         ------------------------------------------------------------
Fh-ORA2         ------------------------------------------------------------
Pf-ORA2         ------------------------------------------------------------
Xm-ORA2         ------------------------------------------------------------
Gm-ORA2         ------------------------------------------------------------
Ga-ORA2         ------------------------------------------------------------
Hc-ORA2         ------------------------------------------------------------
On-ORA2         ------------------------------------------------------------
Ss-ORA2         ------------------------------------------------------------
Ol-ORA2         ------------------------------------------------------------
Tn-ORA2         ------------------------------------------------------------
Tr-ORA2         ------------------------------------------------------------
Dr-ORA2         ------------------------------------------------------------
Lo-ORA2         ------------------------------------------------------------
Am-ORA3         ------------------------------------------------------------
Fh-ORA3         ------------------------------------------------------------
Pf-ORA3         ------------------------------------------------------------
Xm-ORA3         ------------------------------------------------------------
Ga-ORA3         ------------------------------------------------------------
Tn-ORA3         ------------------------------------------------------------
Tr-ORA3         ------------------------------------------------------------
Ol-ORA3a        ------------------------------------------------------------
Ol-ORA3b        ------------------------------------------------------------
Gm-ORA3         ------------------------------------------------------------
Hc-ORA3         ------------------------------------------------------------
On-ORA3         ------------------------------------------------------------
Ss-ORA3a        ------------------------------------------------------------
Ss-ORA3b        ------------------------------------------------------------
Dr-ORA3a        ------------------------------------------------------------
Dr-ORA3b        ------------------------------------------------------------
Lo-ORA3         ------------------------------------------------------------
Am-ORA4         ------------------------------------------------------------
Fh-ORA4         ------------------------------------------------------------
Pf-ORA4         ------------------------------------------------------------
Xm-ORA4         ------------------------------------------------------------
Hc-ORA4         ------------------------------------------------------------
On-ORA4         ------------------------------------------------------------
Ss-ORA4         ------------------------------------------------------------
Ol-ORA4         ------------------------------------------------------------
Tn-ORA4         ------------------------------------------------------------
Tr-ORA4         ------------------------------------------------------------
Gm-Ora4         ------------------------------------------------------------
Ga-ORA4         ------------------------------------------------------------
Dr-ORA4         ------------------------------------------------------------
Lo-ORA4         ------------------------------------------------------------
Am-ORA6         HHHHHDN-----------------------------------------------------
Dr-ORA6         ------------------------------------------------------------
Fh-ORA6         ------------------------------------------------------------
Pf-ORA6         ------------------------------------------------------------
Xm-ORA6         ------------------------------------------------------------
Ga-ORA6         ------------------------------------------------------------
Hc-ORA6         ------------------------------------------------------------
On-ORA6         ------------------------------------------------------------
Tn-ORA6         ------------------------------------------------------------
Tr-ORA6         ------------------------------------------------------------
Gm-ORA6         ------------------------------------------------------------
Ol-ORA6         ------------------------------------------------------------
Lo-ORA6         ------------------------------------------------------------
Ss-ORA6         ------------------------------------------------------------
Am-ORA5a        ELSSKDYISNATSANAPN---TIQK-----------------------------------
Am-ORA5b        EPS-------------------------------------------------VHSNQSSQ
Fh-ORA5         SVSVPDHCKDLKGPSSPA--------------------------------------HSGK
Pf-ORA5         SVSVQDSHKDLKESSSPA--------------ERVSSCP--------NGECLGKSPQSSP
Xm-ORA5         SVSVQDSHKDLKESSSPT--------------EHVSSCP--------NGKCLGKSPRSSP
Gm-ORA5         AAS-------------------------------------------------IKAAGGAE
Ga-ORA5         TASGPGPTEDPKDPSSLT--------------DIYTGVPASARP---TGGSPGHA---GT
Tn-ORA5         CTSG-------KDSTLTS--------------PVYTGVP----PVSADGGALAQSSGEPQ
Tr-ORA5         D----------PGPTPPS--------------QVYTGVP----P---SGGAVAQSSREPQ
Hc-ORA5         IISGPTTSKDLRDSTSTN--------------HIYTGVPAPSSP---NRQPSGHSLHNAP
On-ORA5         IISGPTSSKDLRDSTSTN--------------HIYTGVPAPSSP---NRQPSGHSLRNAP
Ss-ORA5a        ------------------------------------------------GPSVVHSSISSP
Ss-ORA5b        GIKTSDKEADLPIVASPDFPQSCRNQRISDIRPNLPGVSCSVLPC--AGPSVVHSSISSP
Ol-ORA5         QVMGPLKGLKVVRTYKSE--------------PVTSSVT------------LPQTSSQNK
Dr-ORA5         ------------------------------------------------------------
Lo-ORA5         TSTGPDSPREFSSVSTPA----------------------------------GNQNPSSQ
```

```
Am-ORA1     -------------------------------SQGGSMEMRAAKTVVMLVVLYAVFFGIDN
Fh-ORA1     -------------------------------SHGSGAETRAAKTVVTLVVLYVVFFGIDN
Pf-ORA1     -------------------------------SHGSRAETRAAKTVVILVVLYVVFFGIDN
Xm-ORA1     -------------------------------SHGSRSETRAAKIVVTLVVLYVVFFGIDN
Sc-ORA1     -------------------------------SQGGGAETRAAKTVITLVVLYVVFFGIDN
Sr-ORA1     -------------------------------SQGGGAETRAAKTVITLVVLYVVFFGIDN
Hc-ORA1     -------------------------------SQGGGAETRAAKAVLTLVVLYVVFFGIEN
On-ORA1     -------------------------------SQGGGAETRAAKTVLTLVVLYVVFFGIDN
Ol-ORA1     -------------------------------SHSSGAETRAAKTVLILVILYVVFFGIDN
Ga-ORA1     -------------------------------SQGGGAETRAAKTVITLVVLYAVXFGIDN
Ss-ORA1     -------------------------------SQGGGAETRAAKTVITLVVLYAVFFGIDN
Dr-ORA1     -------------------------------SQGSTMETRAARTVLMLVILYSVFFGIDN
Gm-ORA1     -------------------------------GAAQGVETRAAKTVVTLVVLYAVFFGIDN
Lo-ORA1     -------------------------------SSRNAAETRAAKTVVTLVTLYVVFFGIDN
Lo-ORA8a    -------------------------------ARMQRKTVEASKAVLTLIAMYVILFGLDN
Lo-ORA8b    -------------------------------DRMQRKTVEASKAVLTLIAMYVILFSLDN
Lo-ORA7     -------------------------------SQGSRAETRAAKTVVTLVTLYVLFFGIDN
Am-ORA2     -------------------------------GGGASAERRAAISVVVLVTFYVLMYGVDN
Fh-ORA2     -------------------------------AGSCGAEQRAAKVVVVLVTVYVVLYGVDN
Pf-ORA2     -------------------------------SGTCGSKQRAAKVVVVLVTIYVVLYGVDN
Xm-ORA2     -------------------------------SGTGGSKQRAAKVVVVLVTIYVVLYGVDN
Gm-ORA2     -------------------------------GGRDGAERRAAKAVVALVTLYVGLYGVDN
Ga-ORA2     ----------------------------------GGAERRAAKVVVALVTLYVVLYGVDN
Hc-ORA2     -------------------------------AGNSGAEQRAAKAVVALVTLYVVFYGADN
On-ORA2     -------------------------------AGNGGAEQRAAKAVVALVTLYVVFYGVDN
Ss-ORA2     --------------------------------ASGGAERRAAKAVVVLVTLYVVLYGVDN
Ol-ORA2     -------------------------------GGRCGAKRRAAKVVVVLVTLYLGLYGVDC
Tn-ORA2     --------------------------------SSDRAEKRAAKAVVTLVSLYVLLYGVDN
Tr-ORA2     --------------------------------GSGRAERRAAKAVVTLVTLYVLLYGVDN
Dr-ORA2     -------------------------------TAGGAAERRAAVTVVTLVSLYLLVFGLDN
Lo-ORA2     -------------------------------TQKESAESRAAKTVVTLVTLYVLFYGIDN
Am-ORA3     ---------------------NKSQQDPT--MNRVPAERRAAKVILALILLFIVS-----
Fh-ORA3     -----------------------VQDAPV--IKRVPAERRAAKVILALVMLFIVS-----
Pf-ORA3     -----------------------VQDAPV--LKRVPAERRAAKVILALVLLFIVS-----
Xm-ORA3     -----------------------VQDVPV--LKRVPAEKRAAKVILALVMLFIVS-----
Ga-ORA3     -------------------------DAPV--IKRVPAERRAAKVILTLIMLFIVS-----
Tn-ORA3     -----------------------VQEVPI--KKRVPAERRAAKVILALIMLFIIS-----
Tr-ORA3     -----------------------VQDAPV--IKRVPAERRAAKVILALIMLFIAS-----
Ol-ORA3a    -----------------------VQDAPV--VKRVPAEKRAAKVILILVLLFTVS-----
Ol-ORA3b    -----------------------IQHAPV--IRGVPAERRAARVILILVLLFVVS-----
Gm-ORA3     -----------------------VNDVHV--IKKVPAERRAAKVILALIMLFIAS-----
Hc-ORA3     -----------------------QKDAPV--LKRVPAEKRAAKVILTLILLFILS-----
On-ORA3     -----------------------RKDATV--LKRVPAEKRAAKVILTLIILFILS-----
Ss-ORA3a    ---------------------HMTQDAPV--IKRIPAERRAAKVILALIVLFIGS-----
Ss-ORA3b    ---------------------HMTQDAPV--IKRIPAERRAAKVILALTILFIVS-----
Dr-ORA3a    ---------------------SQGEDAPV--VSRIPAERRAAKVILALNILFISS-----
Dr-ORA3b    ---------------------KKSSDAPV--VSRIPAERRAAKVILALNILFILS-----
Lo-ORA3     ------------------------GETTL--TRRIPAERRAAKVVLVLIMLFIIS-----
Am-ORA4     -------------------------------SGHSNSEKKAGHVIMALVTLFVVC-----
Fh-ORA4     ----------------------SGSHGEL--DKHVSTERKAAHVIMSLVSLFVVC-----
Pf-ORA4     ---------------------ESGSHGEL--DKHVSTERKAAHVIMCLVSLFVVC-----
Xm-ORA4     ---------------------ESGSHGEL--DKQVSTERKAAHVIVSLVSLFVIC-----
Hc-ORA4     ----------------------GGTHTEL--DKHLSSERKAAQVIMSLVLLFVVC-----
On-ORA4     ----------------------GGIHTEL--DKHLSSERKAARVIISLVLLFVVC-----
Ss-ORA4     ---------------------GGGTHGEL--DRHVASERKASHVIMLLVMLFVVC-----
Ol-ORA4     ---------------------SGGSHGEV--NKLVSTERKAAHVIMLLVALFVVC-----
Tn-ORA4     ---------------------SGGSQGEL--DKHLSTERKAAHLIMLLVSLFVTC-----
Tr-ORA4     ---------------------PGGSHVEL--DKHLSTERKAAQVIMLLVSLFVVC-----
Gm-Ora4     ---------------------AGGDSGDM--DRHLASERKAGHVIMSLVSLFVVC-----
Ga-ORA4     ---------------------PGETQKEL--DKHVSTERKAAHVIVSLVSLFVVC-----
Dr-ORA4     ---------------------GGSTGAEL--DRHVSSERKAGHVIMALVALFVGC-----
Lo-ORA4     ------------------------ANMEL--QGHMASERRASHVILVLVTLFVTC-----
Am-ORA6     --------------------HHHHHDHHH--HDHTNVFHRSTIGILAAMMIFQVY-----
Dr-ORA6     ------------------------EEPHP--HHHHSSLLRSTLAILAAMLLFLLD-----
Fh-ORA6     ------------------------CPDHP--SGKSHGLQRTACTQVGRCEHGGVA-----
Pf-ORA6     ------------------------SPNHH--HGKSHSLFRSTVAVVAAMGLFQVD-----
Xm-ORA6     ------------------------SPDHH--PGKSHGFYRSTVAVVAAMGLFQVD-----
Ga-ORA6     -------------------------SVGS--RSGGSTLRRSSVAVLAAMGLFQVE-----
Hc-ORA6     ----------------------GSSQFSR--KSKDTKIQWSTIAVLGAMGLFQVD-----
On-ORA6     ----------------------GSSQFSR--KSKDTKIQWSTIAVLGAMGLFQVD-----
Tn-ORA6     ------------------------SQTAQ--RSKCQKFQRSTIAVLTAMGLFQVD-----
Tr-ORA6     ------------------------SQISR--KSKGLKFQRSTIAVLTAMGLFQVD-----
Gm-ORA6     ------------------------GPPGK--KSKGPRLQRSTVGILTAMGVFQID-----
Ol-ORA6     ------------------------QSNHSHTNGRDLRFQHSMMAVVAAMGLFLVN-----
Lo-ORA6     -------------------------SSHH--HHQNLYFSKSTKTVLAAMCIFQLD-----
Ss-ORA6     ------------------------------------------------------------
Am-ORA5a    -------------------VQKSQDRSN---SSSGSSQVRAAKSVVAVATVFVIC-----
Am-ORA5b    -----------EAVSYSNAVVPNPGQARS--SSSSGSLLRAAKSVVVTATIFLIC-----
Fh-ORA5     TGHK--ADSGVEAQANSS--RPSQMPSKQ--STSAHSQVRAAKSVVAVATVVLIC-----
Pf-ORA5     LANK--TDSRAEAQPNHS--KSNQIPSKQ--SNNTSSQVRAAKSVVAVATVVLLC-----
Xm-ORA5     LANK--TDSRAEAQPNHSVCQSSQIPSKQ--SNNASSQVRAAKSVVAVATVVLLC-----
Gm-ORA5     GGGV--------AGGGEV--RGAKPAPKP--SPGSSNQVRAAKSVVAVASIFVVC-----
Ga-ORA5     LVGD--TYSGGGAPEGPG--RPSQTRA----KTSSGTQVRAAKSVVAVAAVFLVC-----
Tn-ORA5     PGGT------ARSHQECP--RPGQAPTKP--TLASGSQVRAAKSVVAVASVFLVC-----
Tr-ORA5     PGGT------DRTPEGGP--RPSQAPAKP--TMASSSQVRAAKSVVGVASVFLVC-----
Hc-ORA5     ------QNCSVGAQPNLS--RPSQIPSKP--HPNSSTQVRAAKSVVAVASVVLVC-----
On-ORA5     ------QNCSVGAQPNLS--RPSQIPSKP--HPNSSTQVRAAKSVVAVASVVLVC-----
Ss-ORA5a    LYQVDIGDNIVRAPADLK--RSSQPPAKP--SPGSGTQVRAAKSVVAVATVFVVC-----
Ss-ORA5b    LYQVEVGDCRVRASPDLQ--RTSQPPAKL--SPGSGTQVRAAKSVVAVATVFVVC-----
Ol-ORA5     TTGS---NCSLGAPANHS------KPSKA--NPNSSTQVRAAKSVVAVGSVVLVC-----
Dr-ORA5     --------------------QKTEVRSSG--NPSSGTHVRAAKSVVAVASIFIFC-----
Lo-ORA5     ALADPAGKGRLDALARAAAVAPPRSRNAP--RQHPGAQVRAAMSVVAVASVFLVC-----
```

```
Am-ORA1      VIWIYMLTVAQV--------PAVVADMRVFFSSCYATLSPFLMISSNKKLKERM---VC-
Fh-ORA1      VIWIYMLTVSKV--------SPVVADMRVFFSSCYASLSPYFIISSNKKVKRKI---VC-
Pf-ORA1      VIWIYMLTVSKV--------SPVVADMRVFFSSCYASLSPFFIISSNKKVKRKI---VC-
Xm-ORA1      VIWIYMLTVSKV--------SPVVADMRVFFSSCYASLSPYFIISSNKKVKRKI---VC-
Sc-ORA1      VIWIYMLTVPQV--------P---------------------------------------
Sr-ORA1      VIWIYMLTVPQV------------------------------------------------
Hc-ORA1      VIWIYMLTVEKV--------SPVVADMRVFFSSCYASLSPYFIISSNKKVKAKI---VC-
On-ORA1      VIWIYMLTVAKV--------SPVVADMRVFFSSCYASLSPYFIISSNKKVKAKI---VC-
Ol-ORA1      VIWIYMLTVSNV--------SPVVADMRVFFSSCYASLSPYFIISSNKKVKRKI---VC-
Ga-ORA1      AIWIYMLTVAKV--------SPVVADMRVFFSSCYASLSPYFIISSNKKVKAKI---LC-
Ss-ORA1      IIWVYMLTVDKV--------SPVVNDMRVFFSCCYACLSPFFIISSNKKVKSKL---VC-
Dr-ORA1      VIWIYMLTVAQV--------PPVVAHMRVFFSSCYASLSPFLIISSNRKLKARM---VC-
Gm-ORA1      VIWIYMLTVAKV--------SPVVADMRVFFSSSYAFLSPYFIISSNKRIKGKL---VC-
Lo-ORA1      IIWIYMLTVAQV--------PPVIADMRVFFSSCYASLSPFLMITSNKKIKNKL---SC-
Lo-ORA8a     VVWIYTLCVSRV--------HPIASDTRVFFASCYSALSPIFIITTNKKIVASL---SC-
Lo-ORA8b     VMWIYPLCVSHV--------HPIVSDIRVFFASCYSALSPVFIITTNKKIAASL---SC-
Lo-ORA7      LIWAYTLTTEKV--------PLLMNDVRVFFSSLYASVCPVVVIVSNRKVNRRL---SC-
Am-ORA2      GLWVYTLTVKQT------LSSALISDLRIFFSMLFAAISPIIIITTNMKVKKQL---L--
Fh-ORA2      GFWVYTLTVRNT------LSSSLISDLRVFFASLYAALSPLVIIASNRKVNGRL---RC-
Pf-ORA2      GLWVYTLTVRHT------MSSSLISDLRVFFASLYAALSPLVIIASNRKVNSRL---RC-
Xm-ORA2      CLWVYTLTLRHT------MSSSLISDLRVFFASLYAALSPLVIIVSNRKVNSRL---RC-
Gm-ORA2      GLWVHTLTVRRT------MGSSLVSDLRIFFSSLYAALSPAVIIATNRKVQRRL---RC-
Ga-ORA2      GLWVYTLTSRKA------MESSLISDLRVFFASLYAALSPAVVIASNRKVNSRL---RC-
Hc-ORA2      GLWVYTLTVKKT------MSSSLISDLRLFFGSLYAALSPLVIIASNRKVNSRL---GC-
On-ORA2      VLWVYTLTVKKT------MSSSLISDLRIFFGSLYAALSPLVIIASNRKVNSRL---GC-
Ss-ORA2      GLWVYTLTVRKT------MSSSLISDLRIFFSSLYAALSPLVIIATNRKVNSRL---RC-
Ol-ORA2      GLWVYTLTVKKT------MSSSLISDLRVFFASMYAALSPLVIIVSNRKVNSRL---MC-
Tn-ORA2      GLWVYTLTVREA------MASSLISEMRIFFSSMYAALSPIVIIVSNRKVNNIL---RC-
Tr-ORA2      GLWVYTLTVREA------MRSSLISDLRVFFSSLYAALSPIVIIISNRKVNSIL---RC-
Dr-ORA2      GLWVYTLTVSHT------LSSALITDLRLFFTSLYTAVSPLLILVSNTRL-------RC-
Lo-ORA2      GLWVYTLTVTQT------LSTSLISDLRIFFASLYAAVSPLVIIASNKKVKSQL---GC-
Am-ORA3      --WGASVISVNYFNYNRGASSTYLLVLARFFNSLFIALSPLILAVGHRRLRQFF---KS-
Fh-ORA3      --WGTSIISVNYFNYNRGSSAEYLLVIARFANIIFIAMSPVVLAFGHRRLRSCM---KS-
Pf-ORA3      --WGTSIISVNYFNYNRGSSAEFLLVIARFANIIFITMSPVVLAIGHRRLRSCM---KS-
Xm-ORA3      --WGTSIISINYFNYNRGSSAEFLLVVARFGNSIFITMSPVVLAIGHRRLRSCM---KS-
Ga-ORA3      --WGTSIISVNYFNYNRGSSAEYLLIIARFANIIFIAMSPIVLTFGHRRLRSFV---KS-
Tn-ORA3      --WGTSVISVNYFNYNQGSSAEFLLVIARFANIIFIALSPVVLAVGHRGLRSFF---KS-
Tr-ORA3      --WGTSIISVNYFNYNQGSSAEFLLVIARFANIIFIAMSPAVLAVGHRGLRTFF---KS-
Ol-ORA3a     --WGTSVISVNYFNYNRGTSSEFLLVIARFAQILFIALSPAVLAVGHRGLRSCI---KS-
Ol-ORA3b     --WGTSVISVNYFNYNRGTSSEFLLVIARFAQILFIALSPAVLAVGHRGLRSCI---KS-
Gm-ORA3      --WGTSIISVNYFNYNRGQSAEFLLVIARFANIFFIAMSPIILSIGHRRLRSFF---TS-
Hc-ORA3      --WGTSVISVNYFNYNRGSSADYLMVIARFANIIFIALSPVILAVGHRQLRSCI---KS-
On-ORA3      --WGTSVISVNYFNYNRGSSADYLLVIARFANIIFIALSPVVLAVGHRQLRSCI---RS-
Ss-ORA3a     --WGTSIISINYFNYNRGLSAGFLLVIARFANTIFIAISPIVLALGHRRLRAVI---KY-
Ss-ORA3b     --WGTSIISINYLNYYKGSSATFLPVIARFANSIFIAISPIVLALGHRRLRAVI---KS-
Dr-ORA3a     --WGTNVISVNYFNYNRGQSTEFLLIIARFVNMSFIAFSPIILAVGHRKLRAFI---KS-
Dr-ORA3b     --WGTSVISVNYFNYNRGSSTDWLLIAARIGNITFIALSPIVLAVGHRRLRAFL---AS-
Lo-ORA3      --WGASVLSVNYYNYNRGPSTEFLLVMARFTNSLFIAFSPLVLLAGHSRLKAIF---RV-
Am-ORA4      --WVLQVAAVTYYNYERGKHTDSLLTVSQFSSSLFVGFSPMVVALGHGKMRKKI---MG-
Fh-ORA4      --WVLQVAAVTYYNHDRGHHAEGLLTVAHFSASLFVGFSPLVVALGHGKLRRKI---KS-
Pf-ORA4      --WVLQVAAVTYYNHDGGQHAEGLLTVAHFSASLFVGFSPLVVALGHGKLRRKI---RS-
Xm-ORA4      --WVLQVAAVTYYNYDGGRHAEGLLTVAHFSASLFVGFSPLVVALGHGKLRRKI---RS-
Hc-ORA4      --WVLQVAAVTYYNHNRGHHAEGLLTVSHFSSSLFVGFSPLVVALGHGKLRRKI---MS-
On-ORA4      --WVLQVAAVTYYNHNRGHHVEGLLTVSHFSSSLFVGFSPLVVALGHGKLRRKI---IS-
Ss-ORA4      --WVLQVAAVTYYNHNRGNHAEELLTVAHFSASVFVGFSPMVVALGHGKLRRRI---MR-
Ol-ORA4      --WVLQVAAVTYYNHNRGLHAEGLLTVAHFSASTFVGFSPMVVALGHGKLRKKI---MG-
Tn-ORA4      --WVLQVAAVTYYNHDRGHHAEGLLTVAHFSASLFVGFSPLVVALGHSKLRRRI---SK-
Tr-ORA4      --WVLQVAAVTYYNHDGGHHAEGLLTVAHFSASLFVGFSPMVVALGHSKLRRRI---SS-
Gm-Ora4      --WVLQVAAVTYYNHDGGHHAEGLLTVAHFAASLFVGFSPMVVALGHGKLRKRI---VG-
Ga-ORA4      --WALQVAAVTYYNHDGGDHAEGLLTVAHFSASLFVGFSPMVVALGHGKLRRRI---MN-
Dr-ORA4      --WVLQVAAVTYYNHNRGAHAEGLLTVAHFSASLFVGFSPLVVALGHGKLRRRI---SG-
Lo-ORA4      --WGLQVTAVTHYNYNRGRQAETLLTVSHFAASVFVGFSPLVVALGHSKLRGRL---RR-
Am-ORA6      --CILYLARHLAFNL---YDFPAWSELEFFIATFYTALIPYVYGMGHNFFSLKHFR-RQ-
Dr-ORA6      --WSVYLLLHLAFDP---YSFPLWAEVEFFITTIYTALSPYVYGIGNDLFSIKRLY----
Fh-ORA6      --LGLHL-------P---VCVYVRGNLPRLVSAAETPRNSWSFAADVIATACPCGT-RR-
Pf-ORA6      --WTLYLILQWTFSP---SDCPIWIEIEFFISTSYMSISPYVYGIGGHLFSLENCK-HL-
Xm-ORA6      --WTLYLILQWTFSP---SDCPIWVEIEFFISASYMSISPYVYGIGGHLFSLENCK-HL-
Ga-ORA6      --WTLYLILQLTAVY---VDFAFRAEAELLISFSYTCISPYVYGIGNDLFSLKNFK-RN-
Hc-ORA6      --WTTYLIFQLAFNP---YEFPFWSEAQFFITISYTSISPYMYMIGHNMIPPHSCK-KG-
On-ORA6      --WTIYLIFQLAFNP---YEFLFWSEVQFFITISYTSISPYMYMIGHNMIPLHSCK-KG-
Tn-ORA6      --WTLNLIFQLTSSP---GAFTSGAEIKFFISSSYTAISPYVYGIGNNLFSLKKFR-KT-
Tr-ORA6      --WTVYLIFQLTISP---GDSSSWAEIKFFISTSYTSISPYVYGIGNNLFSLKKLR-KN-
Gm-ORA6      --WTMYLVFHLAFSP---VNVPLWGDIEFFITTSYTTLSPYVYGIGYDLFSLRYFI-KR-
Ol-ORA6      --WTFYLLFLFLLKP---NNLPSWREIEFFTLTSYSCFSPYVYGIGHNLFSLENFK-II-
Lo-ORA6      --WIMYLVLHLAFDS---SKMDNWSEIEFFIVTTYTTISPYVYGIGTNIFSCRQIV-KVL
Ss-ORA6      ------------------------------------------------------------
Am-ORA5a     --WLTHLLLSITSTI---HDSIVIHEMTSYIGALYTCIIPYIYLYGVKKLTCLTC--SSI
Am-ORA5b     --WVVHVILRLISTI---QESSLIMELASYIGAAYTCIIPYIYLHGVKKFSCTC---RG-
Fh-ORA5      --WVTHLLLRISNNI---QTSSLMMELASYIGASYTCIIPYIFLHGLKKLSCCR---KS-
Pf-ORA5      --WMTHLILRISNSI---QDSSLMMELASYIGAAYTCIIPYIFLHGVKKLSCSC---K--
Xm-ORA5      --WLTHLILRISNSM---QDSPLMMELASYIGAAYTCIIPYIFLHGVKKLSRLC---K--
Gm-ORA5      --WVTHLLLRISSNI---QTSPIVVEVASYIAASYTCIIPYIFLYGVKKLSCSCCGAKQ-
Ga-ORA5      --WLTHLLLRISNNI---HTSSMLVEVASYIAASYTCIIPYIFLYGVKKLGCPC---RR-
Tn-ORA5      --WLTHLLLRISNNV---HTSSVVVEVASYIAASYTCIIPYIFLHGVKKLYCSC---KR-
Tr-ORA5      --WLTHLLLRITNSV---HTSSLVVEVASYIAASYTCIIPYIFLHGVKKLHCSC---KR-
Hc-ORA5      --WLTHLLLHITNNI---HTSSIVVEVSSYIAASYTCIIPYIFLHGVKKLTCSS---KR-
On-ORA5      --WLTHLLLHITNNI---HTSSIVVEVSSYIAASYTCIIPYIFLHGVKKLTCSS---KR-
Ss-ORA5a     --WVTHLLLMMASNI---HTSSLVLELASYIGSSYTCIIPYIFLYGVKKLSCSC---RG-
Ss-ORA5b     --WVTHLLLRISSNI---HTSSVVVEVASYIASSYTCIIPYIFLYGVKKLSCSC---RR-
Ol-ORA5      --WLTHLLLHITNTV---HSSQSTLEVAGYITASYSCIIPYILLHGVKKLTCSH---KR-
Dr-ORA5      --WFIHLVLRIYSGF---RNSILVVKLTNFIGATYTCFVPYVYLHGVKKLNCSCCW----
Lo-ORA5      --WVTHLLLRIASNV---NESSAIVEIASYIAASYTCIIPFIFLHGVKKLSCRC---WK-
```

```
Am-ORA1     ---------------AAGG-EQKQ----------------------------DAAEDTDK
Fh-ORA1     ---------------AAEH-DQ-------------------------------PLVDTQE
Pf-ORA1     ---------------AAEH-DQ-------------------------------PLVDTQE
Xm-ORA1     ---------------VAEH-DQ-------------------------------PLVDTQE
Sc-ORA1     ------------------------------------------------------------
Sr-ORA1     ------------------------------------------------------------
Hc-ORA1     ---------------TAEH-EQ-------------------------------PSADTQD
On-ORA1     ---------------AAEH-EQ-------------------------------PSADTQD
Ol-ORA1     ---------------AAEQ-DQ-------------------------------PSVETQE
Ga-ORA1     ---------------AAEQ-DQ-------------------------------PSVDNQE
Ss-ORA1     ---------------VAADQEQ-------------------------------PSVNTQD
Dr-ORA1     ---------------ATSEQER-------------------------------QAEDGKN
Gm-ORA1     ---------------AVEQ-QQ-------------------------------SPVETQT
Lo-ORA1     ---------------AAANPEQ-------------------------------MSLNTED
Lo-ORA8a    ---------------CKGK-DQKH----------------------------LIAESTVS
Lo-ORA8b    ---------------CKGK-DQKH----------------------------LIAESTVS
Lo-ORA7     ---------------IKLA-----------------------------------------
Am-ORA2     ------------------------------------------------------------
Fh-ORA2     ---------------VAQE-RP------------------------------LLGKTARL
Pf-ORA2     ---------------VAQE-KP------------------------------LLGKTAHL
Xm-ORA2     ---------------VAQE-KP------------------------------LLGKTAHL
Gm-ORA2     ---------------GRGE-KH------------------------------RGESATEA
Ga-ORA2     ---------------DVKR-KP------------------------------VEEKDTCL
Hc-ORA2     ---------------VAHE-KS------------------------------AVEKIKNL
On-ORA2     ---------------VAHE-KS------------------------------AVEKTKNL
Ss-ORA2     ---------------VVQE-RP------------------------------VQDKATTL
Ol-ORA2     ---------------ISQK-LA--------------------------------------
Tn-ORA2     ---------------AGQE-KH------------------------------VQEKTT--
Tr-ORA2     ---------------AEQQ-KH------------------------------VQ------
Dr-ORA2     ---------------GKQP-ETMH------------------------------------
Lo-ORA2     ---------------MKTE-KG------------------------------PVSVDTVL
Am-ORA3     ---------------IISH-----------------------------------------
Fh-ORA3     ---------------SVSD-----------------------------------------
Pf-ORA3     ---------------SVSD-----------------------------------------
Xm-ORA3     ---------------SVSD-----------------------------------------
Ga-ORA3     ---------------TLSH-----------------------------------------
Tn-ORA3     ---------------LLAH-----------------------------------------
Tr-ORA3     ---------------LLSH-----------------------------------------
Ol-ORA3a    ---------------SLTY-----------------------------------------
Ol-ORA3b    ---------------SLTY-----------------------------------------
Gm-ORA3     ---------------LV-------------------------------------------
Hc-ORA3     ---------------TLVH-----------------------------------------
On-ORA3     ---------------TLVR-----------------------------------------
Ss-ORA3a    ---------------FLTH-----------------------------------------
Ss-ORA3b    ---------------FLTH-----------------------------------------
Dr-ORA3a    ---------------VLSHMI---------------------------------------
Dr-ORA3b    ---------------ILTHSIALCR------------------------HLWTYKLQK--
Lo-ORA3     ---------------IADHVHSFCF------------------------SQRCLKKSC--
Am-ORA4     ---------------MLQRWLRKALCR------DAEEHKRAPEISSTI-NSTITQKQTH-
Fh-ORA4     ---------------MILMWTDGPRGP------ESRSVSASPTTKGKQ-TCFEAQKEIQV
Pf-ORA4     ---------------MILVWTNVPLSQ------EAESGRKSPKTSGKQ-ISFVAQKEVKV
Xm-ORA4     ---------------LILMWTNVPHSQ------ETESGKKSLKTSGKK-PSFVAKKEVKV
Hc-ORA4     ---------------MMLG-----------------------------------------
On-ORA4     ---------------MMLG-----------------------------------------
Ss-ORA4     ---------------MIAGCADRVKCQQEKIIDESKAPDKRERTA-KQ-TVFTIQKEREV
Ol-ORA4     ---------------MILVWTKAFHCS------SKDTGRKRPSPKKKKLKVFDVQEEMRV
Tn-ORA4     ---------------VMLSWCRRLKGS------S--------NTKLMQIVFFVPQKQ---
Tr-ORA4     ---------------MMLGWCQCFKGR------SEDDP---PNTRAAKIISFVQHKQ---
Gm-Ora4     ---------------KLVGCSNVVRCRGHEGEDDGGAAGAAQQKEKGK-TVFVVQKEAAS
Ga-ORA4     ---------------MILMWSEVLKCRKENSEERIKSPKAKGRRG-KR-SVFIVQEERMV
Dr-ORA4     ---------------ILQSCMHRLKQTQDK-------------------PAEITEKDGRT
Lo-ORA4     ---------------MLRLHCGRREGPGGEDVGDRERDREAATKTPTT-TRTCRDKS---
Am-ORA6     ------------------------------------------------------------
Dr-ORA6     -C----------------------------------------------------------
Fh-ORA6     -------------RDGAAR-----------------------------------------
Pf-ORA6     ----------------LKR-----------------------------------------
Xm-ORA6     ----------------LKR-----------------------------------------
Ga-ORA6     ------------------------------------------------------------
Hc-ORA6     ------------------------------------------------------------
On-ORA6     ---SF--------KGTVSDFVEVI------------------------------------
Tn-ORA6     ------------------------------------------------------------
Tr-ORA6     ------------------------------------------------------------
Gm-ORA6     ------------------------------------------------------------
Ol-ORA6     ----------------RNKF----------------------------------------
Lo-ORA6     SCLSFSACGLYRQHSMCAKWNQ--------------------------------------
Ss-ORA6     ------------------------------------------------------------
Am-ORA5a    --------------D---------------------------------------------
Am-ORA5b    ------------------------------------------------------------
Fh-ORA5     ------------------------------------------------------------
Pf-ORA5     ------------------------------------------------------------
Xm-ORA5     ------------------------------------------------------------
Gm-ORA5     ------------------------------------------------------------
Ga-ORA5     ------------------------------------------------------------
Tn-ORA5     ------------------------------------------------------------
Tr-ORA5     ------------------------------------------------------------
Hc-ORA5     ------------------------------------------------------------
On-ORA5     ------------------------------------------------------------
Ss-ORA5a    ------------------------------------------------------------
Ss-ORA5b    ------------------------------------------------------------
Ol-ORA5     ------------------------------------------------------------
Dr-ORA5     ------------------------------------------------------------
Lo-ORA5     ------------------------------------------------------------
```

```
Am-ORA1         TNIK----------
Fh-ORA1         SNEK----------
Pf-ORA1         SNEK----------
Xm-ORA1         SNEK----------
Sc-ORA1         --------------
Sr-ORA1         --------------
Hc-ORA1         SNDK----------
On-ORA1         SNDK----------
Ol-ORA1         SNDK----------
Ga-ORA1         TSDK----------
Ss-ORA1         SNDKM---------
Dr-ORA1         SSGKN---------
Gm-ORA1         SNEK----------
Lo-ORA1         STRI----------
Lo-ORA8a        HLSPG--------Q
Lo-ORA8b        HLSPG--------Q
Lo-ORA7         --------------
Am-ORA2         --------------
Fh-ORA2         RSI-----------
Pf-ORA2         RSI-----------
Xm-ORA2         HSI-----------
Gm-ORA2         TAVST--------M
Ga-ORA2         STV-----------
Hc-ORA2         SSM-----------
On-ORA2         SSM-----------
Ss-ORA2         STV-----------
Ol-ORA2         --------------
Tn-ORA2         --------------
Tr-ORA2         --------------
Dr-ORA2         --------------
Lo-ORA2         STV-----------
Am-ORA3         --------------
Fh-ORA3         --------------
Pf-ORA3         --------------
Xm-ORA3         --------------
Ga-ORA3         --------------
Tn-ORA3         --------------
Tr-ORA3         --------------
Ol-ORA3a        --------------
Ol-ORA3b        --------------
Gm-ORA3         --------------
Hc-ORA3         --------------
On-ORA3         --------------
Ss-ORA3a        --------------
Ss-ORA3b        --------------
Dr-ORA3a        --------------
Dr-ORA3b        --------------
Lo-ORA3         --------------
Am-ORA4         -------------H
Fh-ORA4         IKVKA-KVKSQR--
Pf-ORA4         KKVKD-KVIPRR--
Xm-ORA4         KMVKD-KVMPHR--
Hc-ORA4         --------------
On-ORA4         --------------
Ss-ORA4         IK------------
Ol-ORA4         INIRECKALNDRRE
Tn-ORA4         --------------
Tr-ORA4         --------------
Gm-Ora4         TKEKT---MSKK--
Ga-ORA4         VQVKG-NAQADK--
Dr-ORA4         TQSAM-K-------
Lo-ORA4         --------------
Am-ORA6         --------------
Dr-ORA6         --------------
Fh-ORA6         --------------
Pf-ORA6         --------------
Xm-ORA6         --------------
Ga-ORA6         --------------
Hc-ORA6         --------------
On-ORA6         --------------
Tn-ORA6         --------------
Tr-ORA6         --------------
Gm-ORA6         --------------
Ol-ORA6         --------------
Lo-ORA6         --------------
Ss-ORA6         -------------R
Am-ORA5a        --------------
Am-ORA5b        --------------
Fh-ORA5         --------------
Pf-ORA5         --------------
Xm-ORA5         --------------
Gm-ORA5         --------------
Ga-ORA5         --------------
Tn-ORA5         --------------
Tr-ORA5         --------------
Hc-ORA5         --------------
On-ORA5         --------------
Ss-ORA5a        --------------
Ss-ORA5b        --------------
Ol-ORA5         --------------
Dr-ORA5         --------------
Lo-ORA5         --------------
```
